# A proposal of a new automated method for SfM/MVS 3D reconstruction through comparisons of 3D data by SfM/MVS and handheld laser scanners

**DOI:** 10.1371/journal.pone.0270660

**Published:** 2022-07-20

**Authors:** Akihiro Kaneda, Tomomi Nakagawa, Kohei Tamura, Koji Noshita, Hisashi Nakao

**Affiliations:** 1 Nara National Research Institute for Cultural Properties, Nara, Japan; 2 Anthropological Institute, Nanzan University, Nagoya, Japan; 3 Frontier Research Institute for Interdisciplinary Sciences, Tohoku University, Miyagi, Japan; 4 The Center for Northeast Asian Studies, Tohoku University, Miyagi, Japan; 5 Department of Biology, Kyushu University, Fukuoka, Japan; 6 Department of Anthropology and Philosophy, Nanzan University, Nagoya, Japan; Universidade do Vale do Rio dos Sinos, BRAZIL

## Abstract

SfM/MVS photogrammetry has received increasing attention due to its convenience, broadening the range of its applications into archaeology and anthropology. Because the accuracy of SfM/MVS depends on photography, one important issue is that incorrect or low-density point clouds are found in 3D models due to poor overlapping between images. A systematic way of taking photographs solve these problems, though it has not been well established and the accuracy has not been examined either, with some exceptions. The present study aims to (i) develop an efficient method for recording pottery using an automated turntable and (ii) assess its accuracy through a comparison with 3D models made by laser scanning. We recorded relatively simple pottery manufactured by prehistoric farmers in the Japanese archipelago using SfM/MVS photogrammetry and laser scanning. Further, by measuring the Hausdorff distance between 3D models made using these two methods, we show that their difference is negligibly small, suggesting that our method is sufficiently accurate to record pottery.

## Introduction

Based on recent technological developments, the application of 3D measurement has expanded. The field of archaeology has also benefited in various ways, ranging from the generation of more accurate records to the development of new analytical methods, in addition to digital preservation for disaster risk mitigation and uses in exhibitions. Examples include lithics, human skeletal remains, and architecture, and further potential applications have been discussed [1–11].

Among a wide variety of 3D measurement methods, Structure from Motion/Multi-View Stereo (SfM/MVS) photogrammetry has been widely used due to its functionality. This technique reconstructs a three-dimensional model of a focal object by matching a set of two-dimensional images or photos of the object and thus requires a low equipment cost for standard usage. Moreover, SfM/MVS technology usually generates RGM textured 3D model though many laser scanners including ones we used in this study cannot. SfM/MVS technology has become increasingly prevalent in archaeological and other practices including architecture, landscapes, drill cores, chambers in monuments, and pottery [12–22].

Some previous studies have reported that the accuracy of 3D models made with the SfM/MVS technology can be comparative with those made by laser scanning, though restricted to relatively large objects, without some exceptions [23–27]. Laser scanning is another method to construct a 3D model that has gradually become used in archaeological practices [28–32]. Because SfM/MVS reconstructs a 3D model based on matching images, the accuracy of the 3D model, particularly on the local scale, is still unclear. A 3D model obtained by laser scanning is therefore often of more stable quality than that obtained through SfM/MVS. Because it usually requires high equipment costs and can sometimes be difficult to obtain color information, complementary use with SfM/MVS has been attempted [20, 33–37].

While the convenience is an advantage, a crucial technical issue in archaeological applications using SfM/MVS technology is the proces of acquiring photographs or images. The results of SfM/MVS depend on the photos taken, i.e., a low coverage of the focal object or the low quality of photos could strongly decrease the accuracy of a 3D model. In addition, since SfM/MVS technology requires a large number of photos, several efficient methods of photography have been proposed, and some of these methods have used a turntable to rotate a target object [27, 38]. Furthermore, the method of photography depends on the nature of the target object. This means that efficient methods of photography should be adapted or developed for specific types of objects.

In the present study, we first propose a systematic method to record an object with the size of a relatively small jar. As we mentioned above, although a turntable has been used in the previous studies [27, 38], our approach uses a turntable controllable in an automated manner and thus can further economize time to record.

Then, we examine the accuracy of 3D models made with SfM/MVS by comparing them with those made by laser scanning as templates. As mentioned above, many previous studies using both methods have focused on relatively larger objects such as landscapes. Smaller objects such as pottery have often been measured with only one of these methods and the accuracy of the 3D models have not been examined without some exceptions [26, 27]. Pottery is prevalent in archaeological practice and thus it could be beneficial to investigate the accuracy of digital representations of pottery. In particular, because the previous studies have focused on large objects, the accuracy of its relatively deeper inside cannot be fully examined.

Given that the SfM/MVS technique of 3D model construction is more accessible than laser scanning, however, it is important to examine how efficiently and accurately we can produce 3D models of pottery through SfM/MVS. Especially in Japan, but surely in other regions as well, SfM/MVS has been employed for various kinds of archaeological remains in many academic and administrative excavations. These questions are urgent not only for researchers but also for professional archaeologists in the field of buried cultural heritage.

## Materials and methods

### Materials

**Fig 1:**
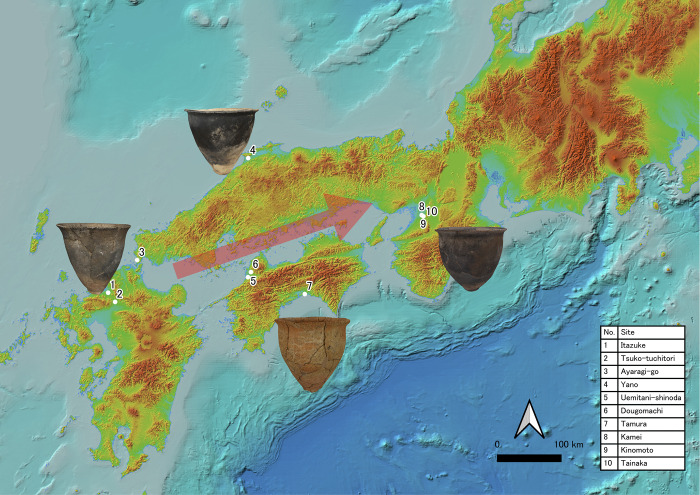
A supposed distribution route of Ongagawa pottery and the sites with samples used in the present study. 1. Itazuke (板付), 2. Tsuko-tsuchitori (津古土取), 3. Ayaragi-go (綾羅木郷), 4. Yano (矢野), 5. Uemitani-shinoda (上三谷篠田), 6. Dougomachi (道後町), 7. Tamura (田村), 8. Kamei (亀井), 9. Kinomoto (木の本), 10. Tainaka (田井中). This map contains data provided by Geospatial Information Authority of Japan (GSI: https://maps.gsi.go.jp/development/ichiran.html) as a part of The GSI Tiles Collection, the Elevation Map by Color. Especially, the sea area is based on the reports by Hydrographic and Oceanographic Department. Location information, scale, direction mark and some figures were added by TN with QGIS 3.16.11version. Reprinted from https://maps.gsi.go.jp/development/ichiran.html under a CC BY license, with permission from Geospatial Information Authority of Japan, original copyright 2013.

Our dataset is composed of 19 jars, all of which are categorized as the Ongagawa-style pottery of the Early Yayoi period (800−300 cal BC, see [39–41]), when rice farming was introduced to the Japanese archipelago from the Korean peninsula [42, 43]. The shapes and styles of this pottery are relatively simple and homogeneous. This pottery was distributed from the Kyushu area to the Tokai area in the Incipient and Early Yayoi period (Fig 1). Japanese archaeologists have suggested that the diffusion of the Yayoi culture, characterized by rice farming, accompanied this pottery [44–46]. All necessary permits were obtained for the described study from Fukuoka city, Ogori city, Shimonoseki city (Shimonoseki City Archaeological Museum), Izumo city (Izumo Yayoinomori Museum), Ehime prefecture, Kochi prefecture, and Osaka prefecture, which complied with all relevant regulations.

### Reconstruction equipment

**Fig 2:**
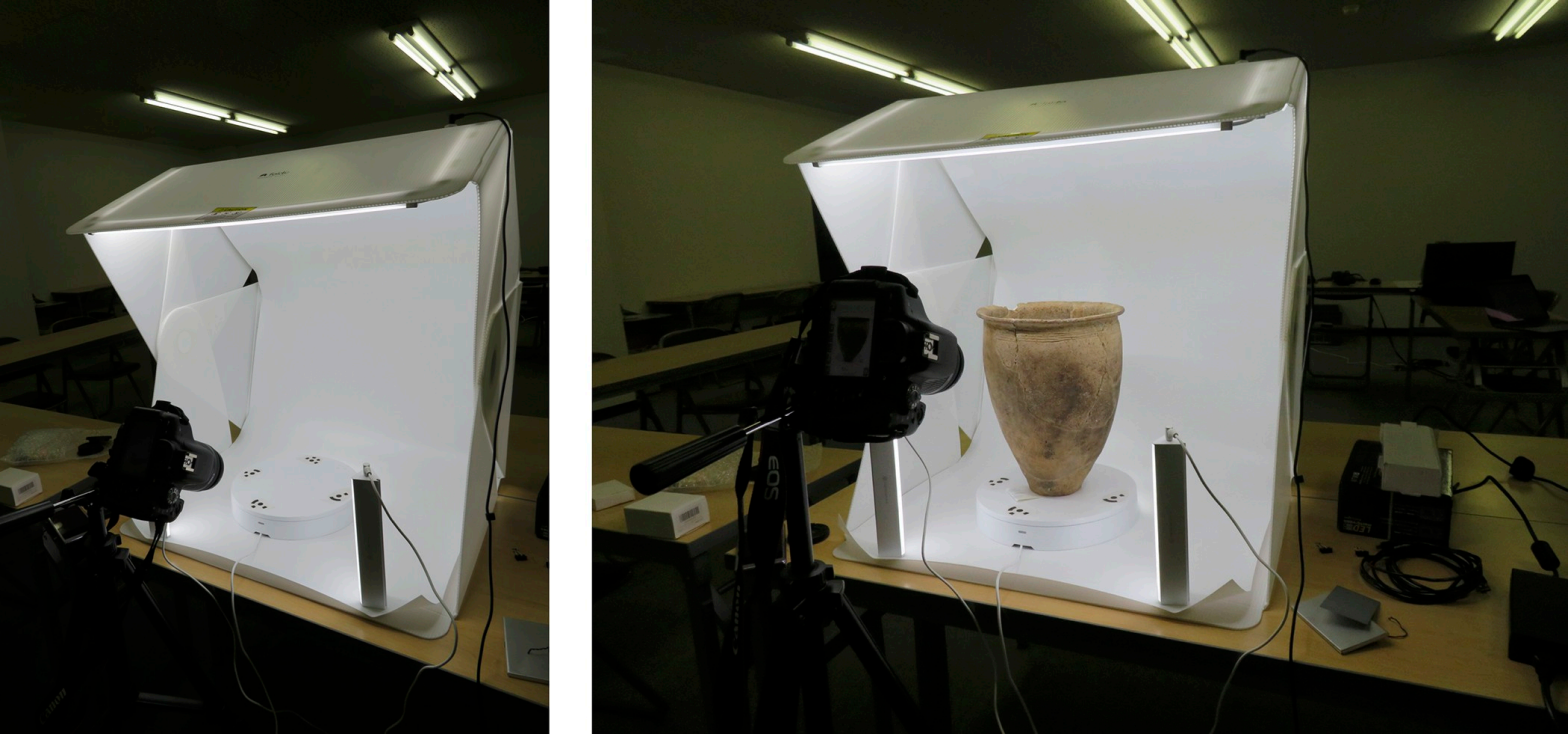
Placement for SfM/MVS construction of 3D models in FOLDIO 3. This pottery is from the Dougomachi site, owned by The Ehime Research Center for Buried Cultural Properties.

**Fig 3:**
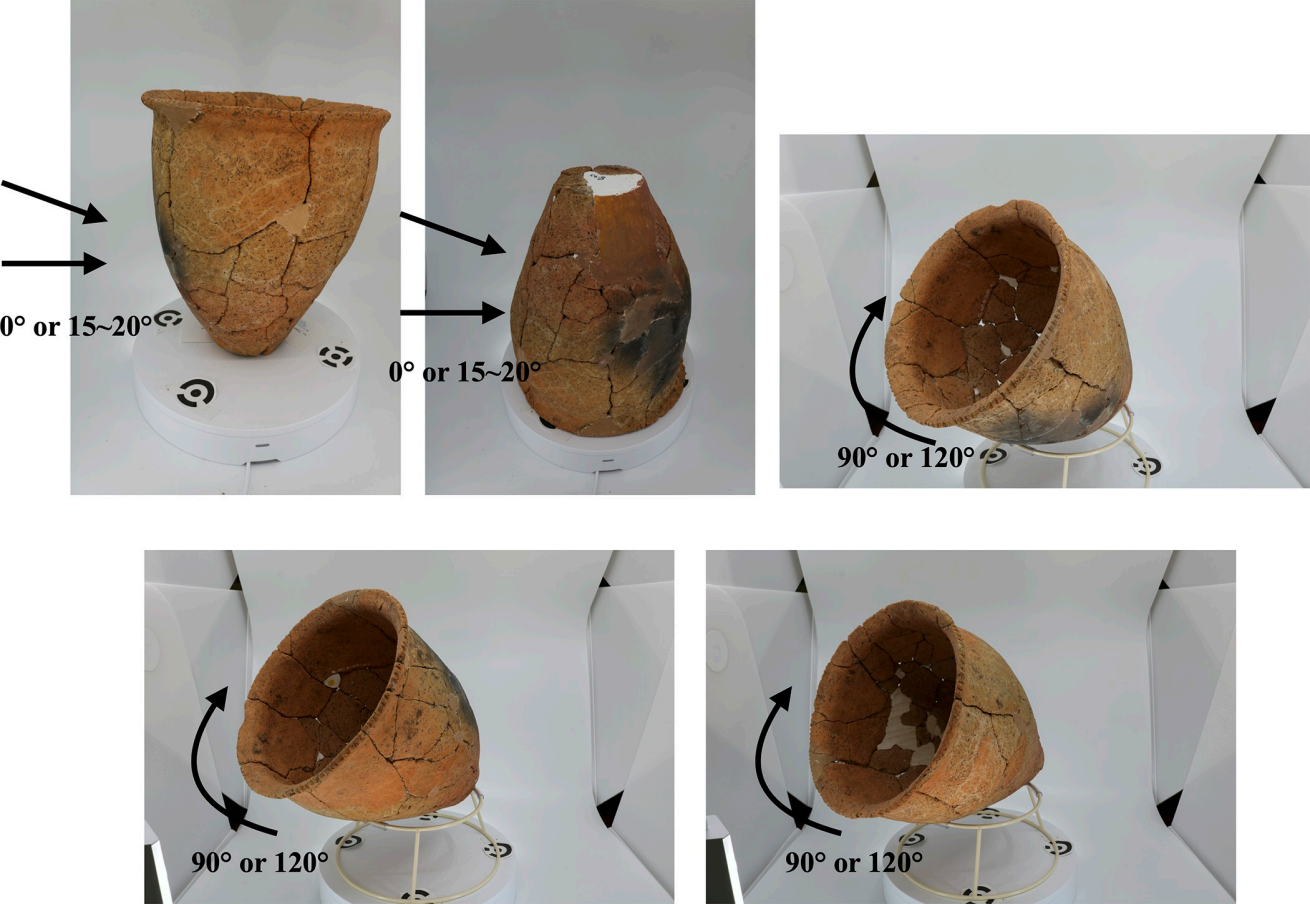
Sample photos from different angles for SfM/MVS. The number of angles (three or four) when we take photos of the inside of pottery depends on its form. It also depends on the outside form whether we tilt the camera at an angle of 15 to 20 degrees or take photos from front.

We used two different methods: laser scanning and SfM/MVS to reconstruct 3D models of Ongagawa-style jars. Our system of photography is described below. For a photo studio set, we used the FOLDIO 3 by ORANGEMOKIE (https://orangemonkie.com/), which includes a turntable, four lights, and a white or black background (Fig 2). The turntable can be controlled by smartphone and rotates in 15 degree increments (the degree can be set via smartphone). A camera can synchronize with the turntable via the smartphone and photos are taken automatically at each angle. The setting could quickly produce photos with better quality from the same angles and distances, which finally lead to better point cloud (see Discussion for more details). We pasted target marks, which are included in Metashape, on the turntable. We situated a jar on the turntable in five or six different positions (Fig 3, see also S1 Video). We took photos of the inside of each jar in three or four positions and the outside in two to four positions. As a result, we obtained 120 or 192 photos per jar, with one exceptional case of 299 photos. We used three models of cameras (Sony α6500, Cannon Eos Kiss X8i, and Kiss 7). The setting of each camera in many cases is as follows: ISO sensitivity is from 100 to 200, F number from 13 to 18, and shutter speed from 1/100 to 1/40. Optical lenses used are attached standard ones. We also took photos in JPEG and RAW files, and 3D models are finally constructed from RAW files.

To construct a 3D model from a set of photos, we used Agisoft Metashape 1.6.5 (https://www.agisoft.com/). In the construction process, we selected the ‘high’ options in ‘Align Photos’, ‘Build Dense cloud’, and ‘Build Mesh’, after confirming that a 3D model could be successfully constructed from our set of photos with the above settings on ‘low’. Photography and confirmation of the 3D model took about 30 minutes, respectively. We also spent 40 minutes for the final construction of the 3D model. After a 3D model is constructed, we minimized the distance errors between scale markers. It should be noted that an advantage of the above equipment is the lower costs. The price of each camera is around 1,000 US$ and that of the photo studio is under 400 US$.

**Fig 4:**
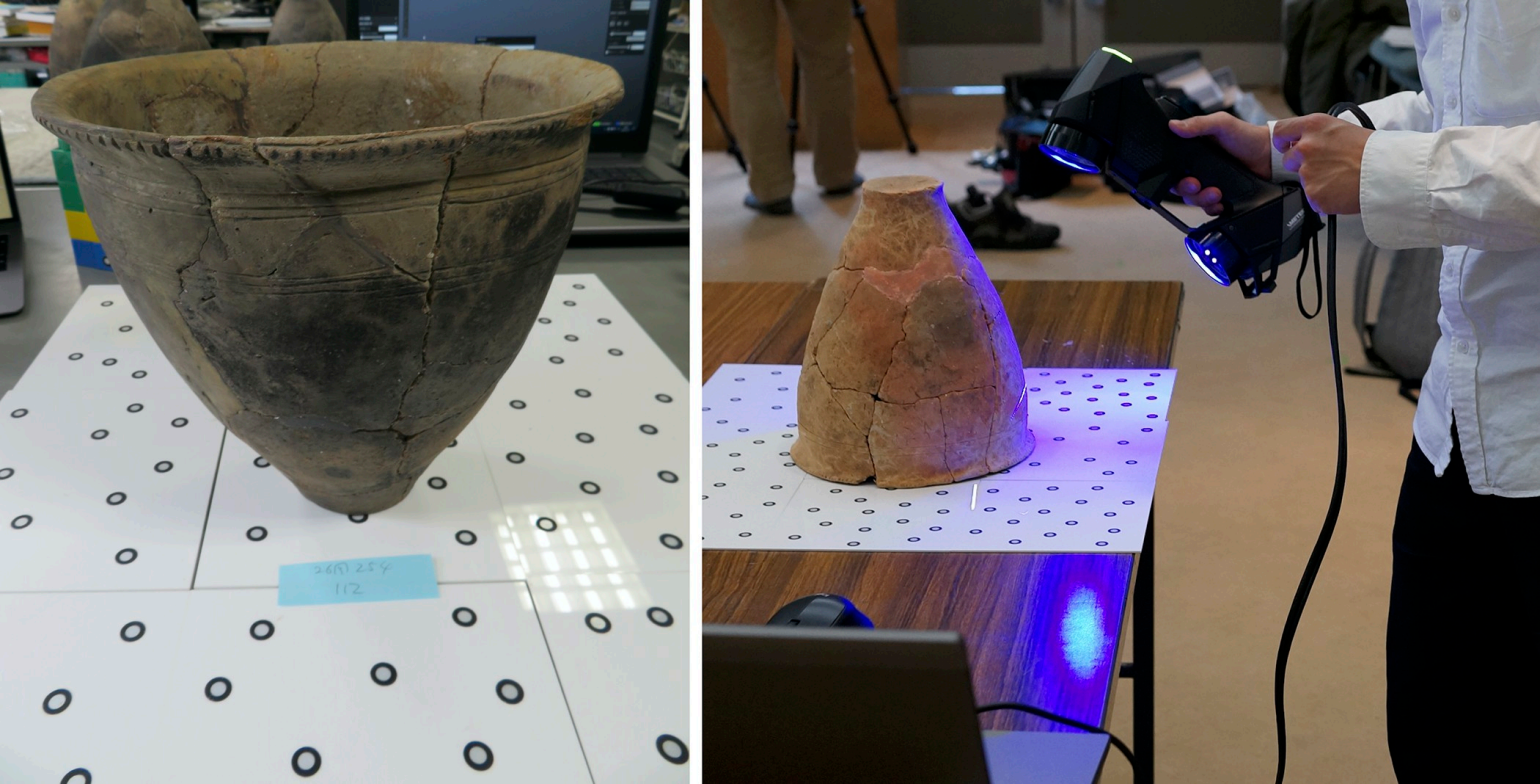
Placement for scanning with the Creafrom HandySCAN BLACK or Creafrom HandySCAN BLACK™ | Elite (the pottery is tainaka_26_254 and ayaragi-go_407). The scanner reconstructs 3D models of the pottery based on the time and angle of the reflected laser from the target points (silver and black seals on the acrylic boards).

**Fig 5:**
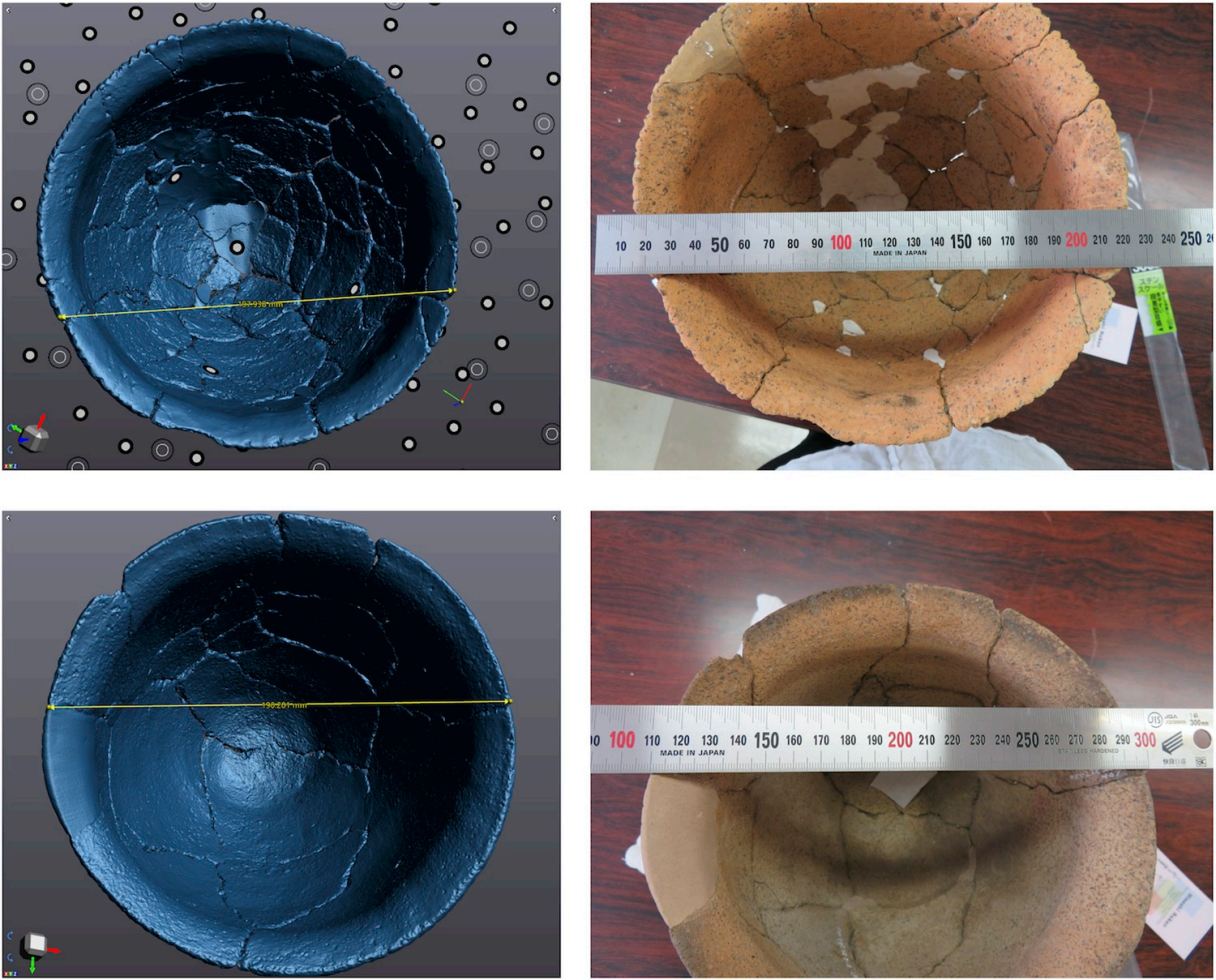
Comparisons of the distances in the 3D models by Creafrom HandySCAN BLACK™ | Elite and the actual objects using a steel ruler.

We used two versions of laser scanners from Creaform (LS1: Creafrom HandySCAN BLACK™ | Elite; and LS2: Creafrom HandySCAN BLACK). These laser scanners calculate distance based on the triangulation method: 3D models are reconstructed through the reflection of the laser from target points (Fig 4). Although a standard use assumes that target points are put on the surface of the focal object, this is not always feasible or allowed. Thus, we placed the target points on acrylic boards, which, as a result, was quicker than putting target points on each object. The results by our way and standard way are not significantly different (see S1 Fig). The time needed for reconstruction was almost 40 minutes with the Creafrom HandySCAN BLACK and 15 minutes with the Creafrom HandySCAN BLACK™ | Elite. It also took 40 minutes and 10 minutes, respectively, for cleaning the raw data and constructing the full 3D model. The model accuracy is 0.025 mm (https://www.creaform3d.com/), which is supported by comparisons between scanned data and the actual scale (Fig 5). Advantages of the above laser scanning are that a 3D model is accurately and speedily obtained and the scanners are mobile. On the other hand, the scanners cost over 60,000 US$. 3D models of some jars were reconstructed using all five instruments (three cameras and two laser scanners), while others were reconstructed using only a selection (see Table 1).

**Table 1:** Summarized data of comparisons. Pottery from the Ayaragi go site is owned by the Shimonoseki city archaeological museum, the Tamura site by the Kochi Prefecture Archaeological Center, the Itazuke site by the Fukuoka City Archaeological Center, the Kamei site by the Osaka Center for Cultural Heritage, the Kinomoto and Tainaka site by the Osaka Prefectural Board of Education, the Yano site by the Izumo Yayoinomori Museum, the Uemitani and Dougomachi site by the Ehime Prefecture Archaeological Center, the Tsuko-tsuchitori site by the Ogori City Archaeological Center, and the Shimokawatsu site by the Kagawa Prefecture Archaeological Center.

				Mesh numbers		Deviations (3σ, mm)					
Comparison	No.	Site	Date	L(1)	L(2)	max	min	mean	Rim diameter (mm)	max/Rim	min/Rim
**L(1)/L(2)**	ayaragi-go_293	Ayaragi-go	2020/2/20	5266582	5304773	0.46	-0.51	-0.03	174.94	0.26%	0.29%
	tamura_c1_34_12	Tamura	2020/7/14	10158956	10308728	0.63	-0.63	0.00	190.02	0.22%	0.33%
	tamura_c1_12_7	Tamura	2020/7/14	8799406	8799982	0.36	-0.36	0.00	202.73	0.12%	0.18%
	Mean			8074981	8137828	0.48	-0.50	-0.03		0.20%	0.27%
				**L(1)**	**SfM(1)**						
**L(1)/SfM(1)**	itazuke_35_3_161_1	Itazuke	2020/9/1	12956257	1740874	0.54	-0.45	0.04	210.3	0.26%	0.21%
	itazuke_36_1_50_12	Itazuke	2020/9/2	13862297	2598776	0.52	-0.41	0.05	242.19	0.21%	0.17%
	kamei_84_490	Kamei	2020/8/3	9813974	2010834	0.49	-0.49	-0.01	221.16	0.22%	0.22%
	kinomoto_55_326	Kinomoto	2020/7/28	4705671	1150498	0.36	-0.38	-0.01	142.18	0.25%	0.27%
	kinomoto_76_709	Kinomoto	2020/7/28	7688935	1701894	0.32	-0.32	0.00	221.94	0.14%	0.14%
	tainaka_17_156	Tainaka	2020/7/27	11985939	3324066	0.29	-0.25	0.01	221.11	0.13%	0.11%
	tainaka_26_254	Tainaka	2020/7/27	10809609	2748654	0.48	-0.43	0.02	232.46	0.21%	0.18%
	tamura_b1_4_8	Tamura	2020/7/14	11851043	2743404	1.15	-1.17	-0.03	211.49	0.54%	0.55%
	tamura_c1_34_12	Tamura	2020/7/14	10158956	2192924	0.95	-0.92	0.01	190.02	0.50%	0.48%
	tamura_c1_12_7	Tamura	2020/7/14	8799406	2044780	0.87	-0.76	0.05	202.73	0.43%	0.37%
	tamura_e2_9	Tamura	2020/7/14	8903216	2207884	0.98	-1.00	-0.02	202.73	0.48%	0.49%
	yano_244_9	Yano	2020/7/31	13090331	2411996	0.37	-0.37	0.00	234.87	0.16%	0.16%
	yano_256_3	Yano	2020/7/31	9318379	1771810	0.31	-0.35	-0.02	209.47	0.15%	0.17%
	uemitani_117	Uemitani	2021/1/12	11554144	3277332	0.45	-0.39	0.02	211.61	0.21%	0.18%
	dougomachi_5	Dougomachi	2021/1/12	7097722	2453772	0.42	-0.27	0.02	187.52	0.22%	0.14%
	Mean			10173059	2291967	0.57	-0.50	0.02		0.28%	0.26%
				**L(1)**	**SfM(2)**						
**L(1)/SfM(2)**	ayaragi-go_293	Ayaragi-go	2020/2/20	5266582	2664648	0.48	-0.51	-0.03	174.94	0.27%	0.29%
	tamura_b1_4_8	Tamura	2020/7/14	11851043	4206880	0.71	-0.58	0.05	211.49	0.34%	0.27%
	tamura_c1_34_12	Tamura	2020/7/14	10158956	3045276	0.75	-0.68	0.02	190.02	0.39%	0.36%
	tamura_c1_12_7	Tamura	2020/7/14	8799406	3588412	0.66	-0.64	0.00	202.73	0.33%	0.32%
	tamura_e2_9	Tamura	2020/7/14	8903216	2645754	0.49	-0.42	0.03	189.77	0.26%	0.22%
	tsuko_74_1	Tsuko-tsuchitori	2020/7/10	7274078	1607166	0.31	-0.24	0.04	176.69	0.18%	0.14%
	tsuko_89_2	Tsuko-tsuchitori	2020/7/10	10553828	2480806	1.05	-1.02	0.00	203.48	0.52%	0.50%
	yano_256_3	Yano	2020/7/31	9318379	410004	0.36	-0.55	-0.10	209.47	0.17%	0.26%
	shimokawatsu_17_7	Shimokawatsu	2020/6/19	16710170	2729402	1.04	-0.90	0.05	224.61	0.46%	0.40%
	Mean			9870629	2597594	0.65	-0.62	0.00		0.32%	0.31%
				**L(1)**	**SfM(3)**						
L(1)/SfM(3)	tamura_b1_4_8	Tamura	2020/7/14	11851043	2894552	0.67	-0.64	0.00	211.49	0.32%	0.30%
	tamura_c1_34_12	Tamura	2020/7/14	10158956	2330682	0.92	-0.97	-0.04	190.02	0.48%	0.51%
	tamura_c1_12_7	Tamura	2020/7/14	8799406	1879376	0.72	-0.77	-0.04	202.73	0.36%	0.38%
	Mean			10269802	2368203	0.77	-0.79	-0.03		0.39%	0.40%

We compared their shapes using GOM Inspect (https://www.gom-inspect.com/). GOM Inspect can easily fit the two models and calculate the distance between them. First, after we initially aligned the two models by ‘3-point alignment’ (the 3 points are artificially selected though the results are not substantially different when we select different points), we selected the surface of the models and then used ‘Local best-fit’ command as main alignment. Next, we compared the two kinds of models by ‘Create surface comparison on actual’ and created the color legends and histograms. Finally, we calculated maximum and mean deviations (3σ), which is summarized in Table 1.

## Results

### Comparison of 3D models by laser scanning: L (1) and L (2)

**Fig 6:**
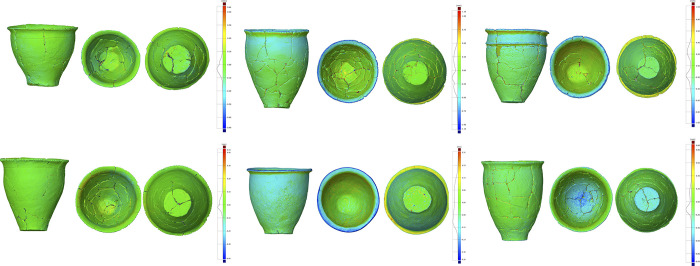
The difference between 3D models by two kinds of laser scanners (ayaragi-go_293 and tamura_c1_12_7), laser scanner (1) and SfM (1) (tamura_b1_4_8 and yano_256_3), laser scanner (1) and SfM (2) (tsuko_89_2), and laser scanner (1) and SfM (3) (tamura_c1_34_12) using GOM Inspect. For other results, see S2–S32 Figs.

The results of the comparisons are summarized in Table 1. Fig 6 shows the difference between two 3D models (ayaragi-go_293 and tamura C1_12_7) by two kinds of laser scanners examined with GOM Inspect. The negative or positive values of deviations between two models mean that meshes of the compared model are located inside or outside of the reference model. GOM Inspect indicates that the mean error is -0.03mm. The results shows that the 3D models created by the two laser scanners are almost identical.

### Comparison of 3D models by laser scanning and SfM: L (1) and SfM (1)

The morphological differences between 3D models by L(1) and SfM (1) were also examined by GOM Inspect (Table 1 and Fig 6 (tamura_b1_4_8 and yano_256_3), see S2–S32 Figs for more information). The mean error is 0.02 mm and the greatest difference was found around the rim of the pottery. Given that the diameters of the pottery rims are around 200 mm, it is reasonable to say that the mean error is not significant.

### Comparison of 3D models by laser scanning and SfM: L (1) and SfM (2)

The comparative results by L(1) and SfM (2) were also summarized in Table 1 and Fig 6 (tsuko_89_2). The mean error is 0.00 mm and the maximum and minimum deviations are under 0.4% of the rim diameters, and so we could say that they are not significant differences.

### Comparison of 3D models by laser scanning and SfM: L (1) and SfM (3)

The results in Table 1 and Fig 6 (tamura_c1_34_12) show that the mean error is -0.03 mm. The maximum deviation is not over 0.5% of the rim diameters, which is not serious for ordinary archaeological research.

## Discussion

In the present study, we proposed a new and automated method for SfM/MVS photogrammetry. Our results indicate that the accuracy of the above method is comparable with that of laser scanning, suggesting that our method can provide a reliable 3D model.

**Fig 7:**
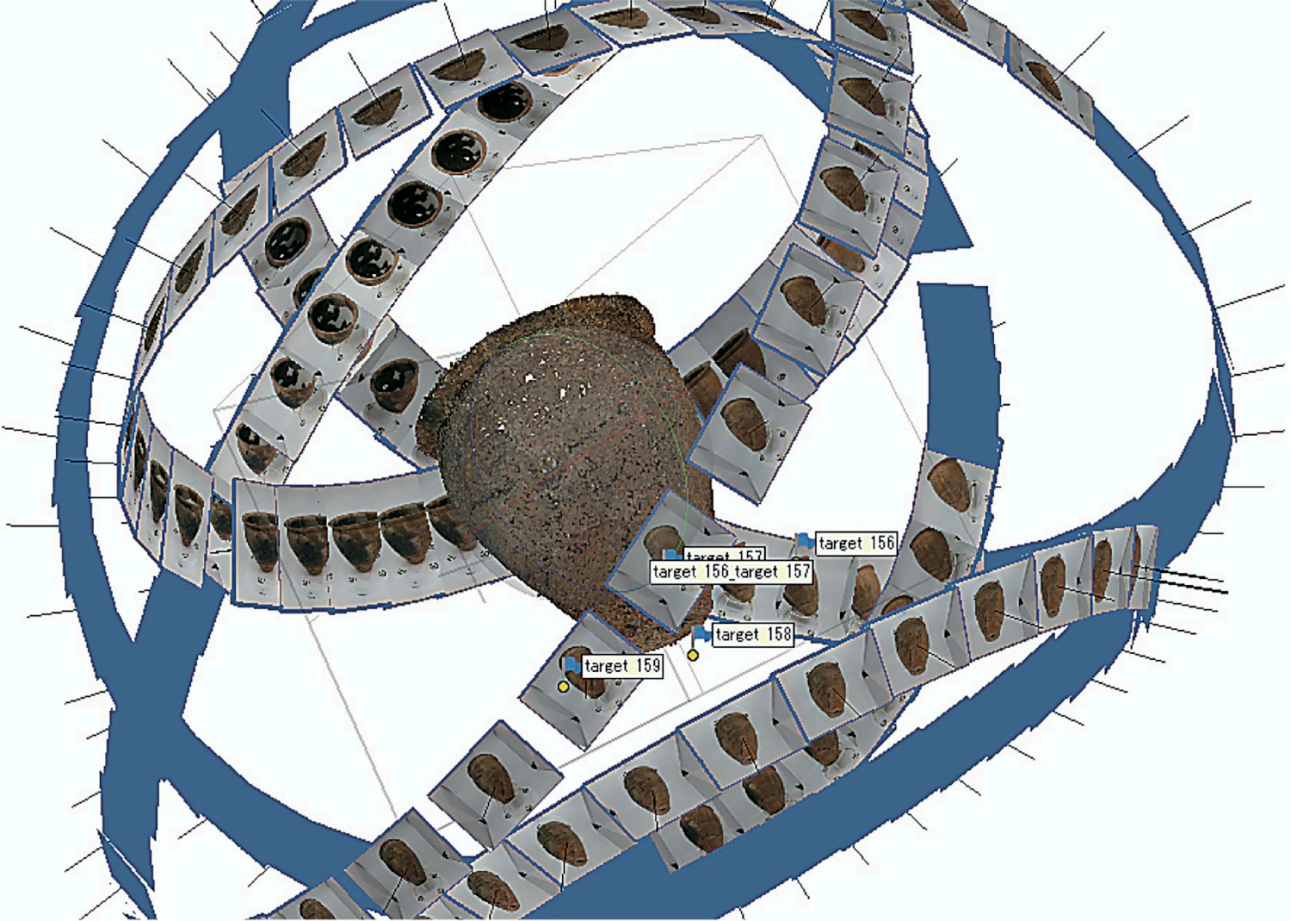
Photos aligned in Metashape.

As mentioned briefly in Materials and Methods, the reasons why the above method is effective for constructing sufficiently accurate 3D models are as follows. (1) Automatically controllable turntables enable us to release the shutter without camera shake, which makes photos clearer and less blurry. (2) Because we could take photos from the same angles and distances and their overlapped areas are also the same, point cloud produced from the photos through Metashape ranges uniformly (Fig 7). (3) The objects turn but the background is the same, and so photos alignment does not depend on the background. Even when we ourselves turn around the objects to take photos, (1) and (3) are still possible though (2) is rather difficult.

Note that while we have to turn and reconstruct 3D models of the objects manually when handheld laser scanning, in SfM/MVS, photos could be taken almost automatically after an object is set up in our method using a turntable, allowing us to reconstruct 3D models of multiple objects at the same time with multiple devices. One might claim that common stationary laser scanners also could reconstruct 3D models of the objects with automatic turntables, which also enable us to reconstruct 3D models of multiple objects at the same time. Importantly, our methods use relatively cheaper devices: The price of each camera is around 1,000 US$ and that of the photo studio is under 400 US$. The total prices are still less expensive than common and high-resolution stationary laser scanners (e.g., ArticEVA, ATOS, EinScan, and etc.). Scalability and affordability of our methods are better than such common laser scanners.

Although the time needed for 3D model construction is still a bit longer than that needed for laser scanning, as a whole, we can conclude that our method can be as efficient and reliable as laser scanning for the construction of 3D models of pottery. However, we admit that the above conclusion depends on the aim of a 3D model and the kinds of objects. For example, to observe detailed patterns or decorations on an artifact, a 3D model with higher resolution, in other words a greater number of photos taken at close range, may be required and thus our method may be insufficient. To obtain photos of the inside of an object with a narrow rim like a pot, the SfM/MVS technique may be less effective. Additionally, an object larger than the size of the FOLDIO 3 is out of the scope of our method. An extension of the current study is developing a device for measuring a larger object with multiple cameras. As some previous studies focusing on lithics [8] have suggested, the efficient and effective settings for 3D model construction with SfM/MVS depend on the kinds and properties of the objects to be reconstructed. Applying the current method and its extension to various objects, exploring appropriate instruments and settings, can contribute to expanding the range of application of the SfM/MVS technique.

## Conclusion

In the present study, we proposed an efficient method of SfM/MVS photogrammetry. By comparing 3D models constructed through both SfM/MVS and laser scanning, we show that their 3D models are almost identical, suggesting the accuracy of our method. We conclude that SfM/MVS can be reliable, as well as expedient, for recording pottery.

We should mention our method’s limits and possibilities for improvements. As we have already pointed out, this study used a box of photo studio and automatic turntable, and our method should be modified when applied to the objects that are larger than the box or exceed the turntable withstand load. The method is also not efficient for reconstructing insides of jars with narrower necks. We could modify the system to use two cameras simultaneously controlled by two smartphones, which makes the procedures more efficient.

Our method is potentially applicable to other archaeological remains, at least similar with pottery in size. We have actually confirmed that our method is useful for human skeletal remains [47]. This study employs the almost same method and shows that two kinds of results by laser scanning and SfM/MVS photogrammetry using our methods are not significantly different. We believe that such automated procedures are useful for reconstructing 3D models of various kinds of materials, and future work should be conducted on more diverse objects in terms of size and shape.

## Supporting information

S1 Fig A comparative result of 3D models by a standard way and our way of laser scanning. (PDF)

S2 Fig A comparative result of 3D models by two laser scanners (ayaragi-go_293). (PDF)

S3 Fig A comparative result of 3D models by two laser scanners (tamura_C1_12_7). (PDF)

S4 Fig A comparative result of 3D models by two laser scanners (tamura_C1_34_12). (PDF)

S5 Fig A comparative result of 3D models by L (1) and SfM (1) (dougoimaichi_5). (PDF)

S6 Fig A comparative result of 3D models by L (1) and SfM (1) (itazuke_35_3_161_1). (PDF)

S7 Fig A comparative result of 3D models by L (1) and SfM (1) (itazuke_36_1_50_12). (PDF)

S8 Fig A comparative result of 3D models by L (1) and SfM (1) (kamei_84_490). (PDF)

S9 Fig A comparative result of 3D models by L (1) and SfM (1) (kinomoto_55_326). (PDF)

S10 Fig A comparative result of 3D models by L (1) and SfM (1) (kinomoto_76_709). (PDF)

S11 Fig A comparative result of 3D models by L (1) and SfM (1) (tainaka_17_156). (PDF)

S12 Fig A comparative result of 3D models by L (1) and SfM (1) (tainaka_26_254). (PDF)

S13 Fig A comparative result of 3D models by L (1) and SfM (1) (tamura_B1_4_8). (PDF)

S14 Fig A comparative result of 3D models by L (1) and SfM (1) (tamura_c1_12_7). (PDF)

S15 Fig A comparative result of 3D models by L (1) and SfM (1) (tamura_c1_34_12). (PDF)

S16 Fig A comparative result of 3D models by L (1) and SfM (1) (tamura_e2_13_9). (PDF)

S17 Fig A comparative result of 3D models by L (1) and SfM (1) (uemitani). (PDF)

S18 Fig A comparative result of 3D models by L (1) and SfM (1) (yano_244_9). (PDF)

S19 Fig A comparative result of 3D models by L (1) and SfM (1) (yano_256_3). (PDF)

S20 Fig A comparative result of 3D models by L (1) and SfM (2) (ayaragi-go_293). (PDF)

S21 Fig A comparative result of 3D models by L (1) and SfM (2) (shimokawatsu_17_7). (PDF)

S22 Fig A comparative result of 3D models by L (1) and SfM (2) (tamura_B1_4_8). (PDF)

S23 Fig A comparative result of 3D models by L (1) and SfM (2) (tamura_c1_12_7). (PDF)

S24 Fig A comparative result of 3D models by L (1) and SfM (2) (tamura_c1_34_12). (PDF)

S25 Fig A comparative result of 3D models by L (1) and SfM (2) (tamura_e2_13_9). (PDF)

S26 Fig A comparative result of 3D models by L (1) and SfM (2) (tsuko_74_1). (PDF)

S27 Fig A comparative result of 3D models by L (1) and SfM (2) (tsuko_89_2). (PDF)

S28 Fig A comparative result of 3D models by L (1) and SfM (2) (yano_256_3). (PDF)

S29 Fig A comparative result of 3D models by L (1) and SfM (3) (tamura_B1_4_8). (PDF)

S30 Fig A comparative result of 3D models by L (1) and SfM (3) (tamura_c1_12_7). (PDF)

S31 Fig A comparative result of 3D models by L (1) and SfM (3) (tamura_c1_34_12). (PDF)

S32 Fig A comparative result of 3D models by L (1) and SfM (2) (tamura_c1_34_12). (PDF)

S1 Video A sample movie of photo shooting for SfM/MVS. (MP4)
