# Supplementary figures and images for: A proposal of a new automated method for SfM/MVS 3D reconstruction through comparisons of 3D data by SfM/MVS and handheld laser scanners
Source: PLoS One. 2022 Jul 20;17(7):e0270660. doi: 10.1371/journal.pone.0270660 (PMC9299387; doi:10.1371/journal.pone.0270660)

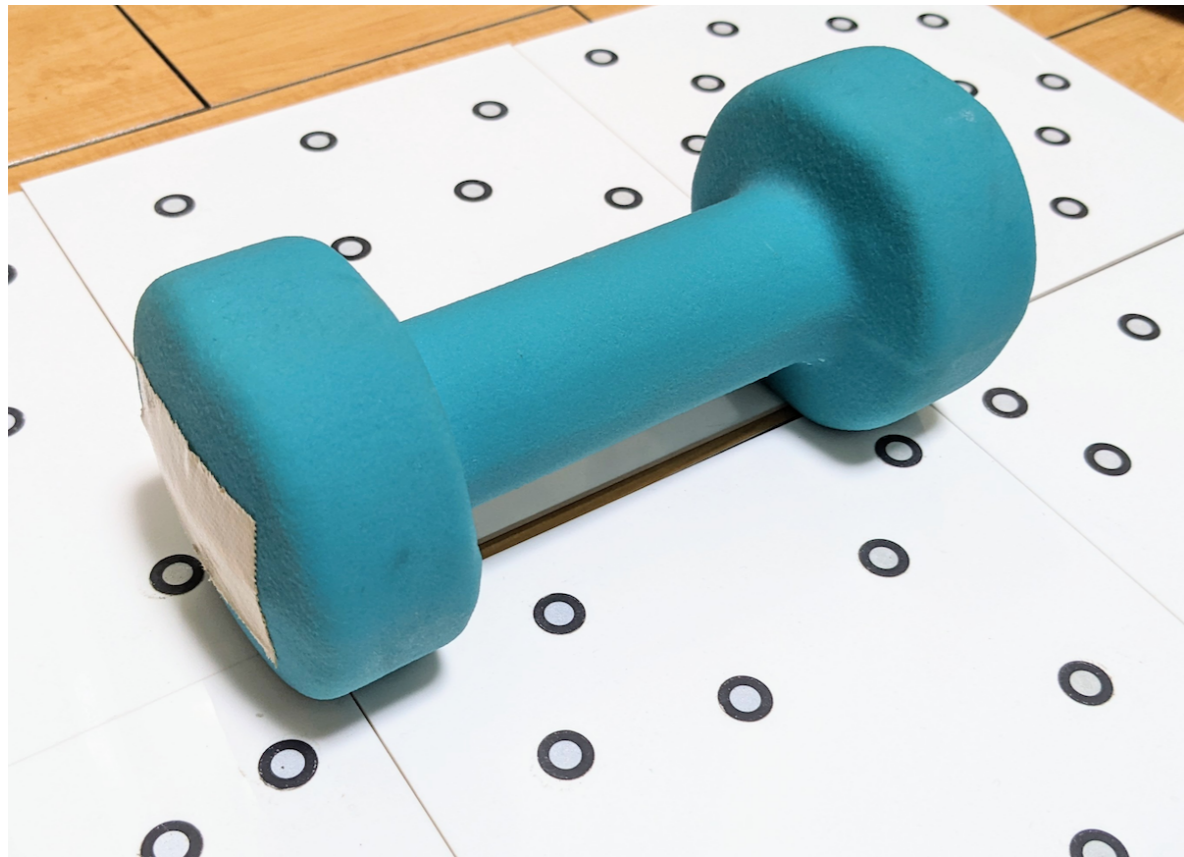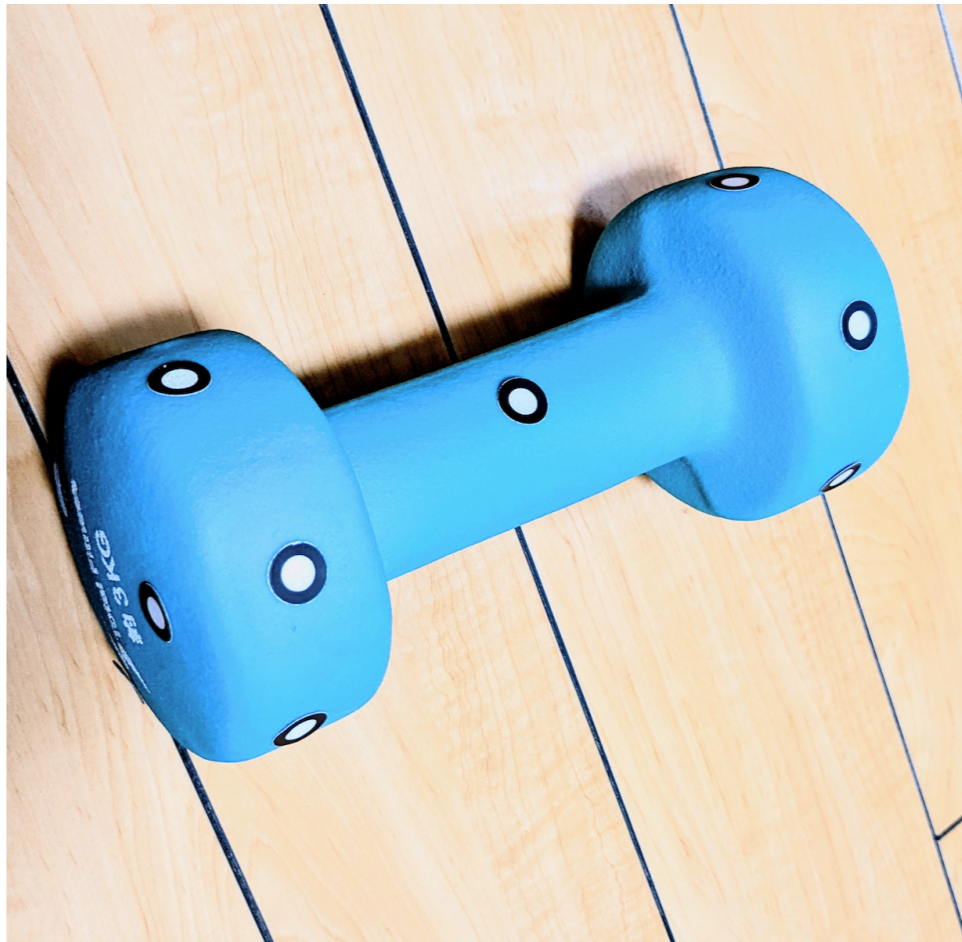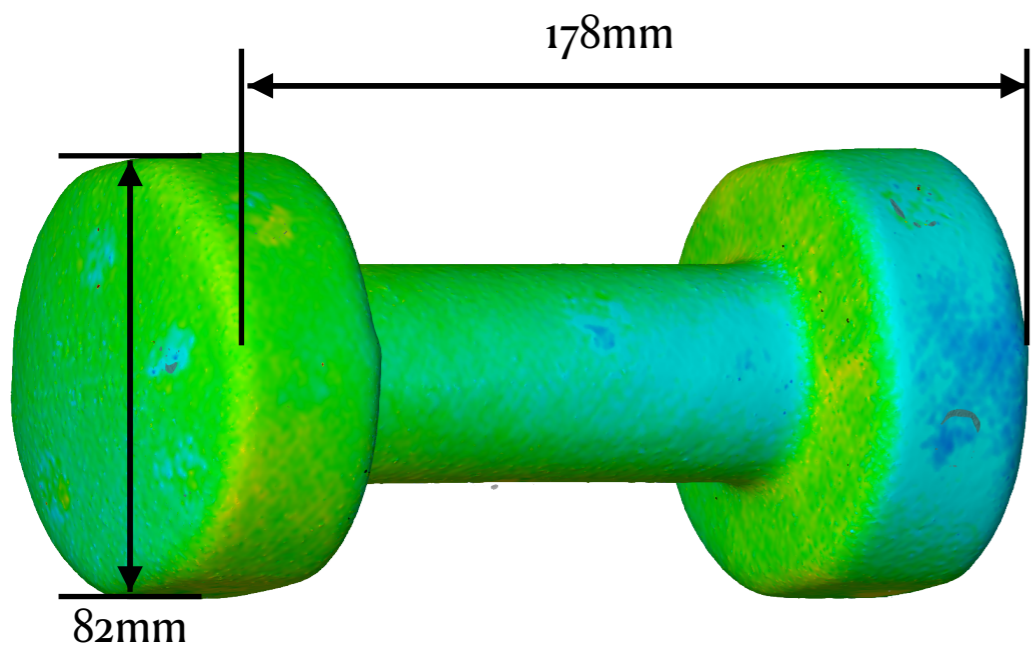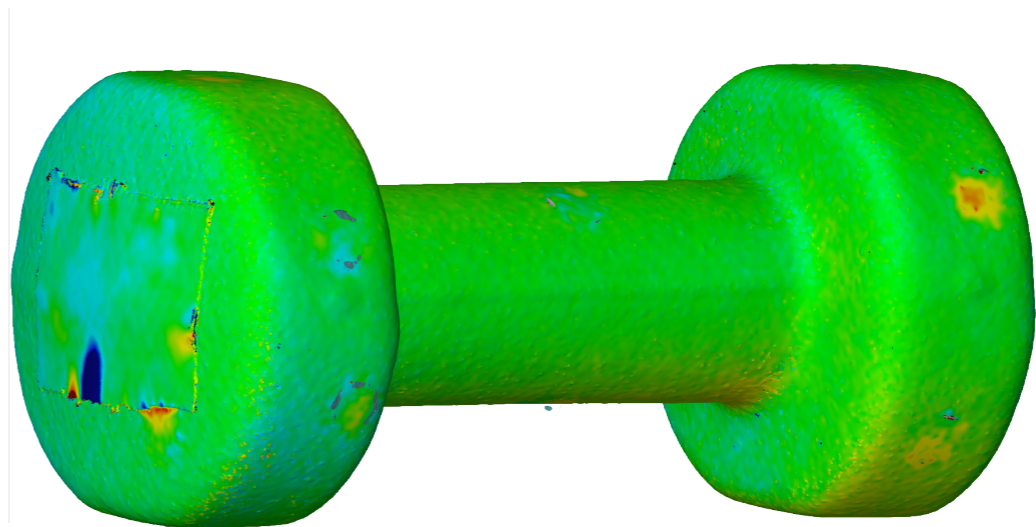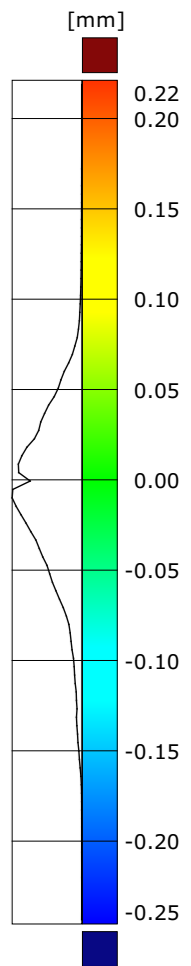

Supplement: S1 Fig — (PDF) [file pone.0270660.s001.pdf]

タイトルなし

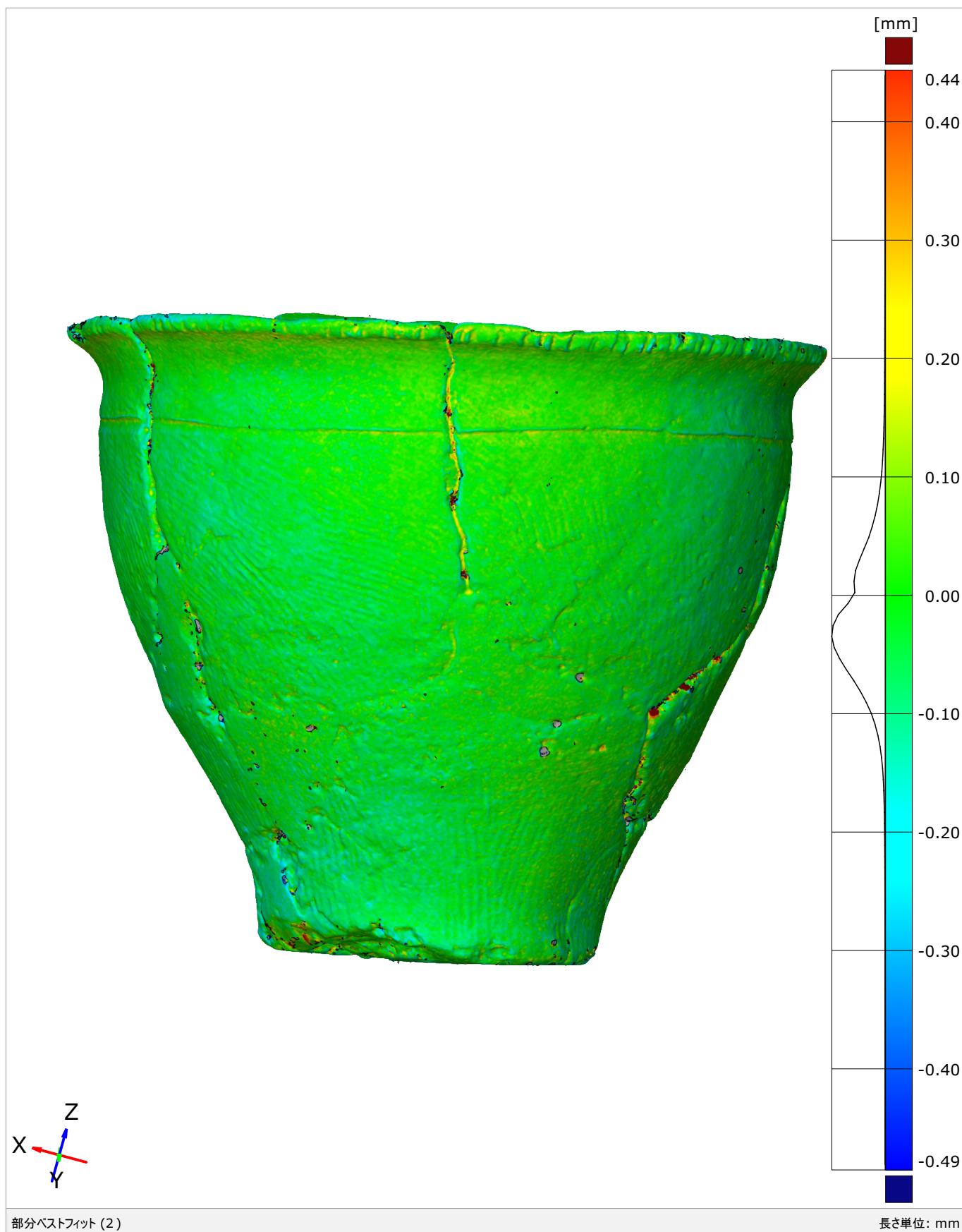

タイトルなし

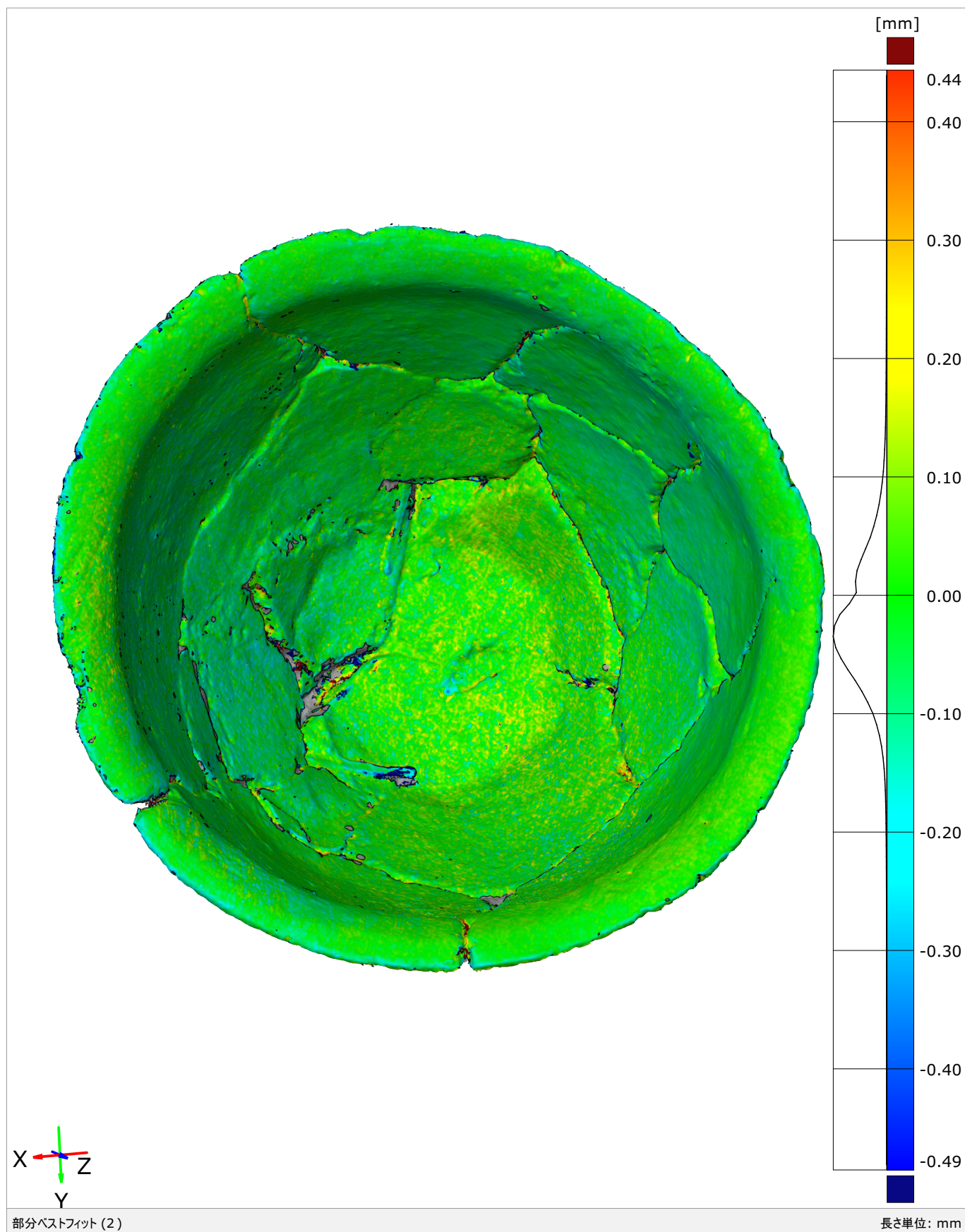

タイトルなし

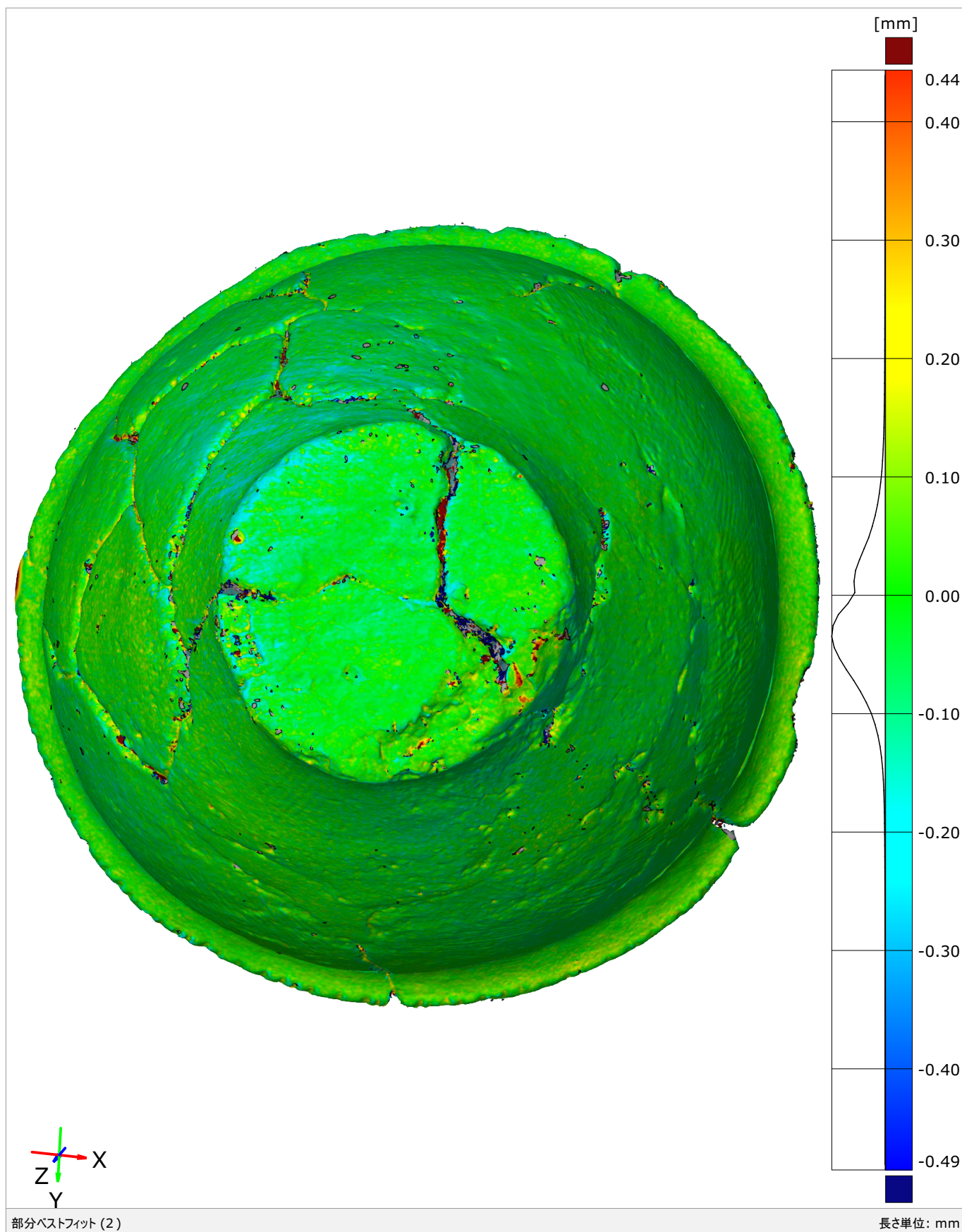

Supplement: S2 Fig — (PDF) [file pone.0270660.s002.pdf]

タイトルなし

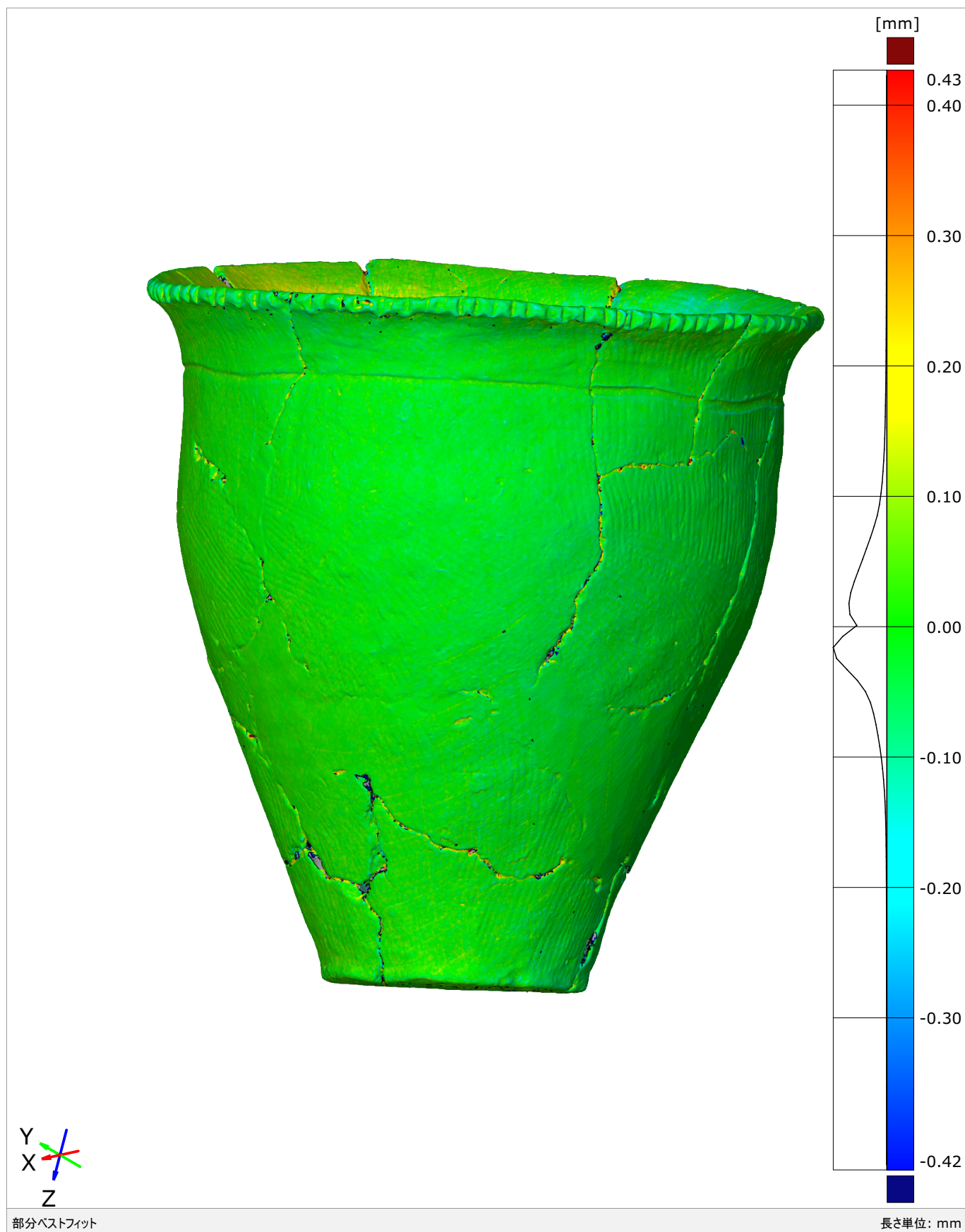

タイトルなし

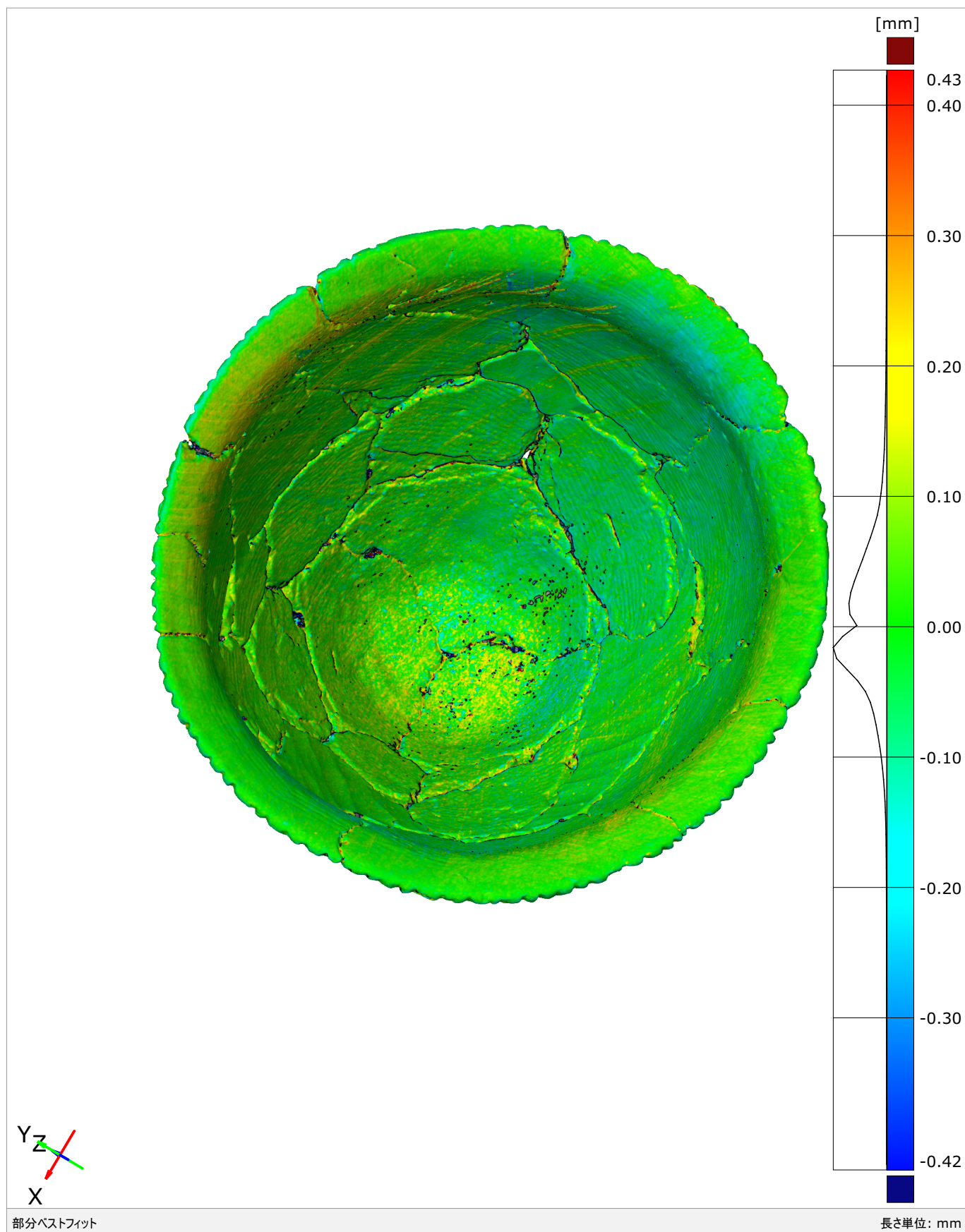

タイトルなし

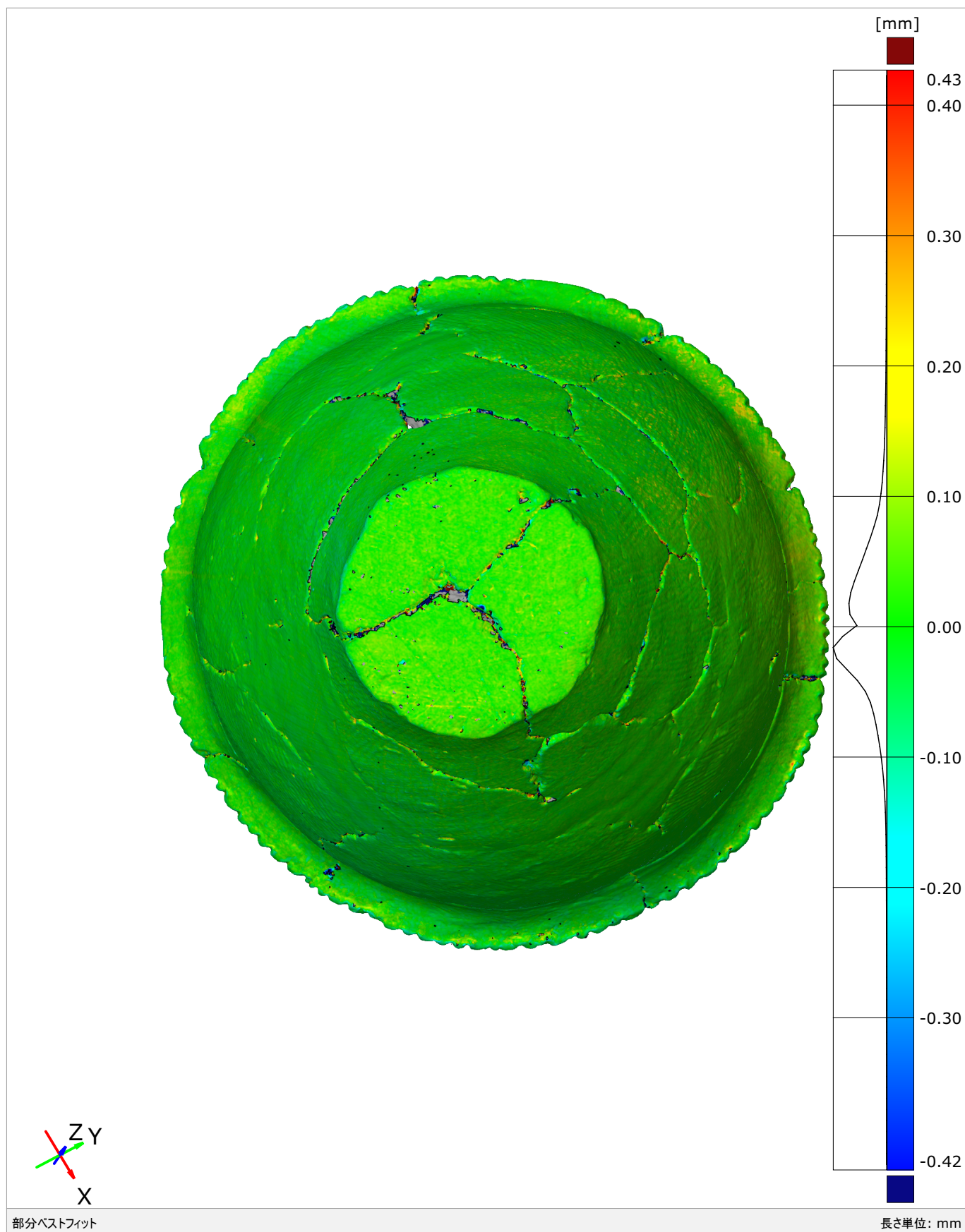

Supplement: S3 Fig — (PDF) [file pone.0270660.s003.pdf]

タイトルなし

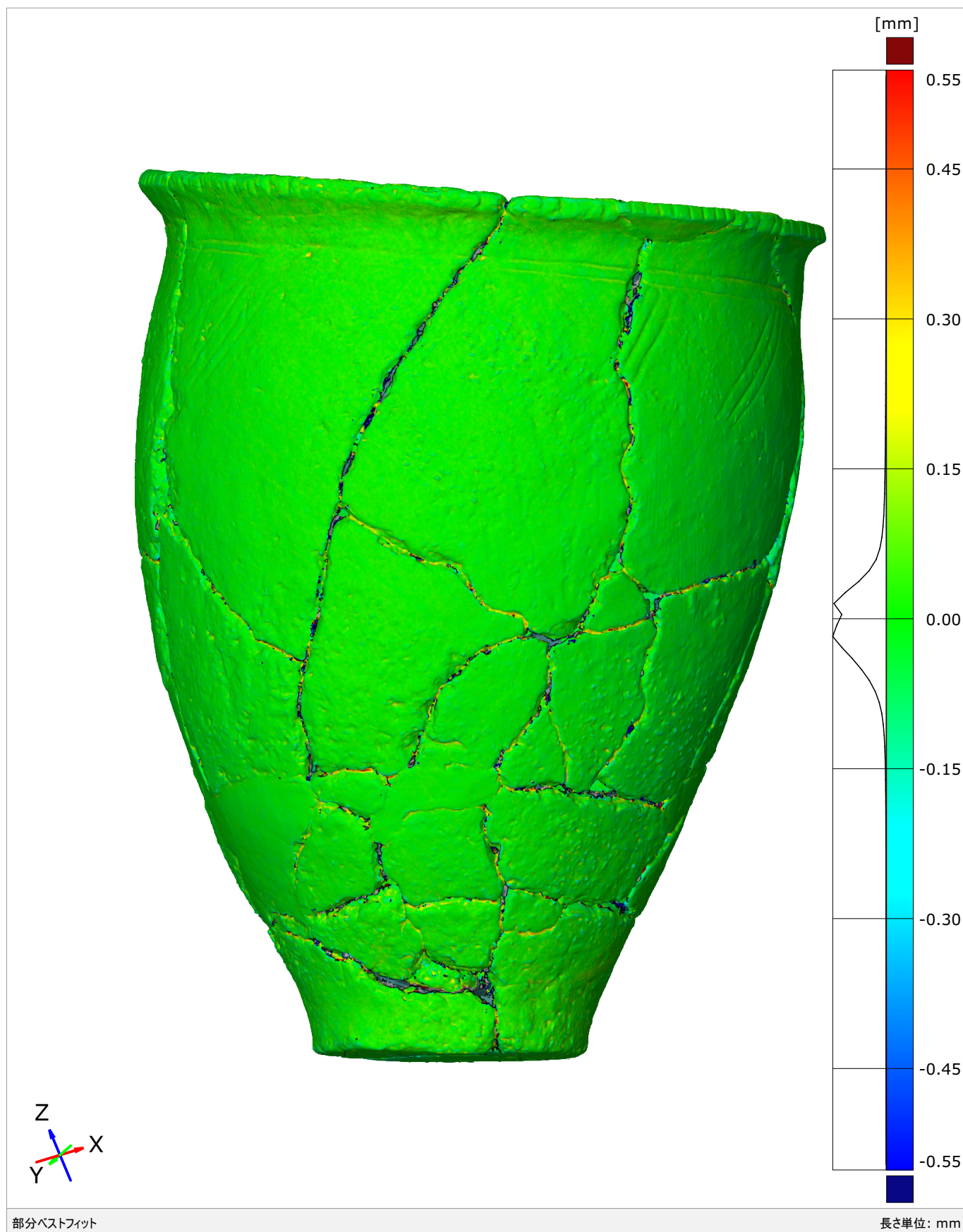

タイトルなし

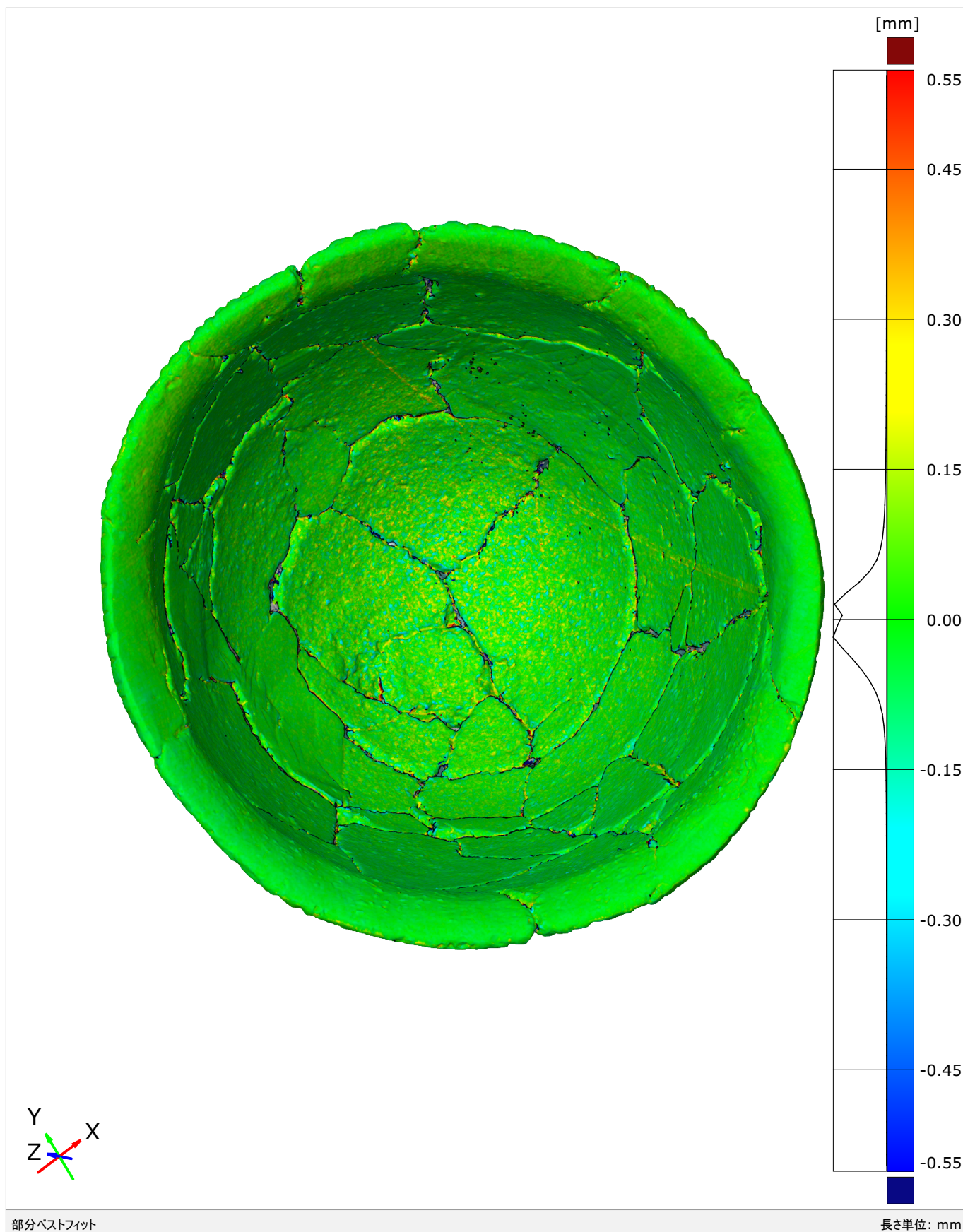

タイトルなし

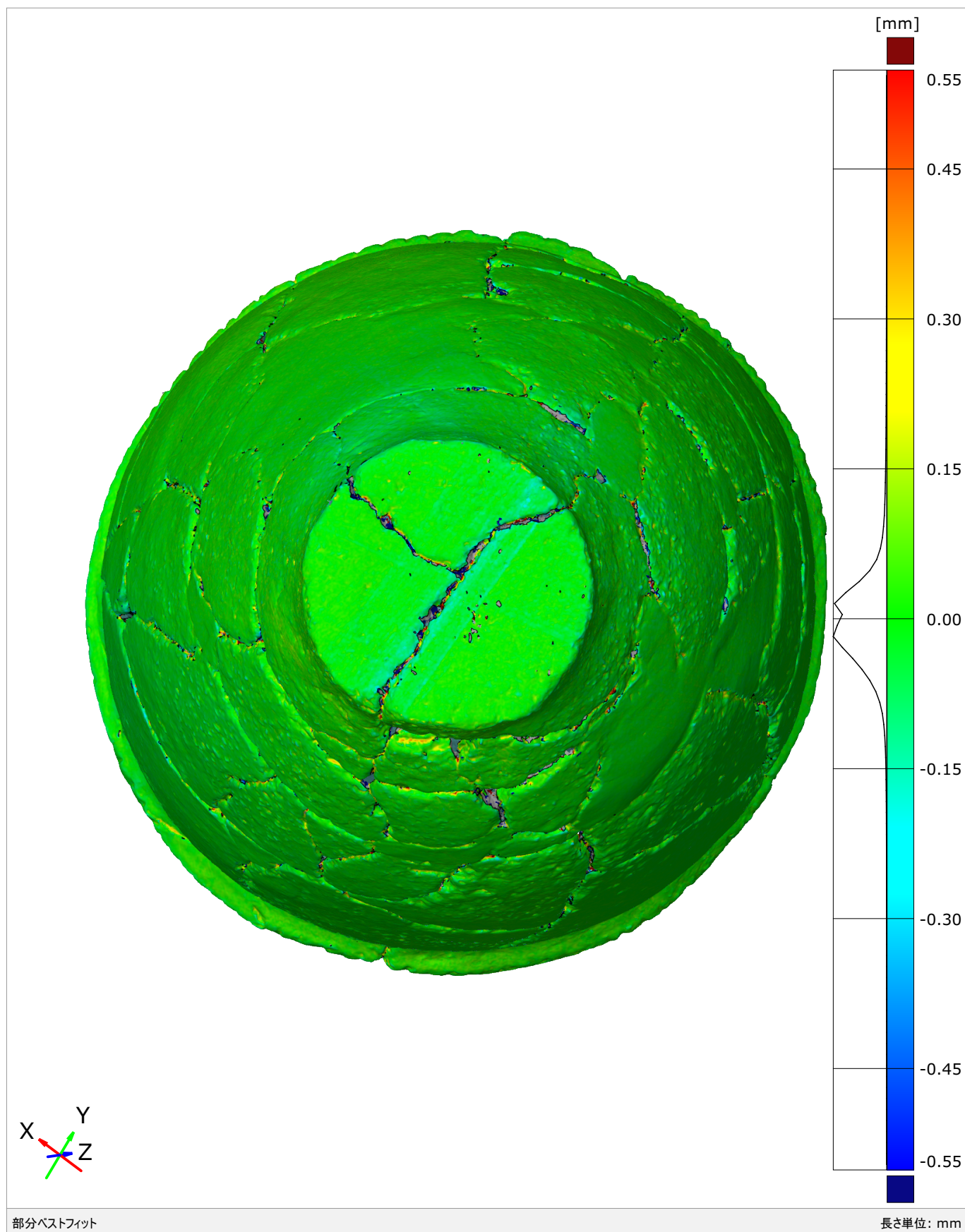

Supplement: S4 Fig — (PDF) [file pone.0270660.s004.pdf]

タイトルなし

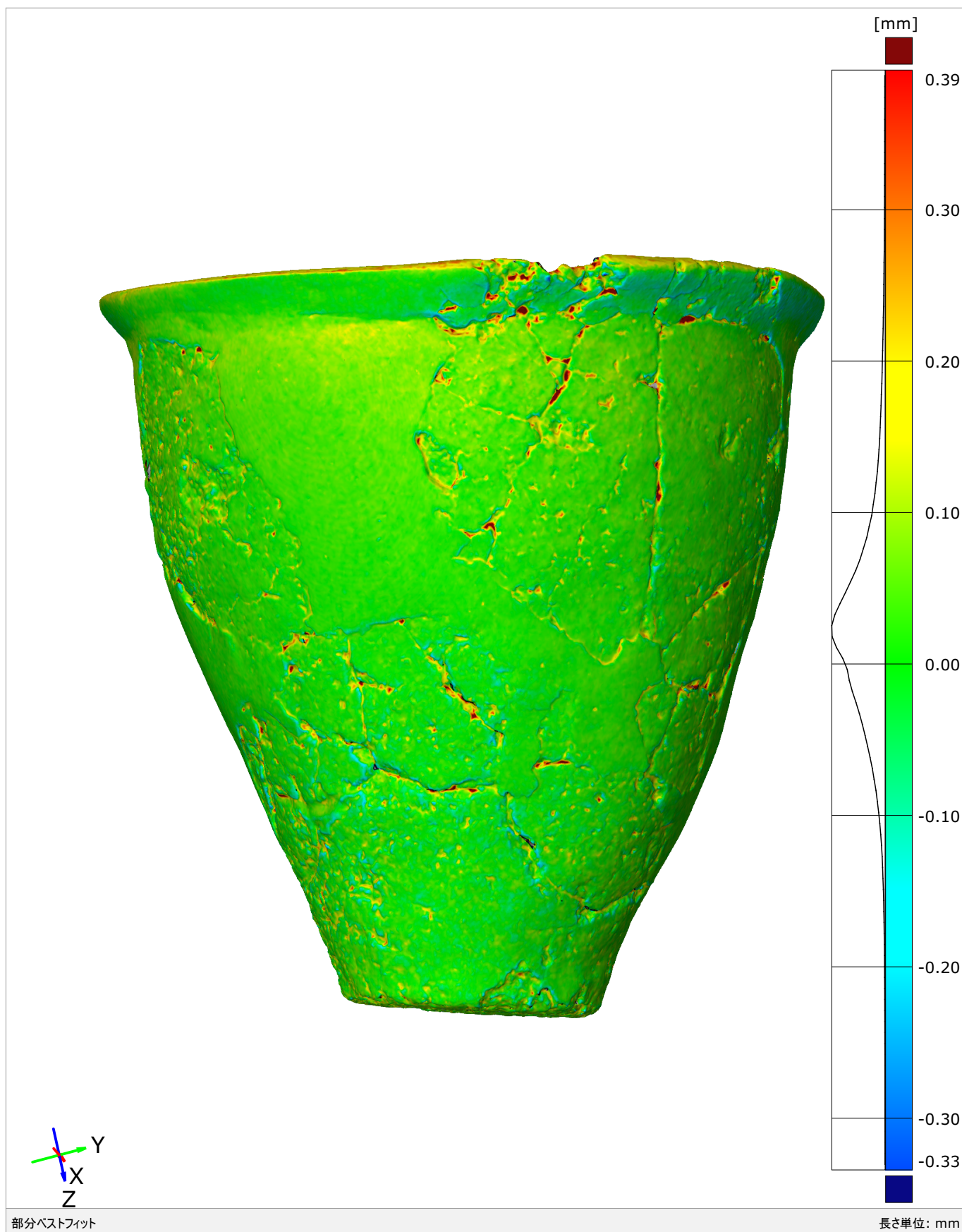

タイトルなし

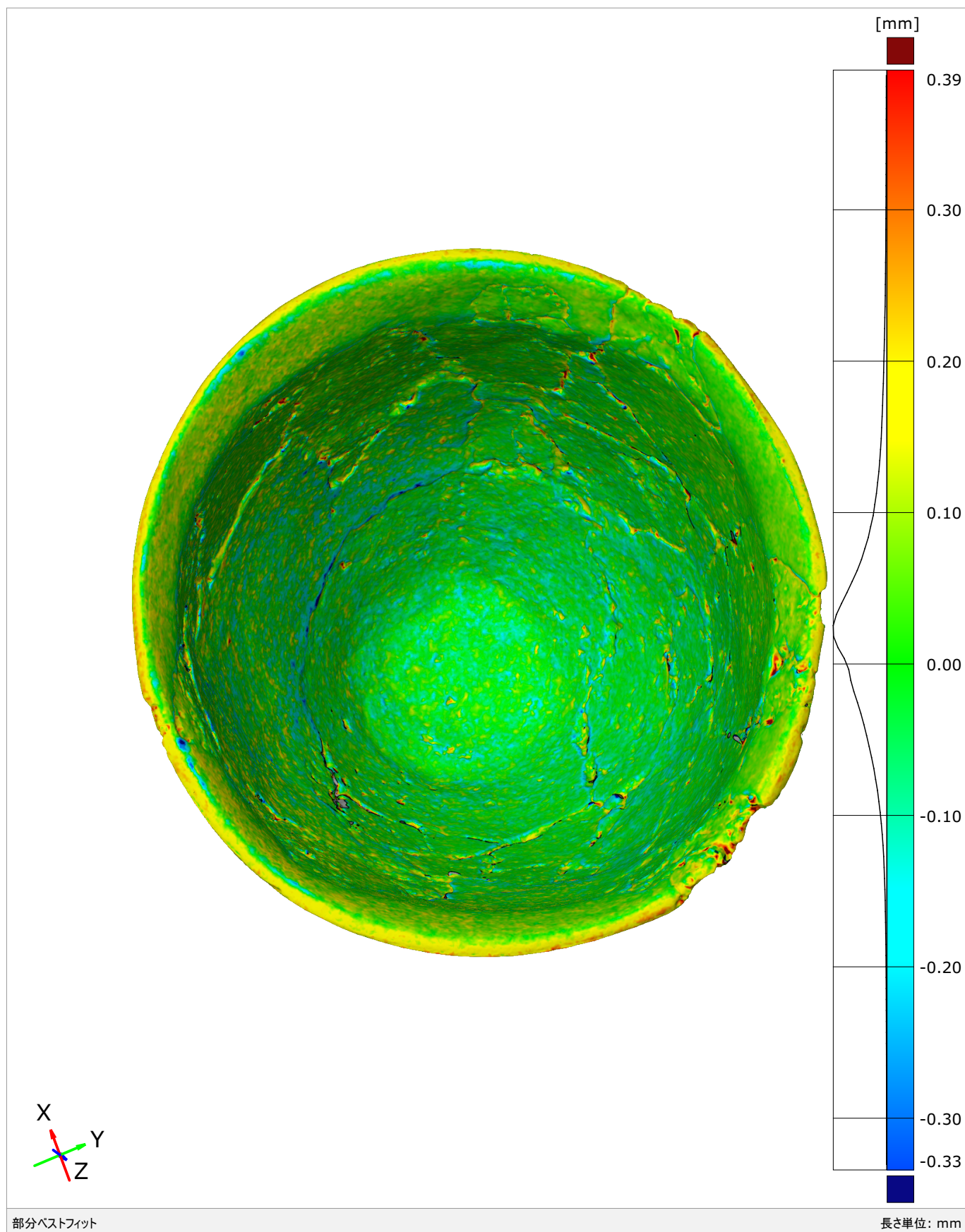

タイトルなし

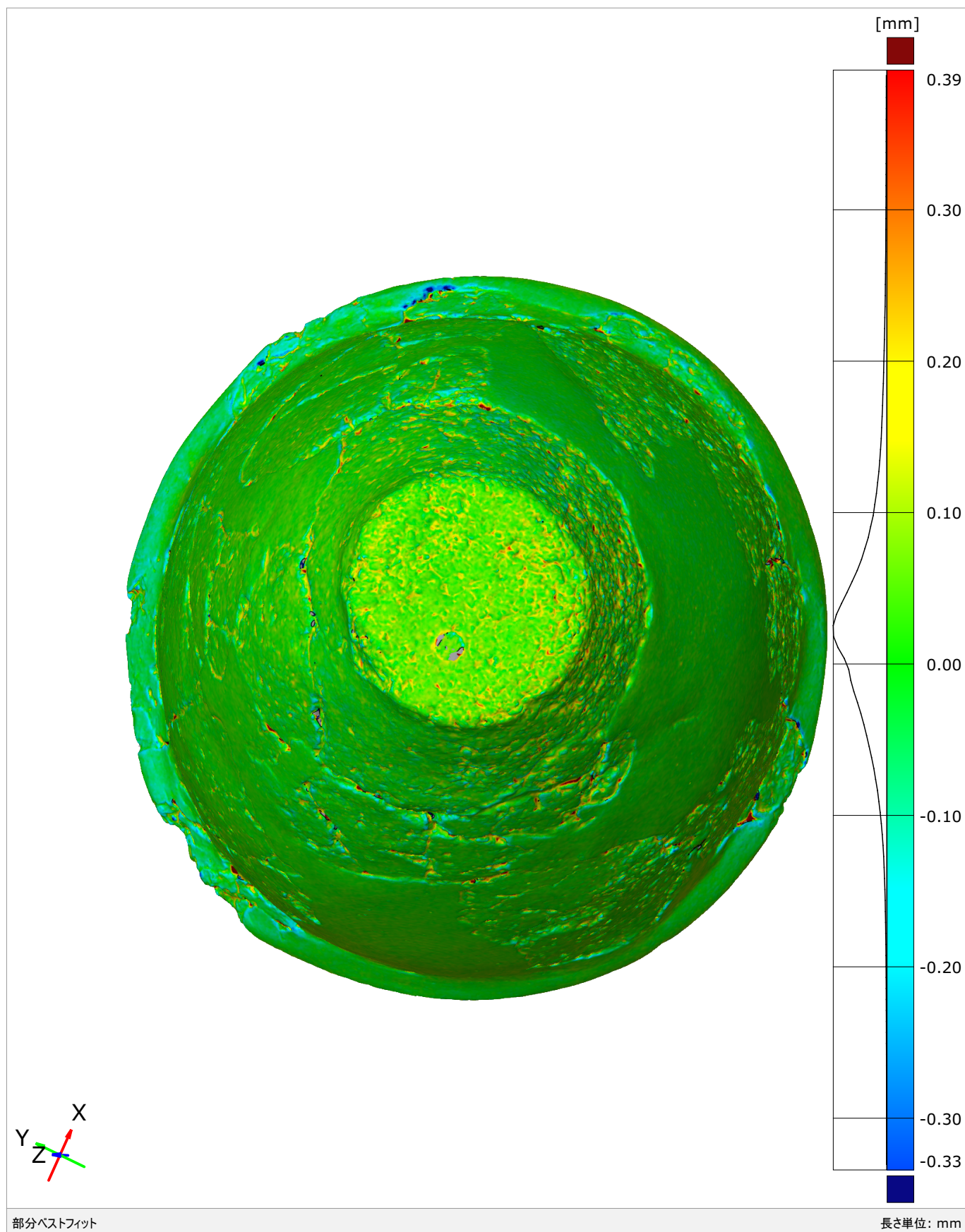

Supplement: S5 Fig — (PDF) [file pone.0270660.s005.pdf]

タイトルなし

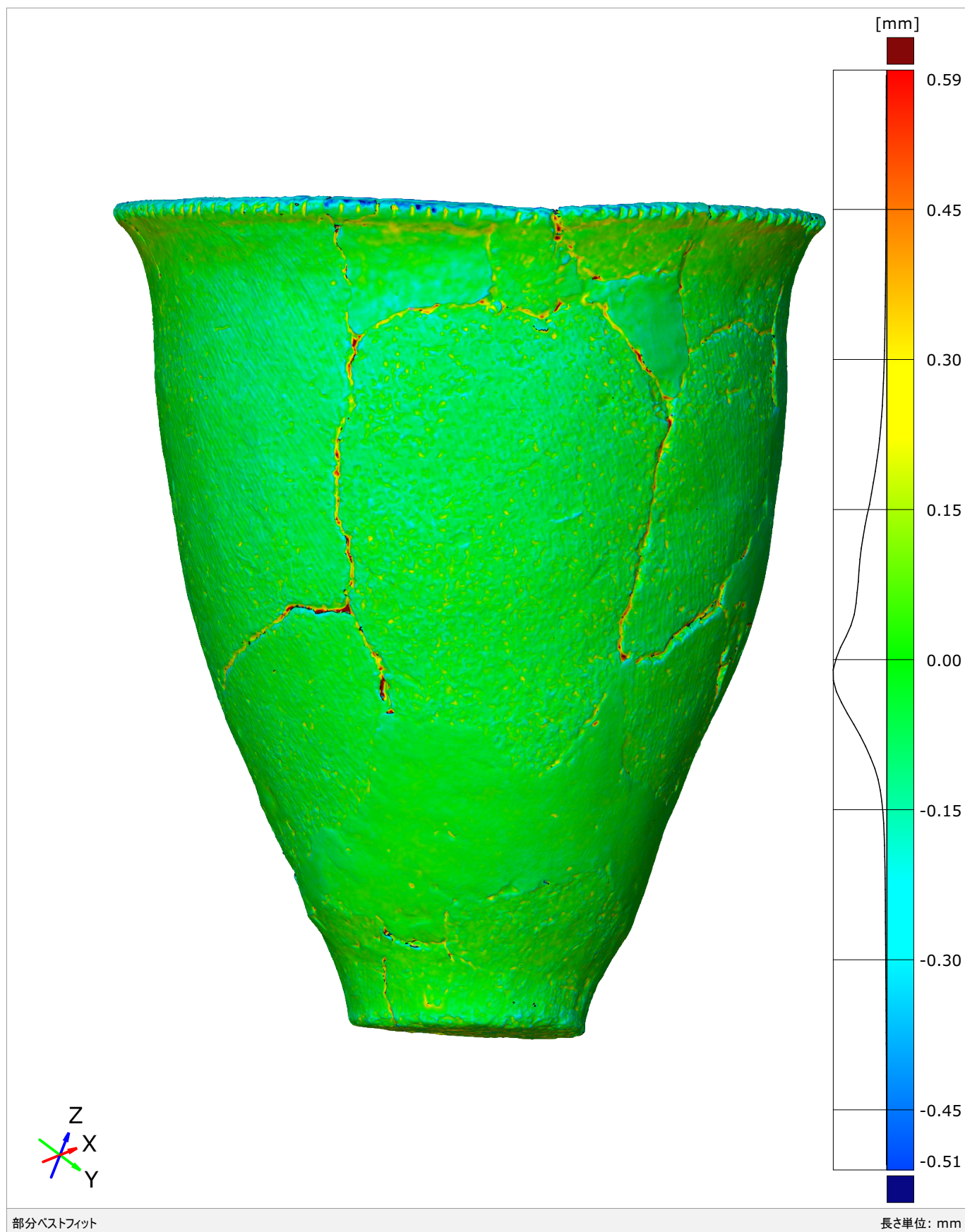

タイトルなし

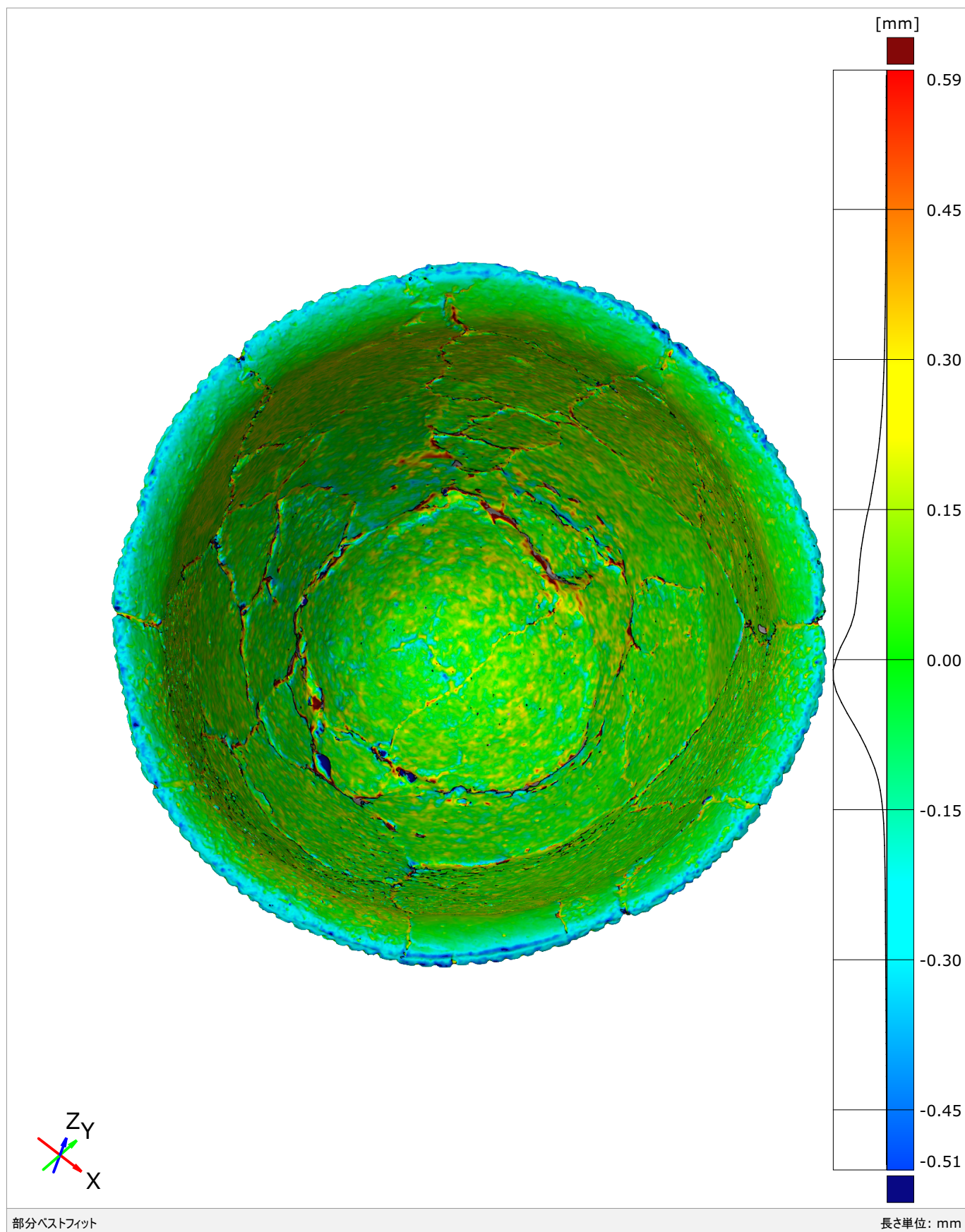

タイトルなし

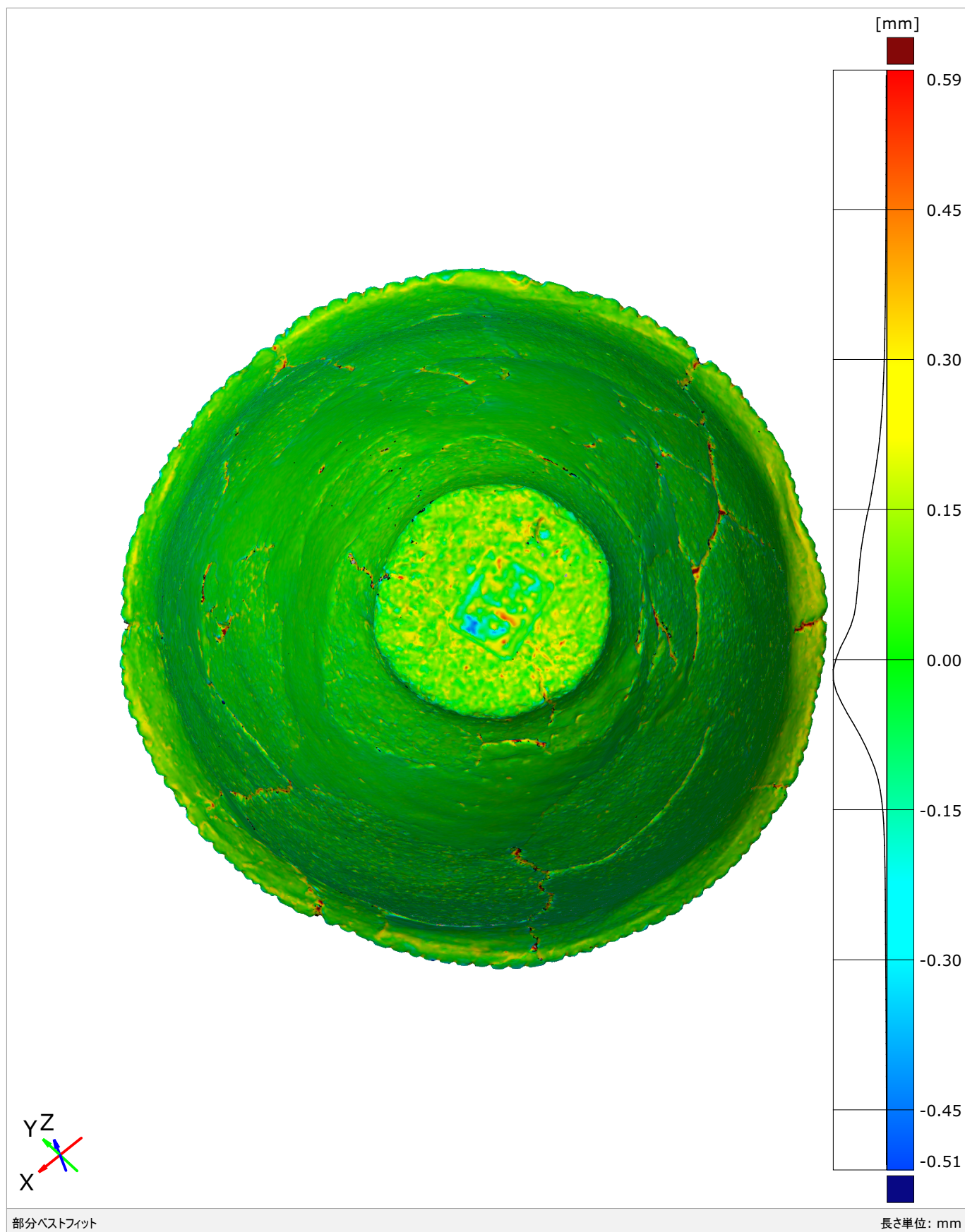

Supplement: S6 Fig — (PDF) [file pone.0270660.s006.pdf]

タイトルなし

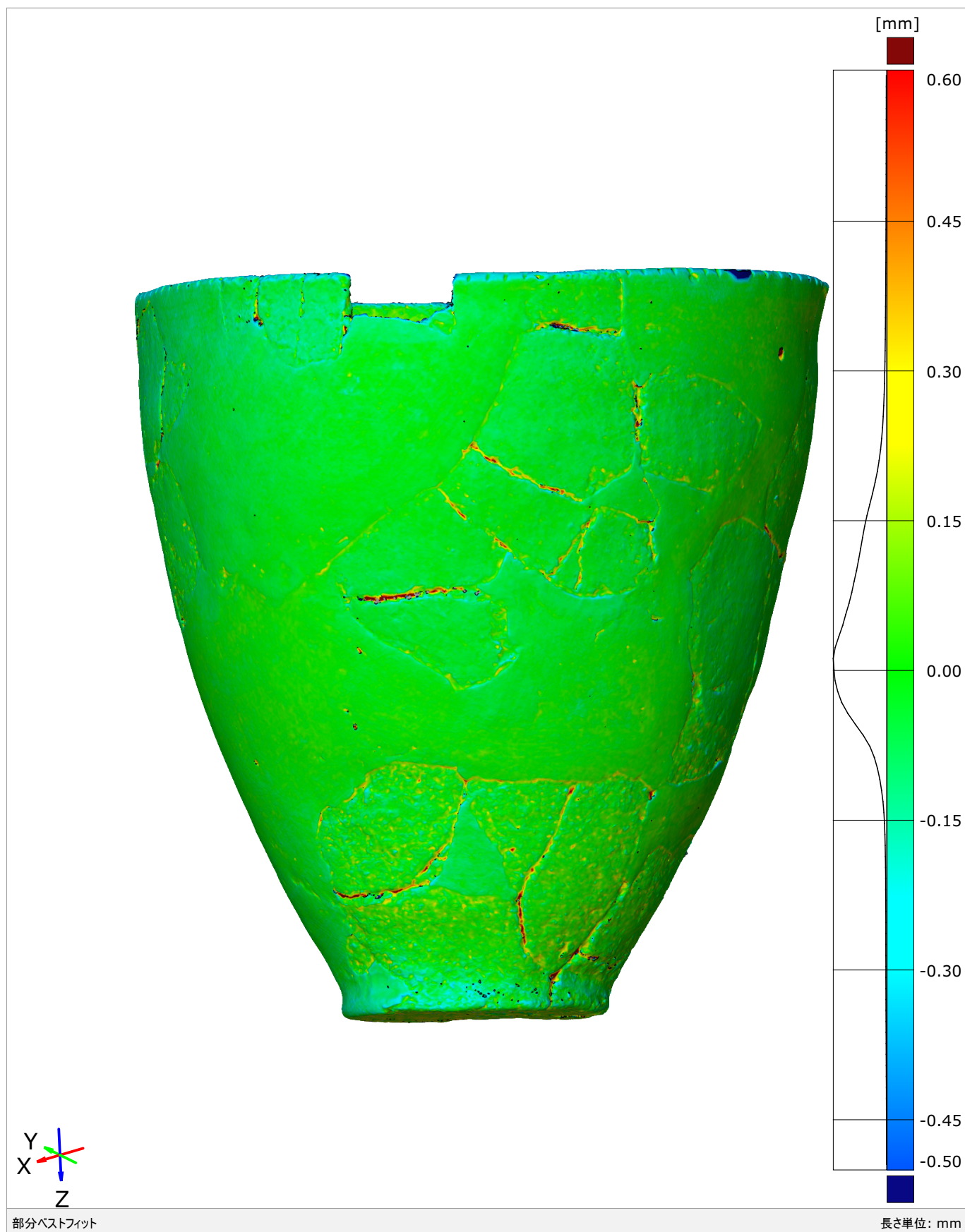

タイトルなし

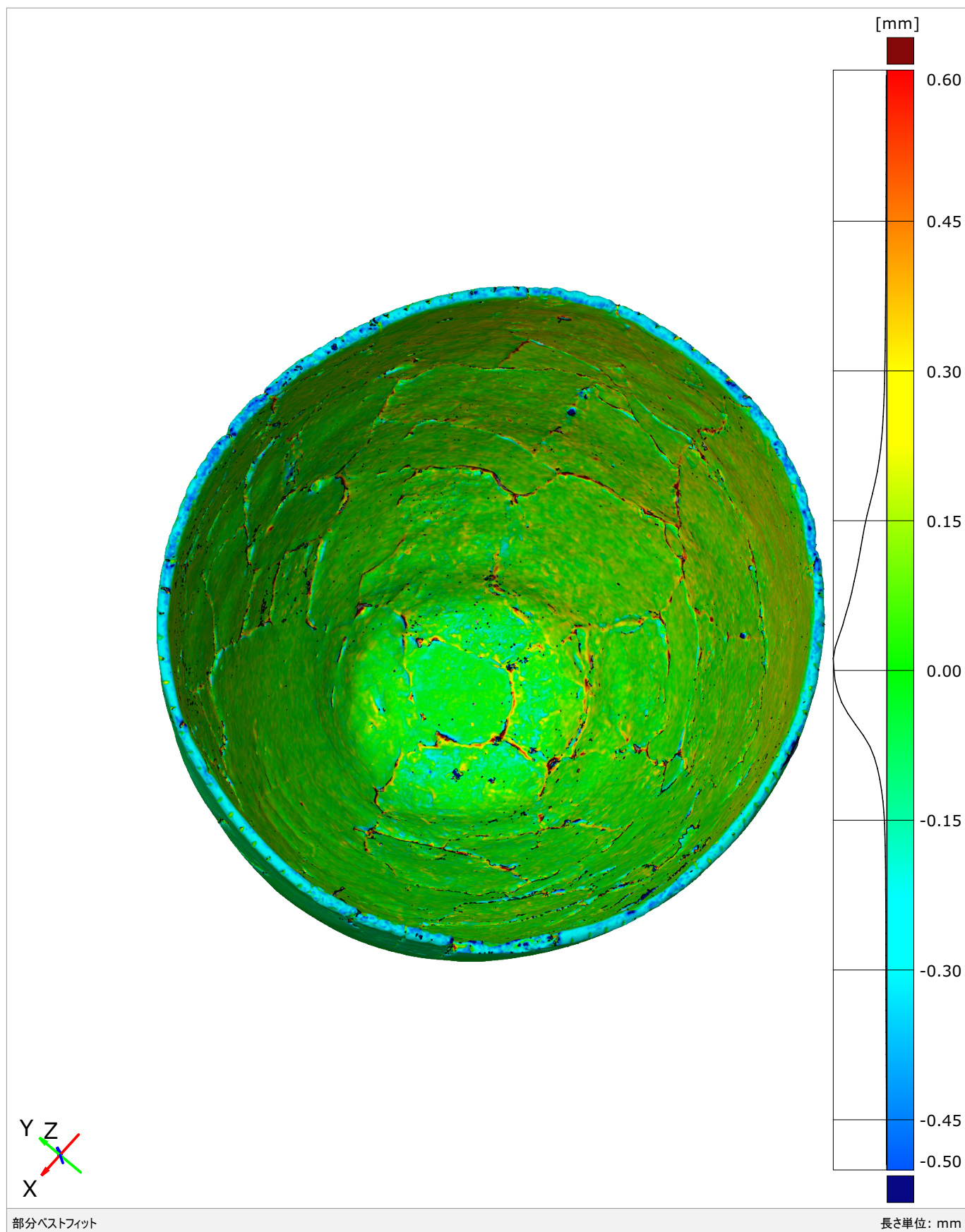

タイトルなし

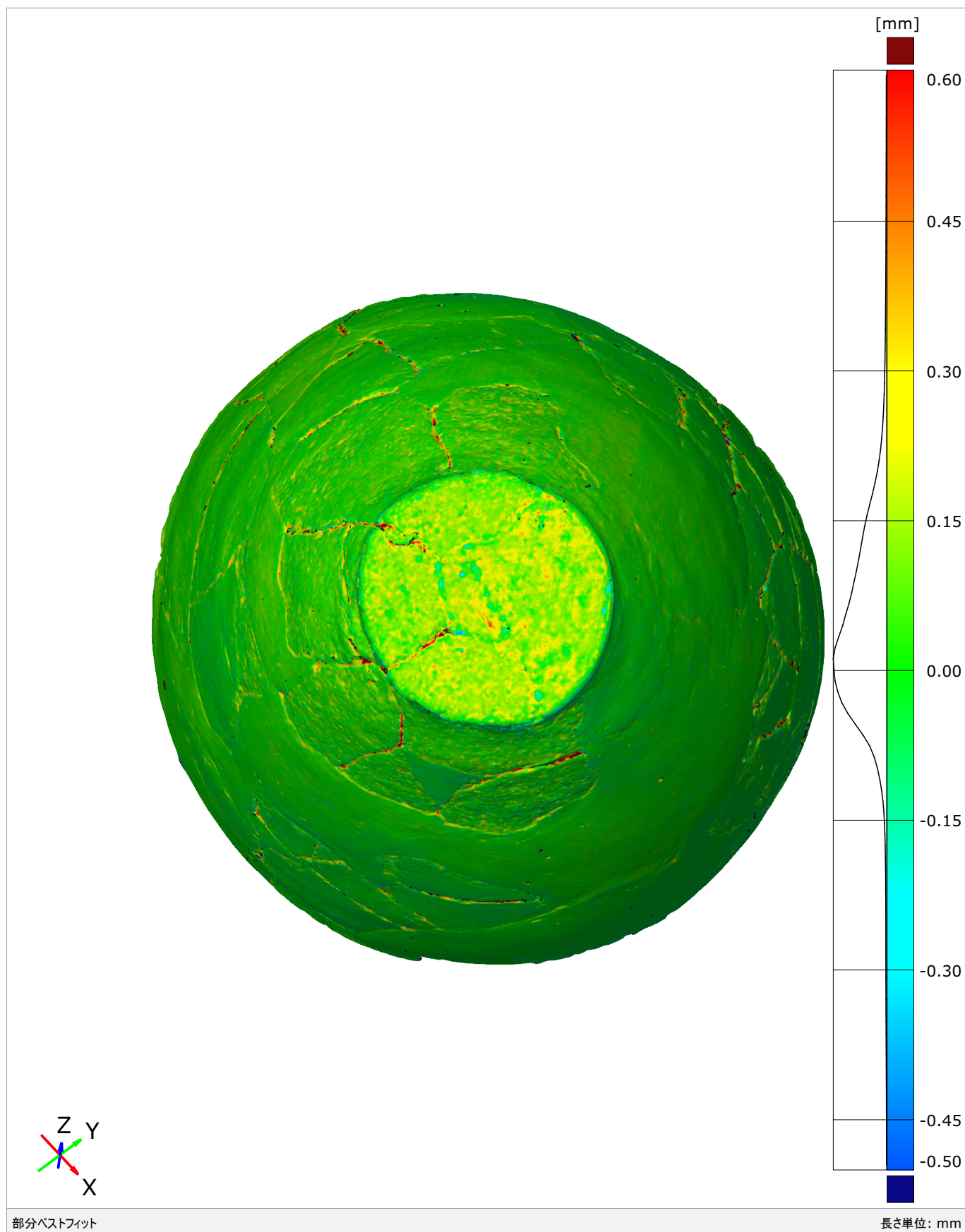

Supplement: S7 Fig — (PDF) [file pone.0270660.s007.pdf]

タイトルなし

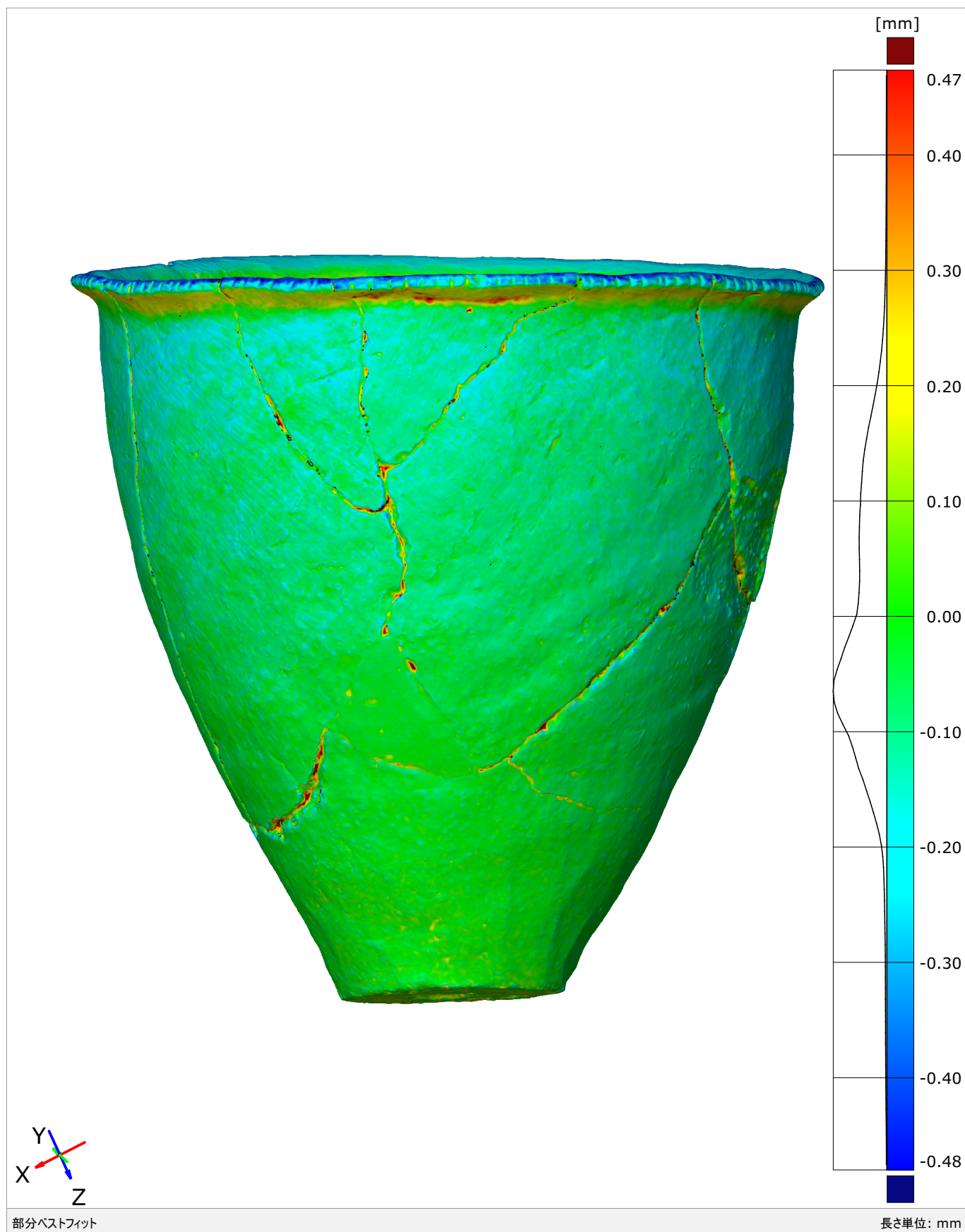

タイトルなし

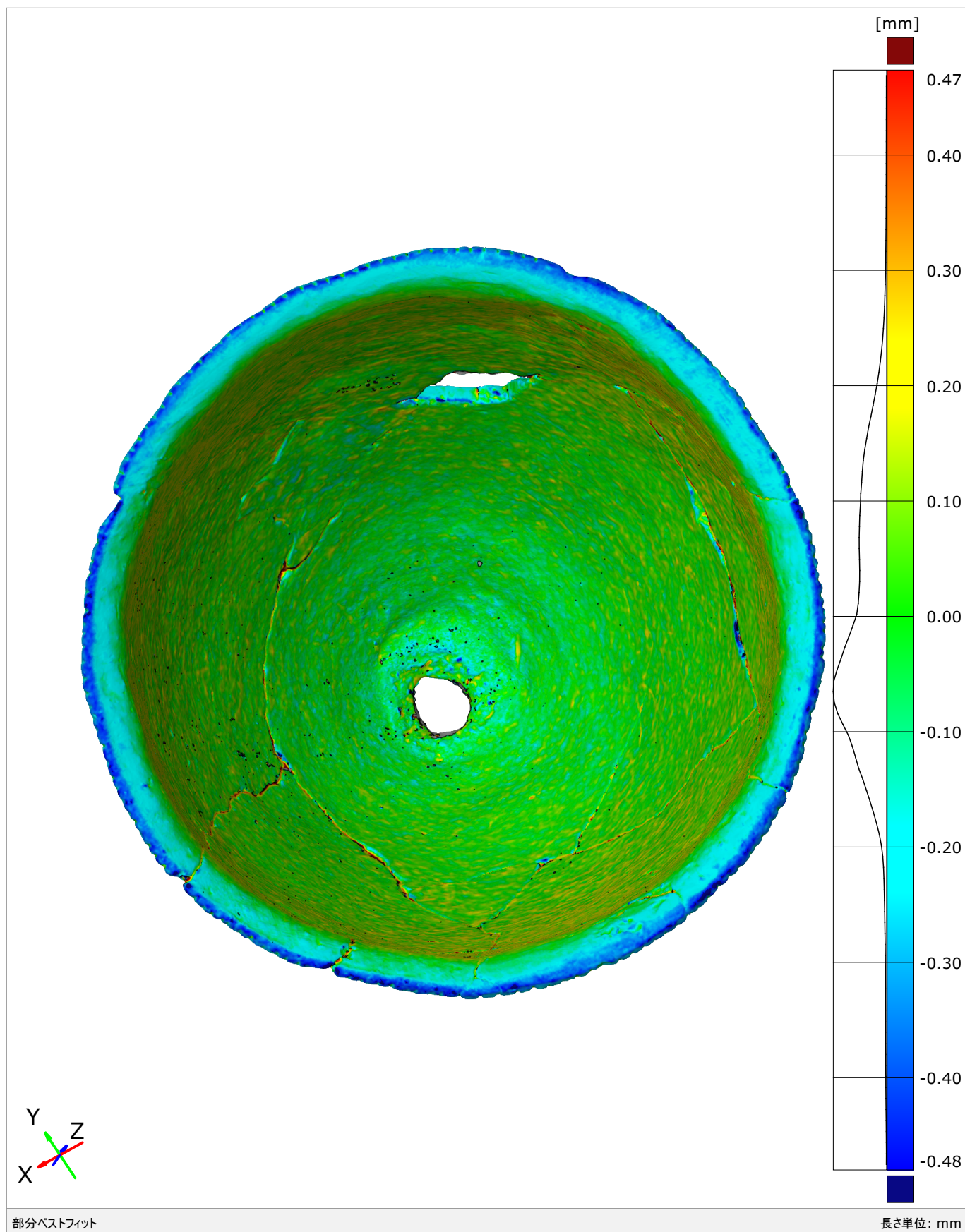

タイトルなし

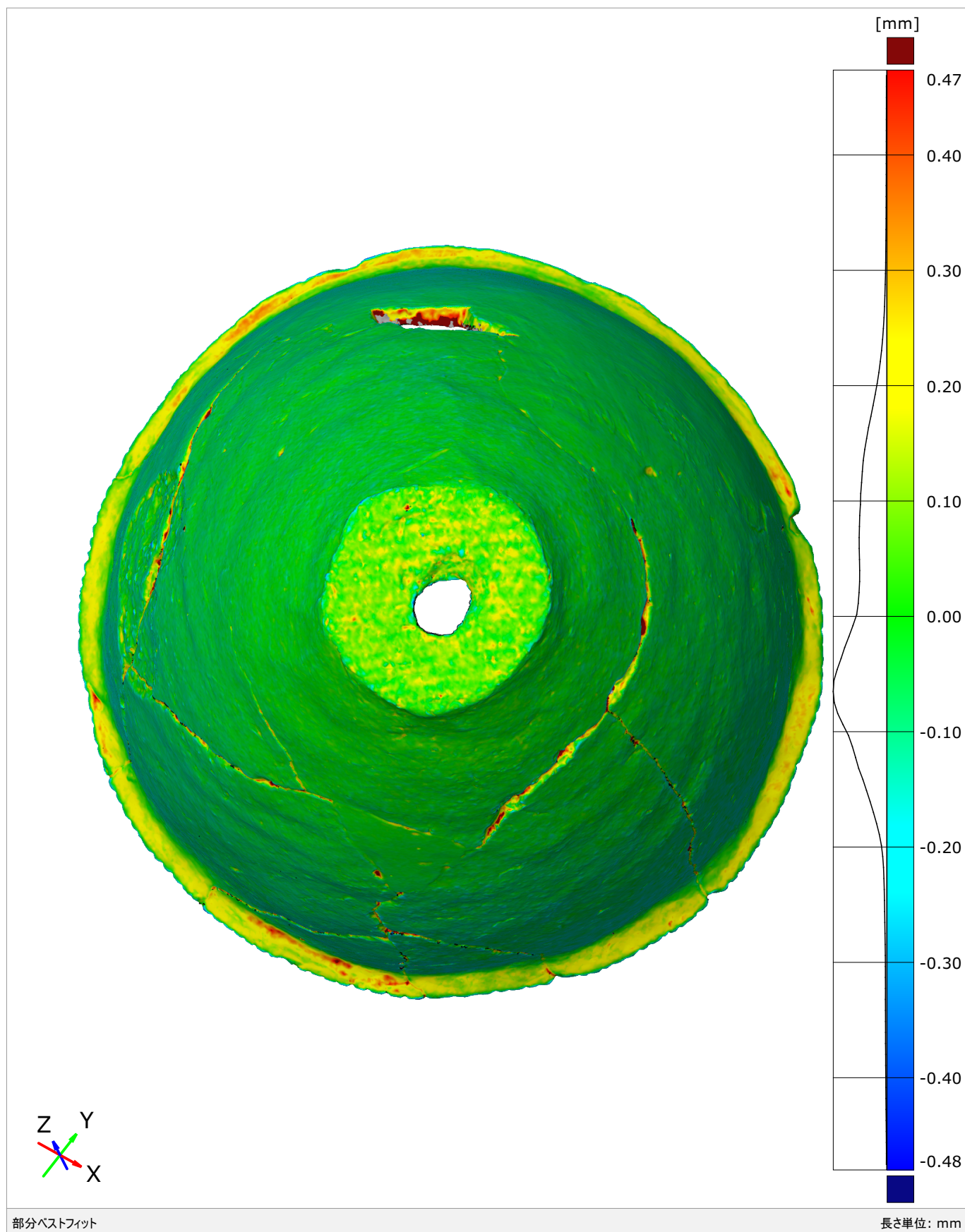

Supplement: S8 Fig — (PDF) [file pone.0270660.s008.pdf]

タイトルなし

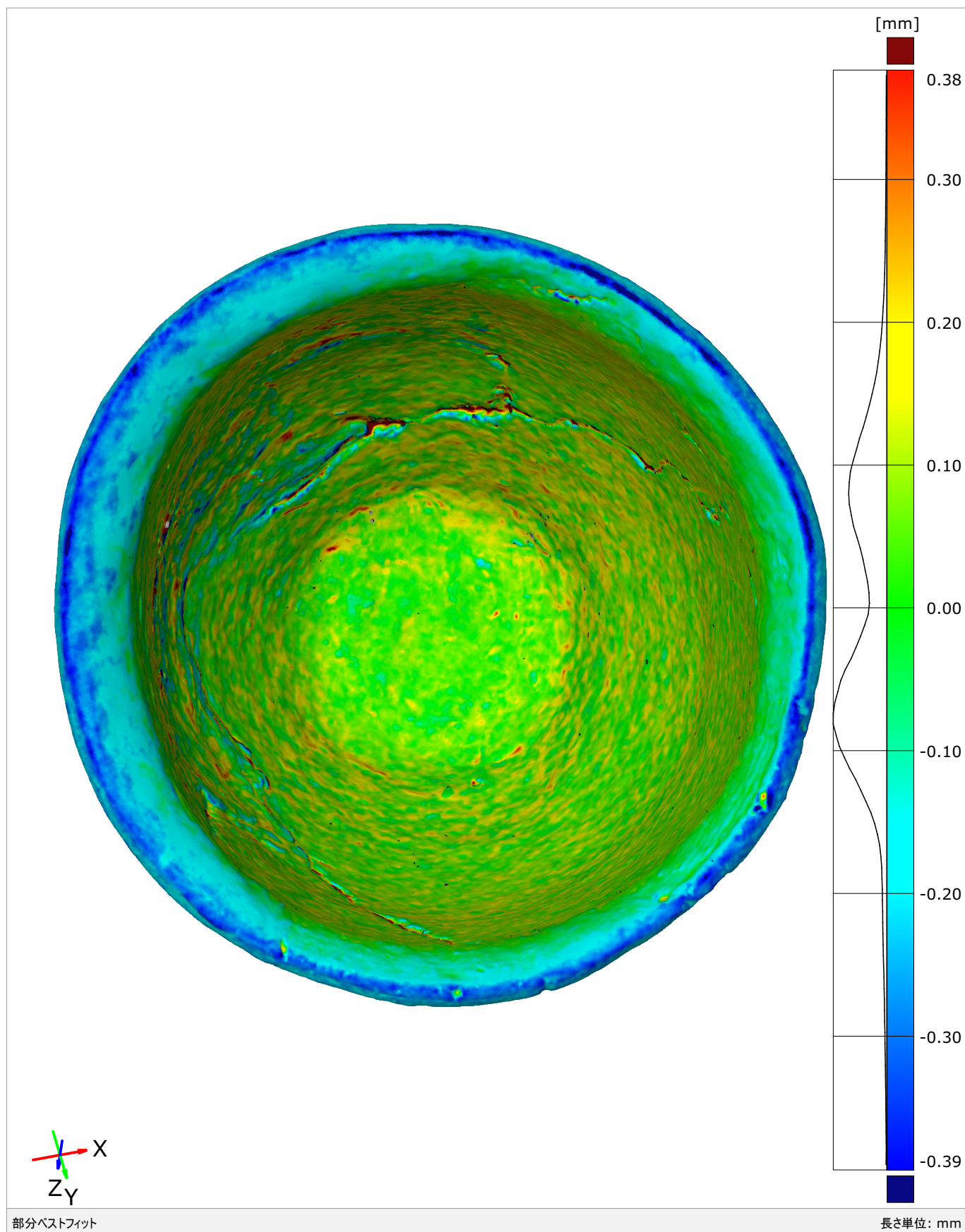

タイトルなし

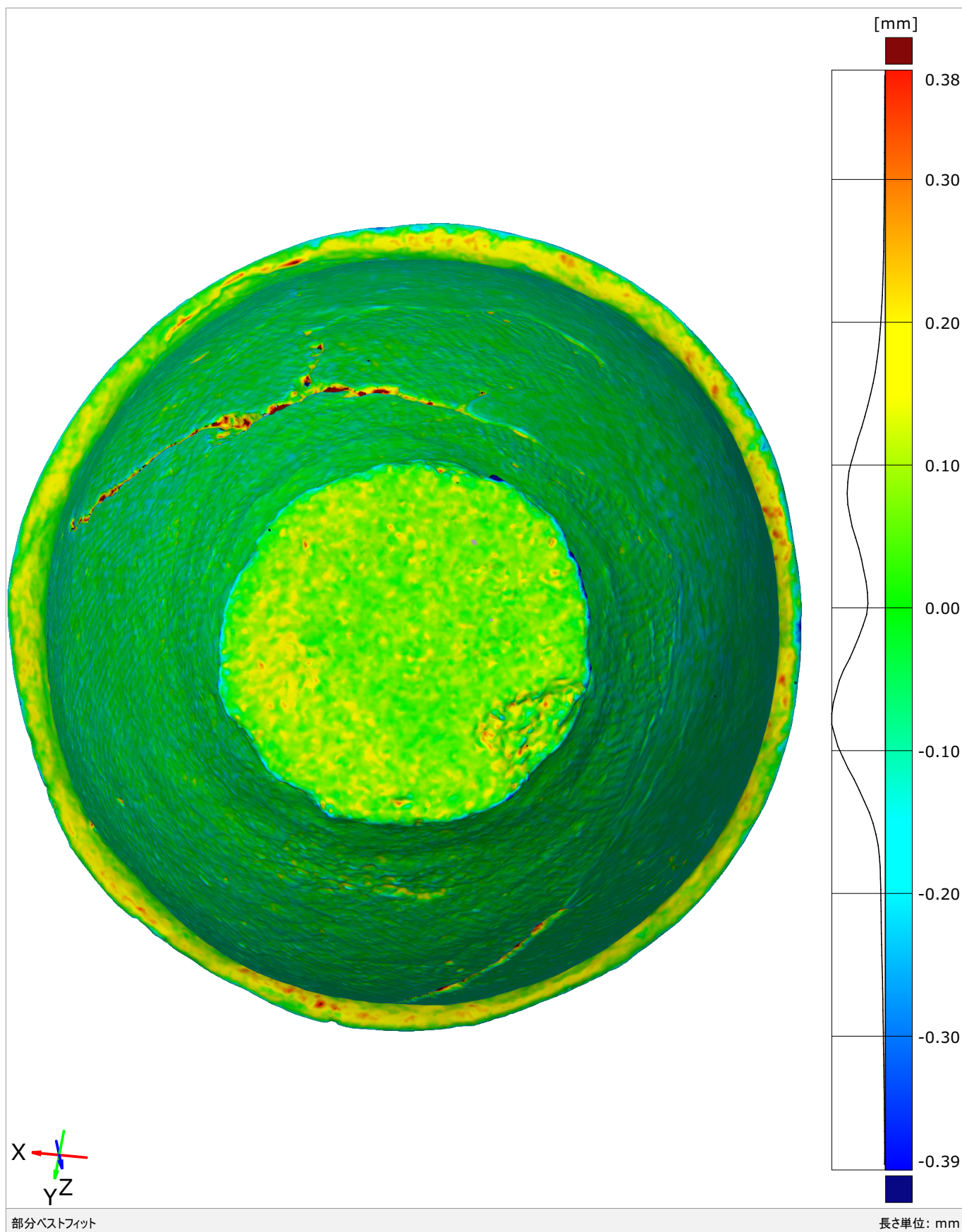

タイトルなし

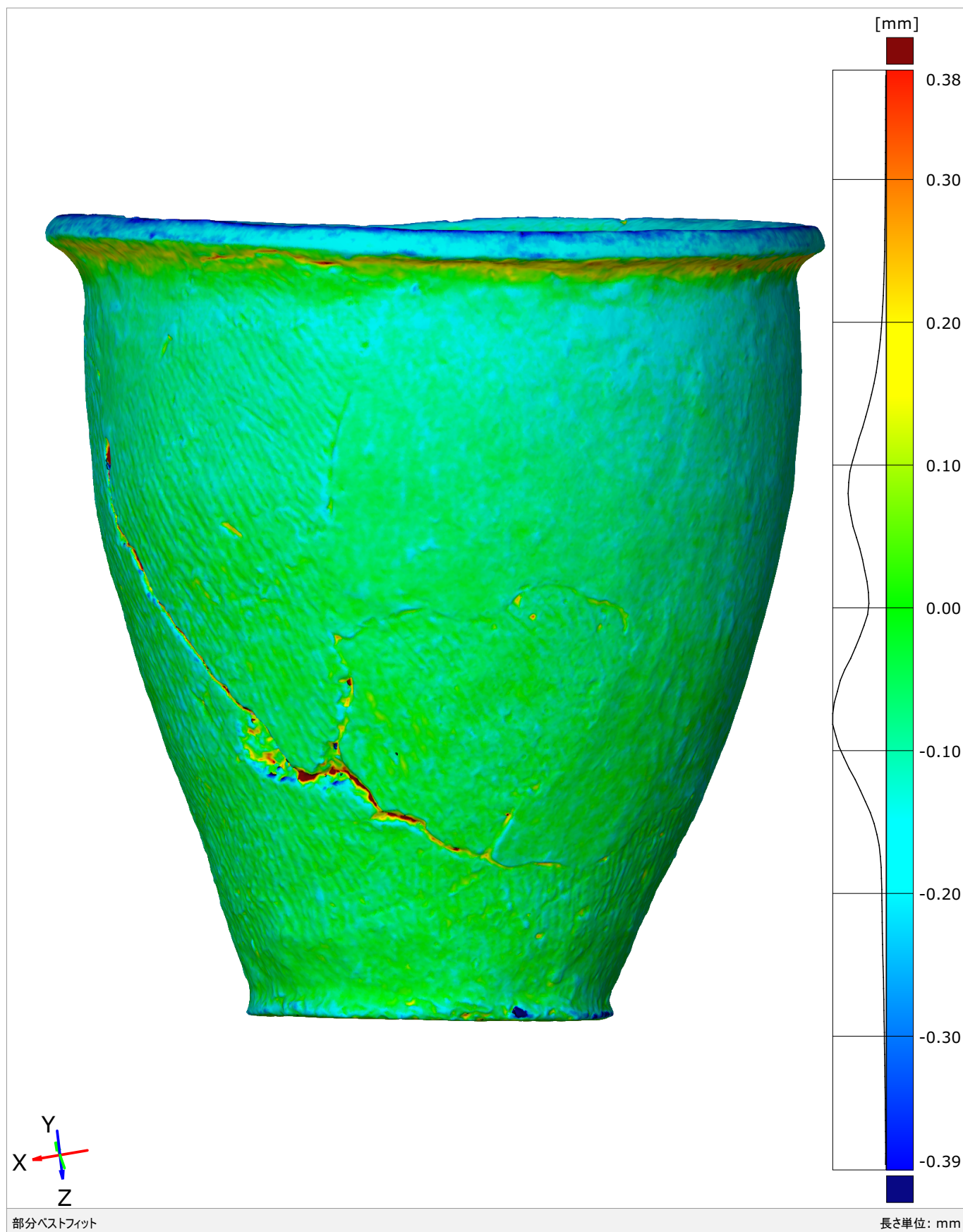

Supplement: S9 Fig — (PDF) [file pone.0270660.s009.pdf]

タイトルなし

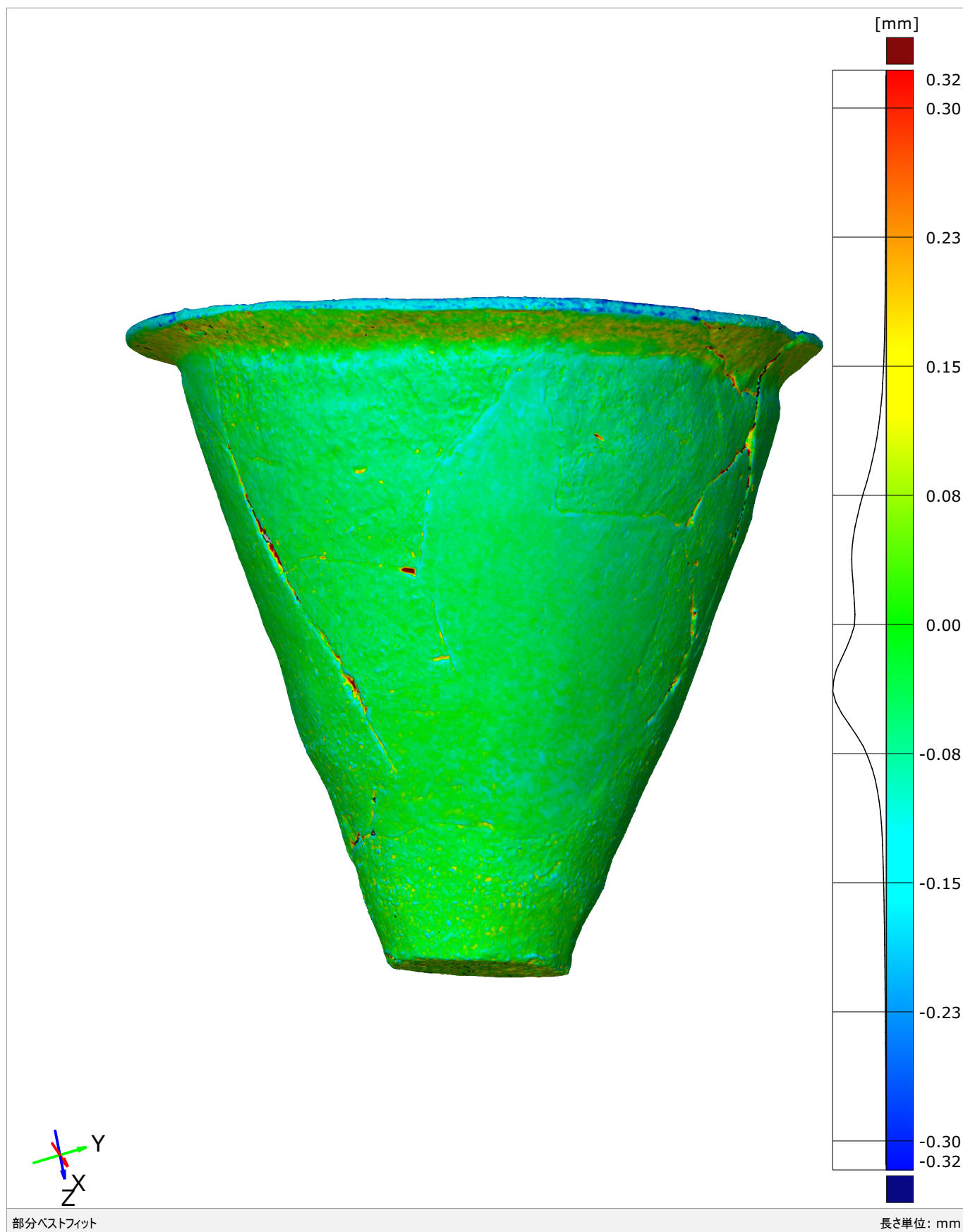

タイトルなし

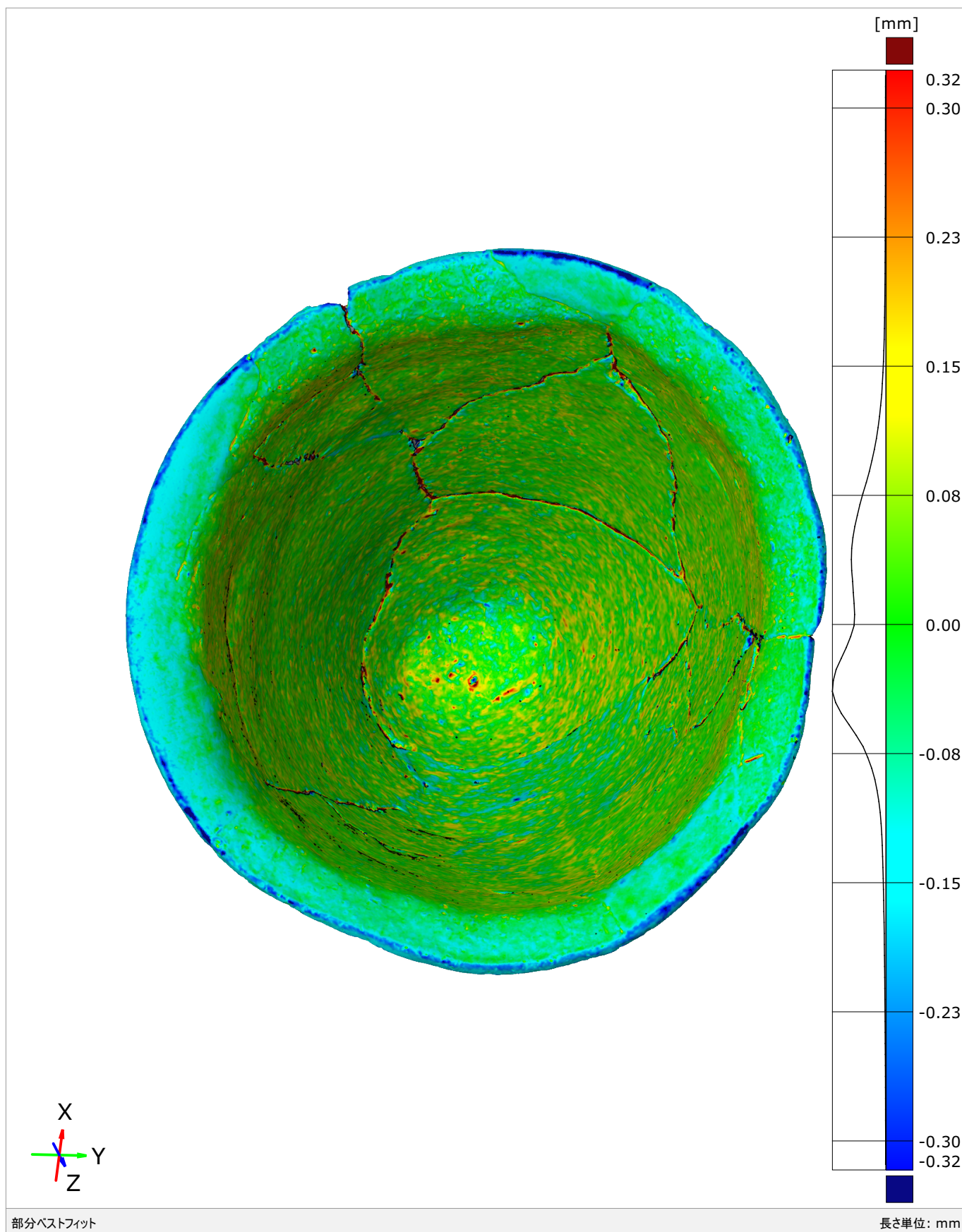

タイトルなし

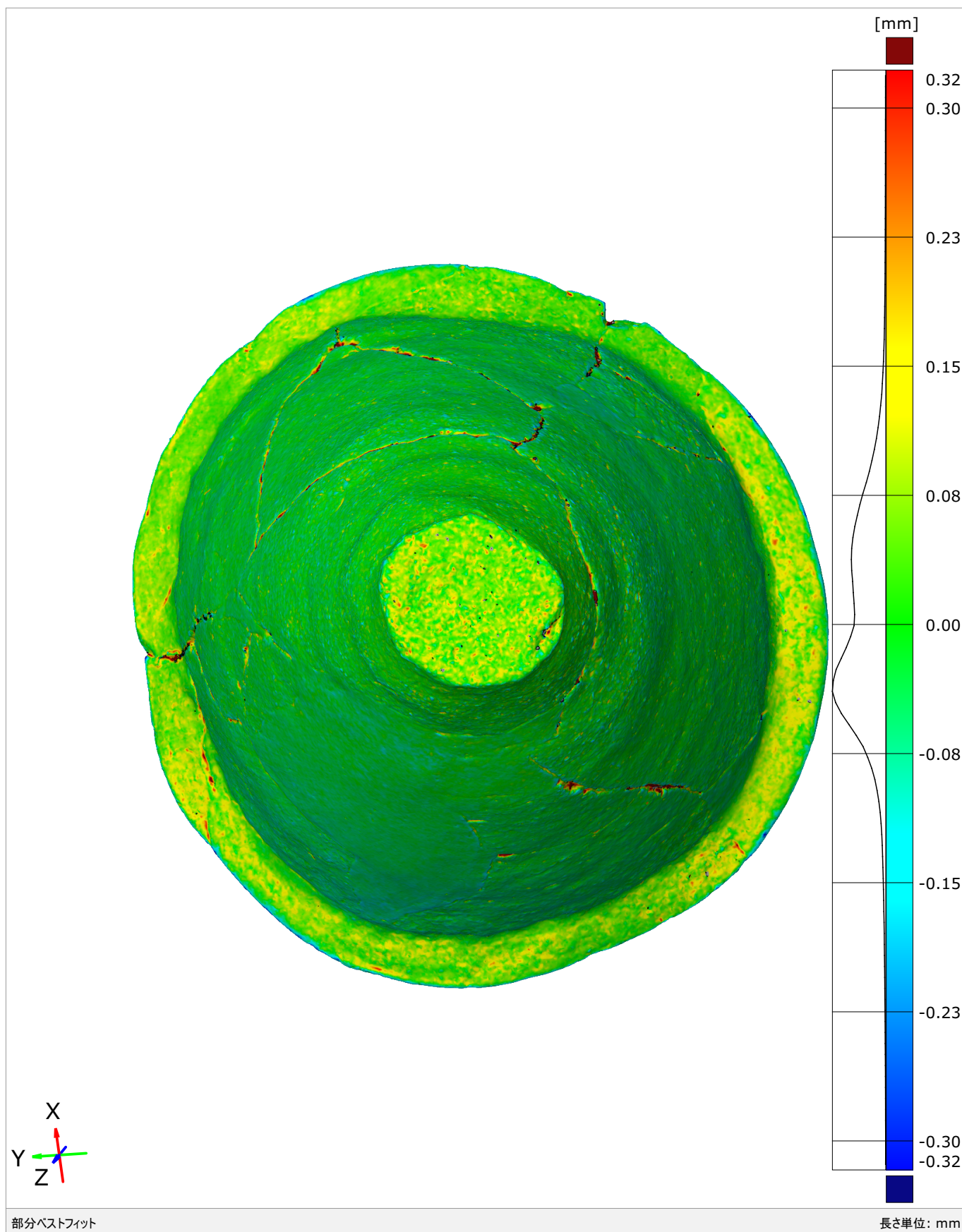

Supplement: S10 Fig — (PDF) [file pone.0270660.s010.pdf]

タイトルなし

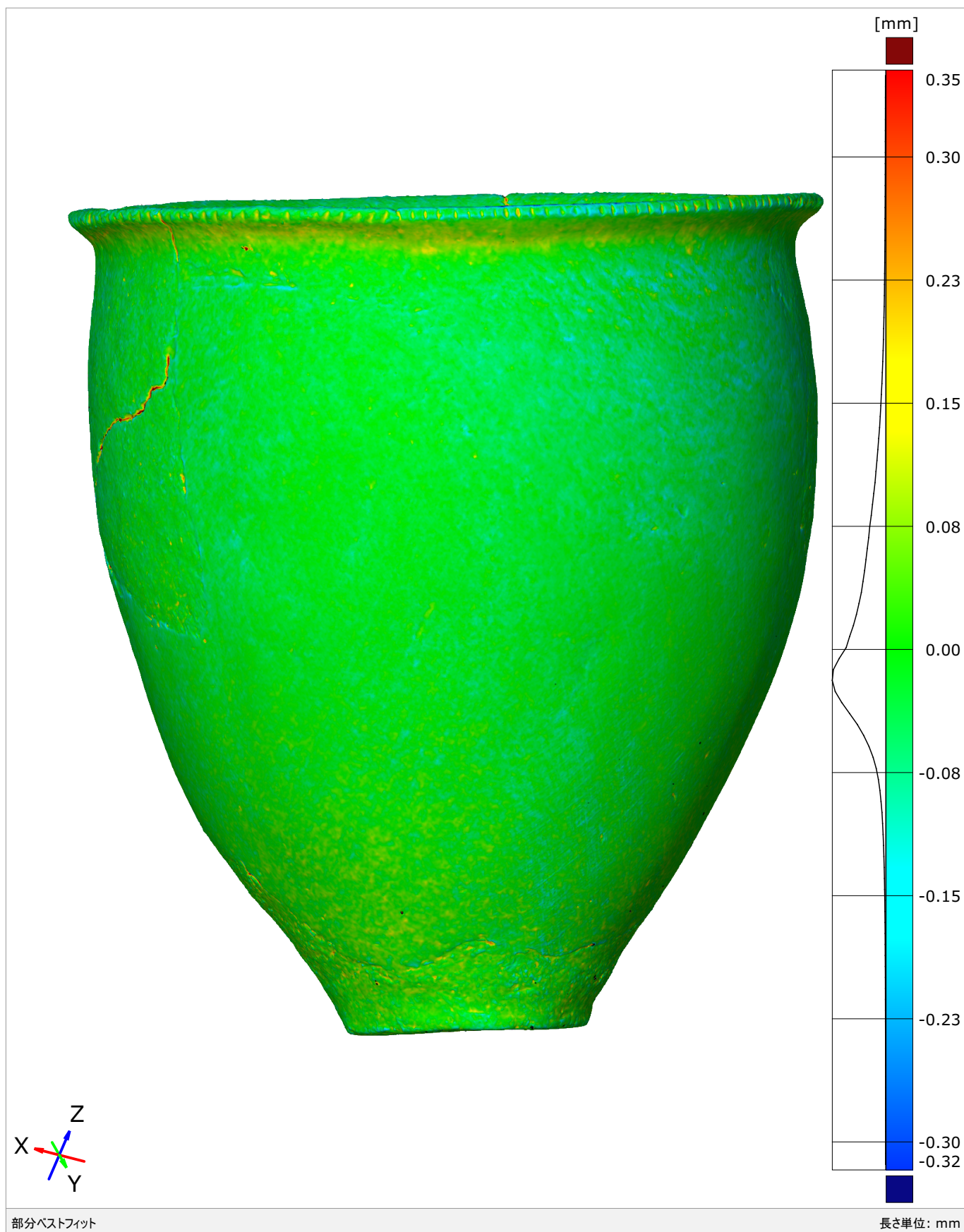

タイトルなし

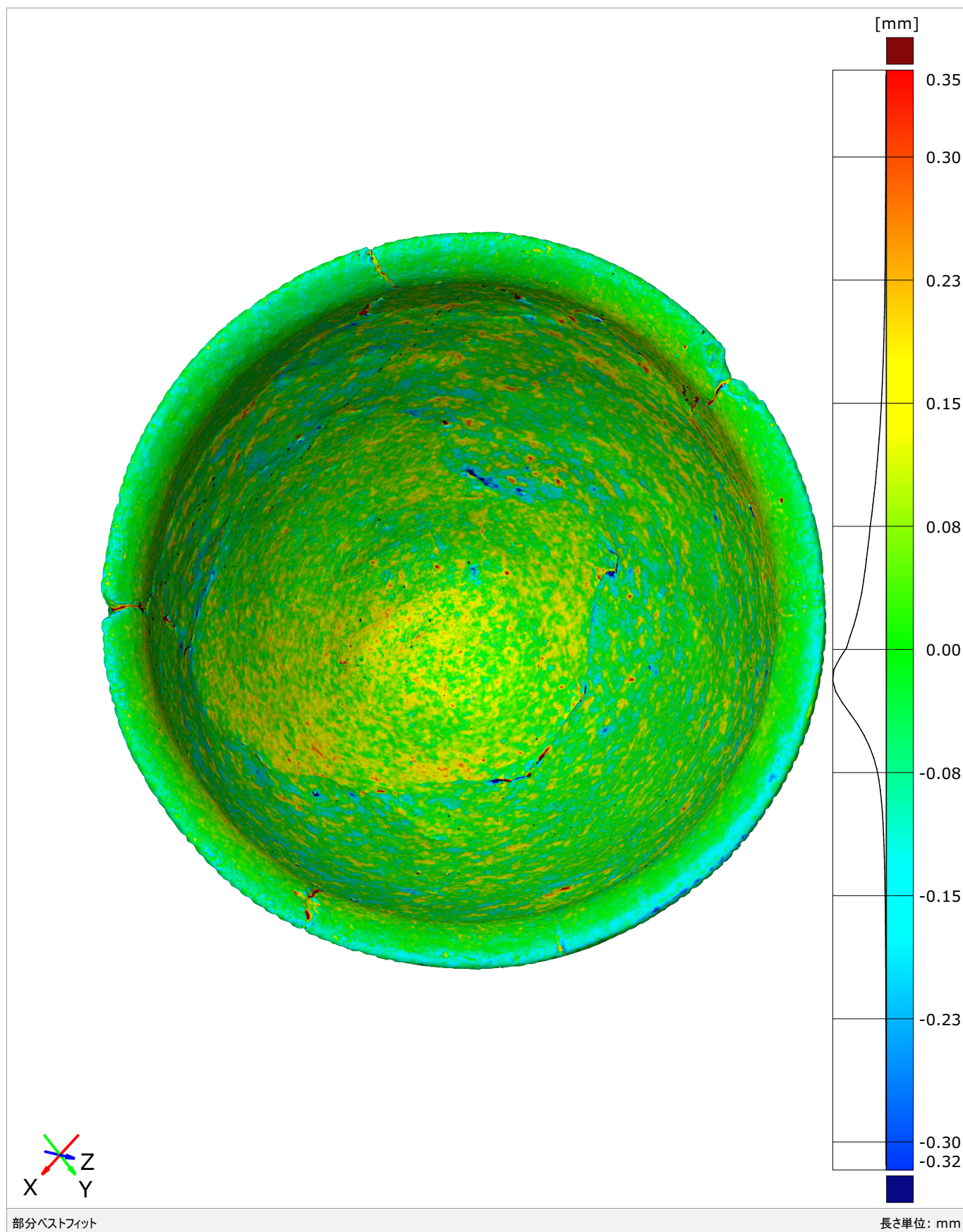

タイトルなし

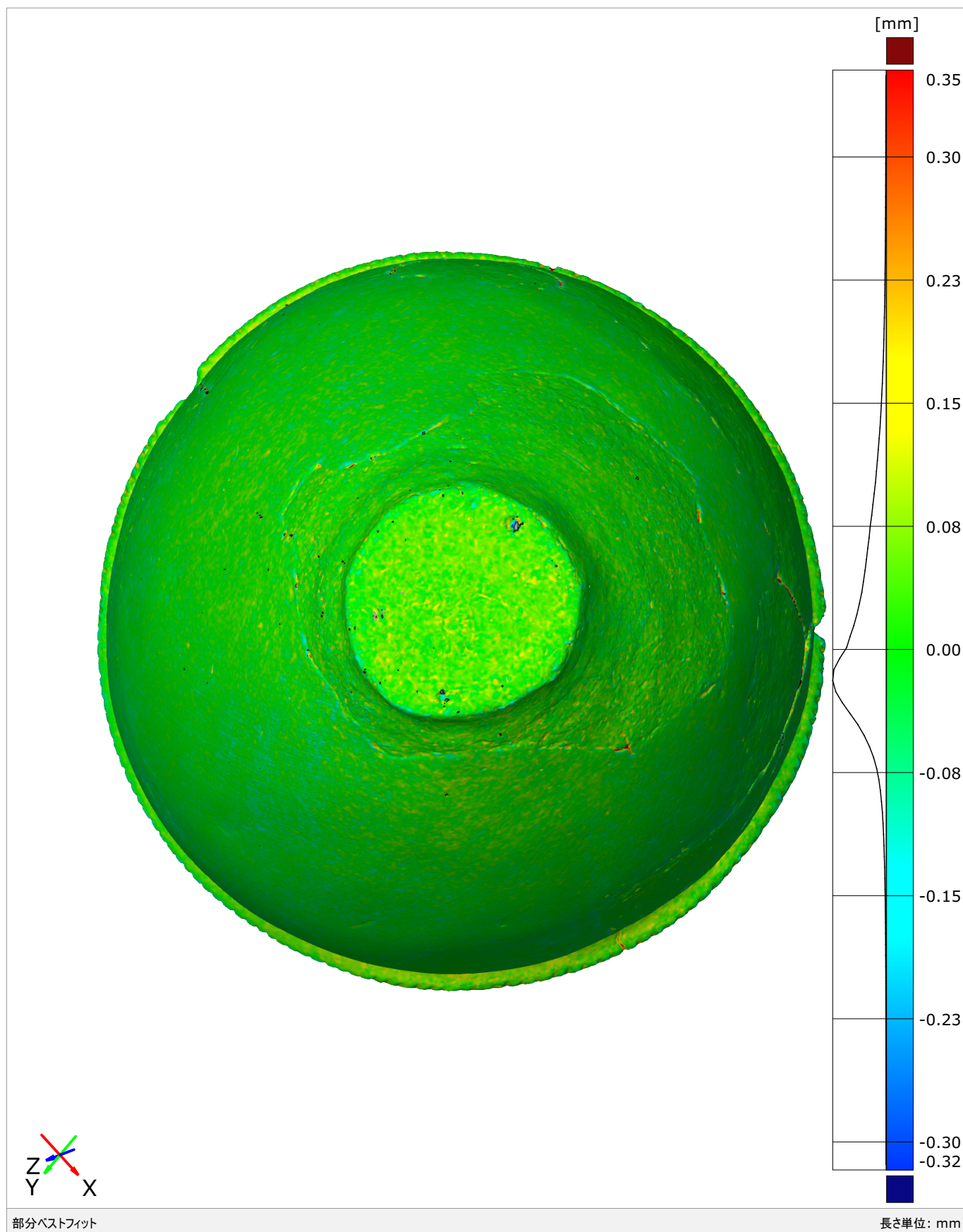

Supplement: S11 Fig — (PDF) [file pone.0270660.s011.pdf]

タイトルなし

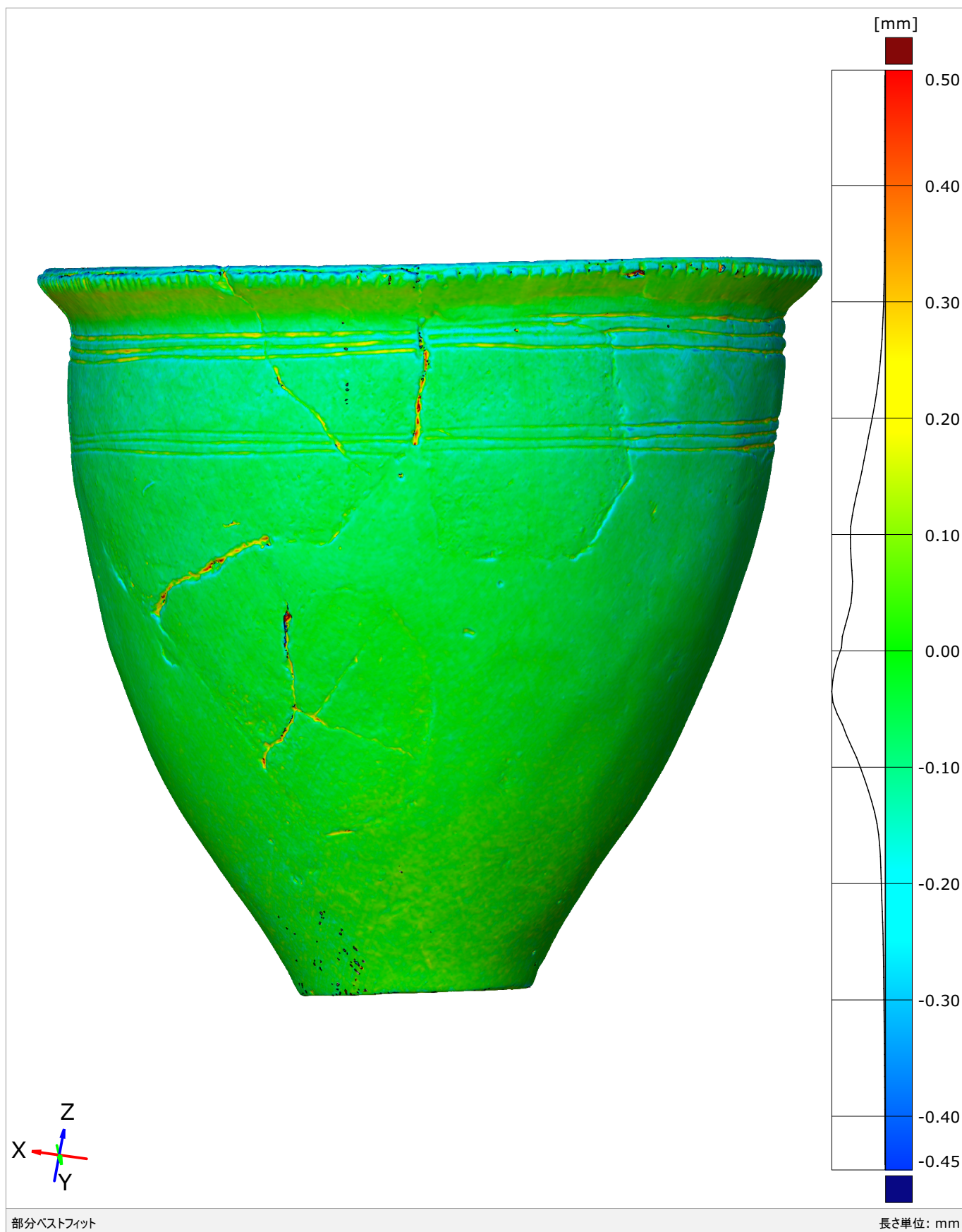

タイトルなし

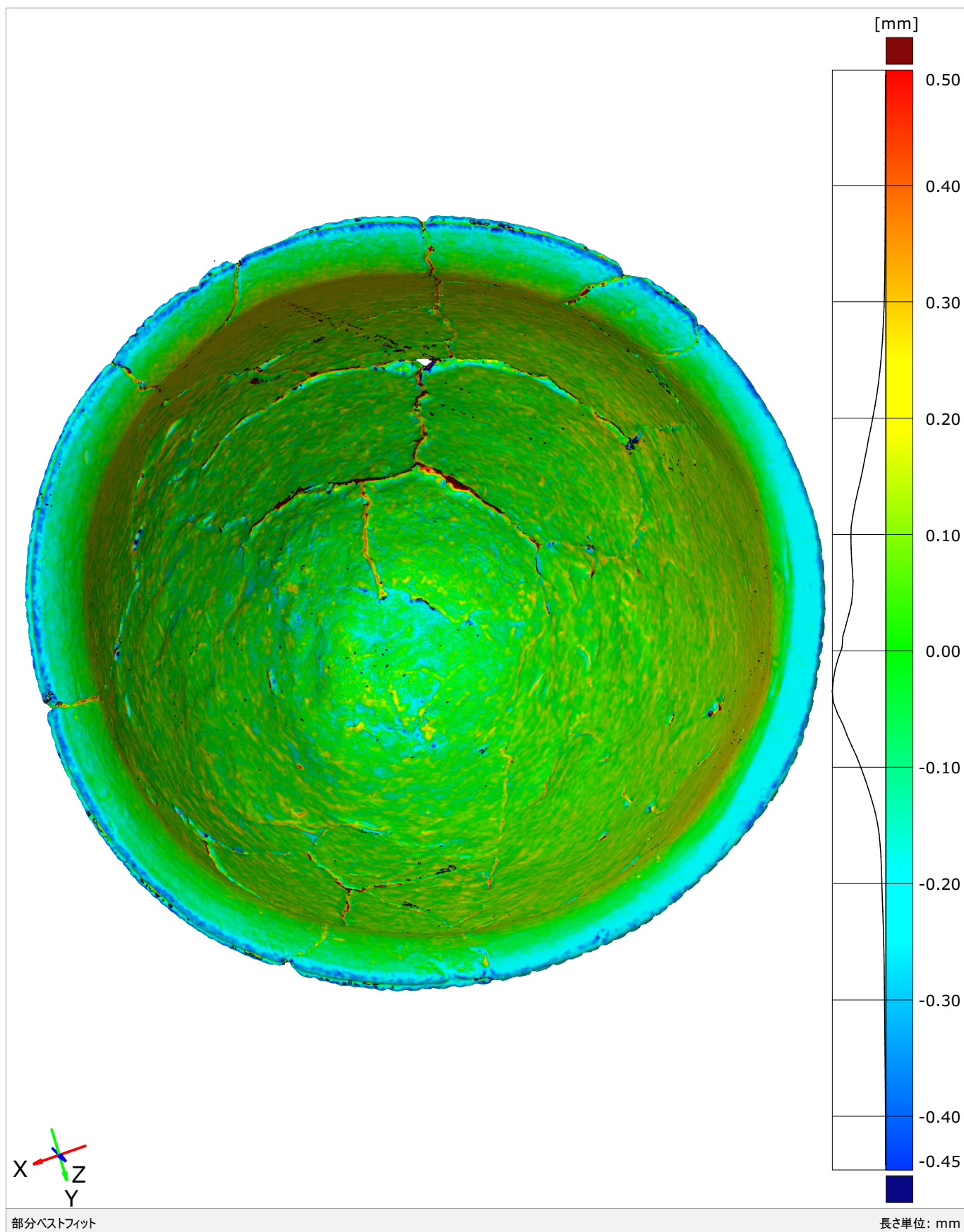

タイトルなし

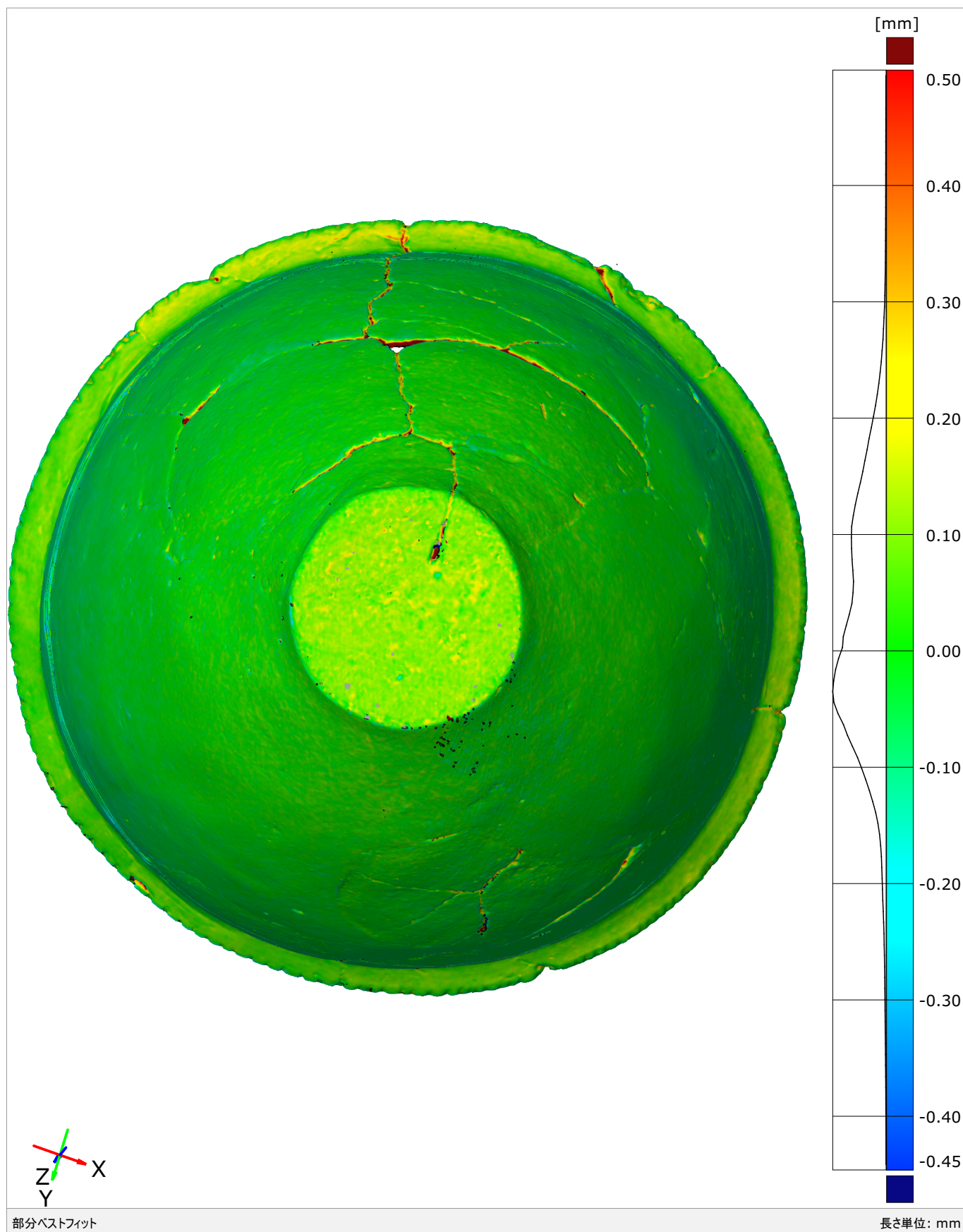

Supplement: S12 Fig — (PDF) [file pone.0270660.s012.pdf]

タイトルなし

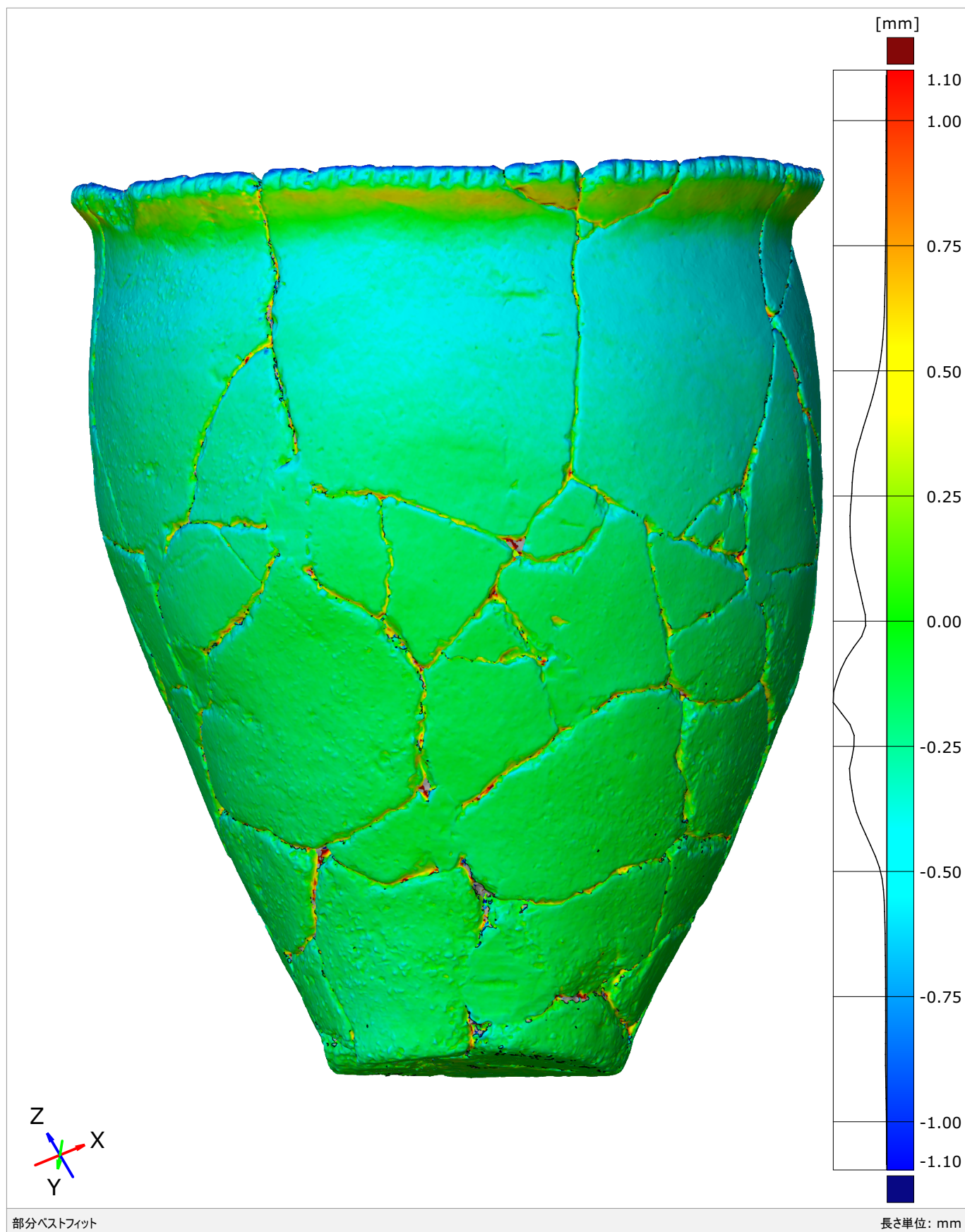

タイトルなし

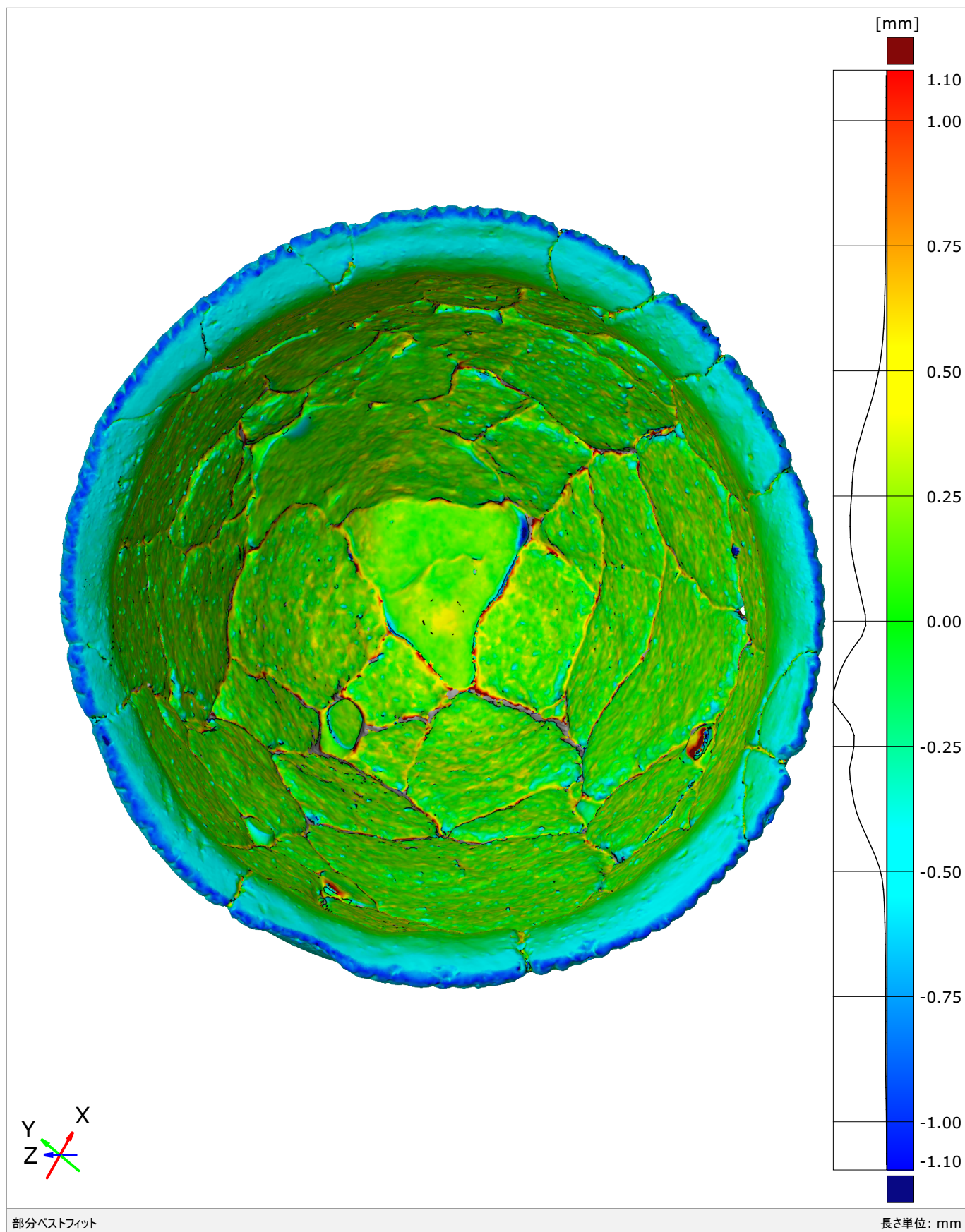

タイトルなし

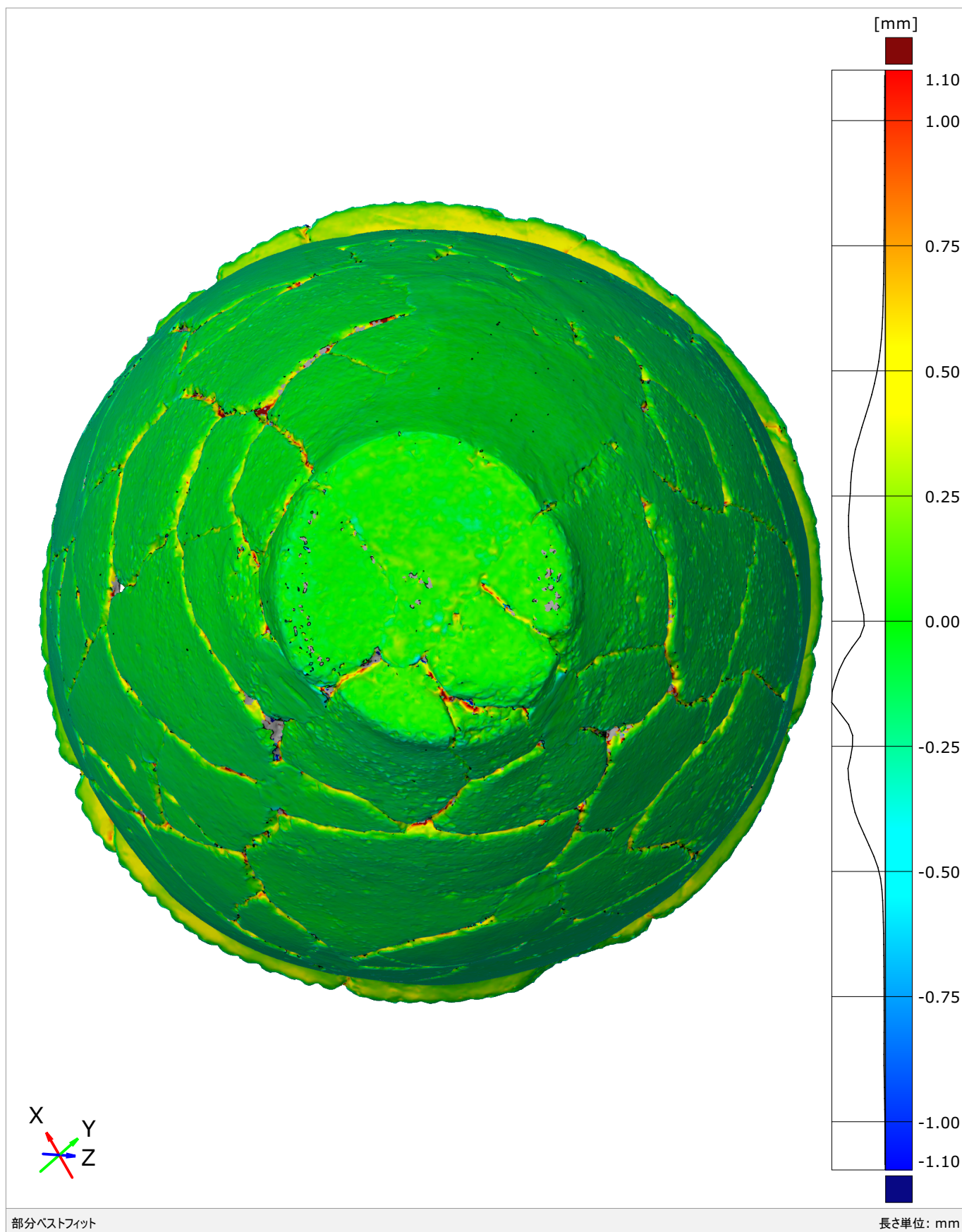

Supplement: S13 Fig — (PDF) [file pone.0270660.s013.pdf]

タイトルなし

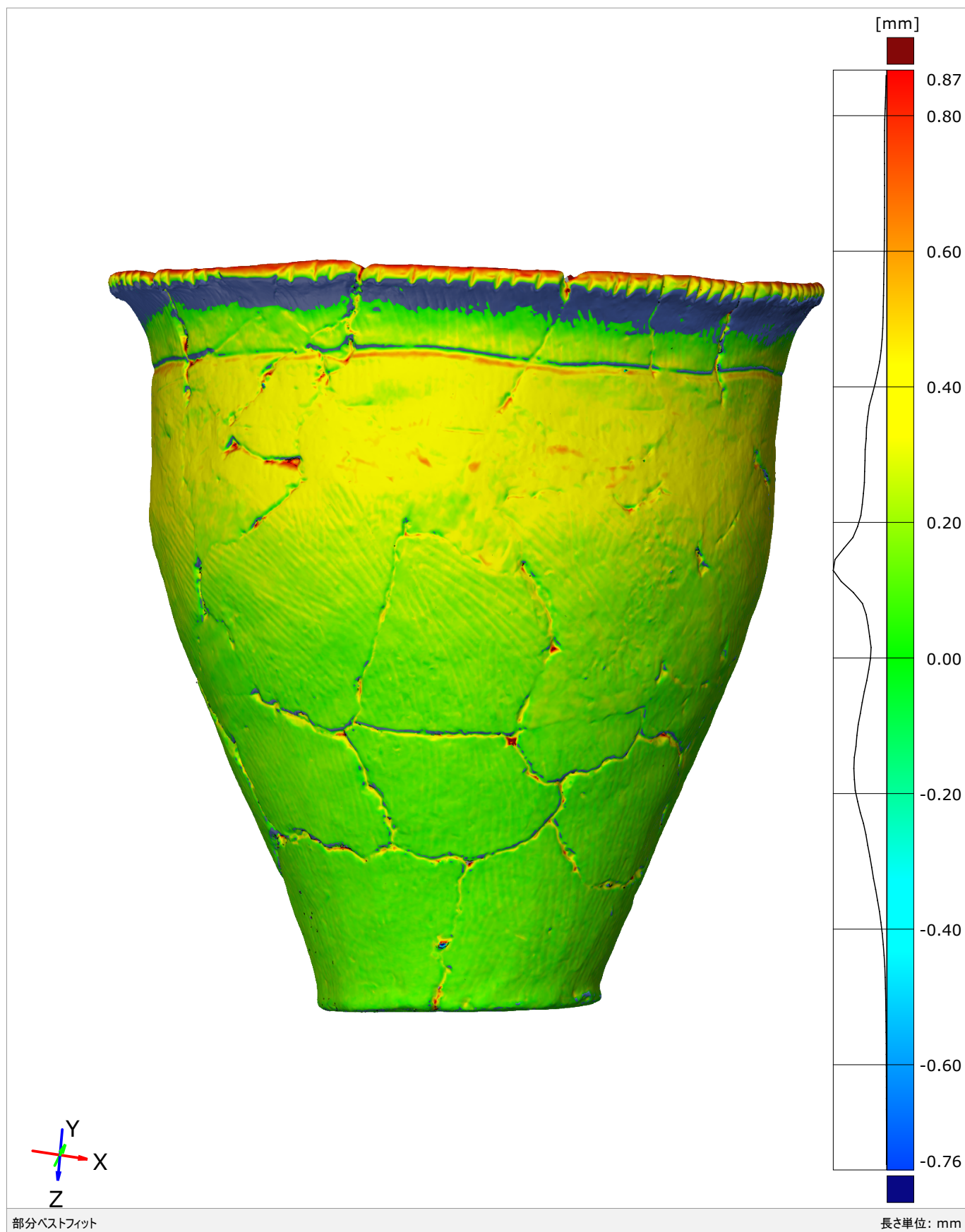

タイトルなし

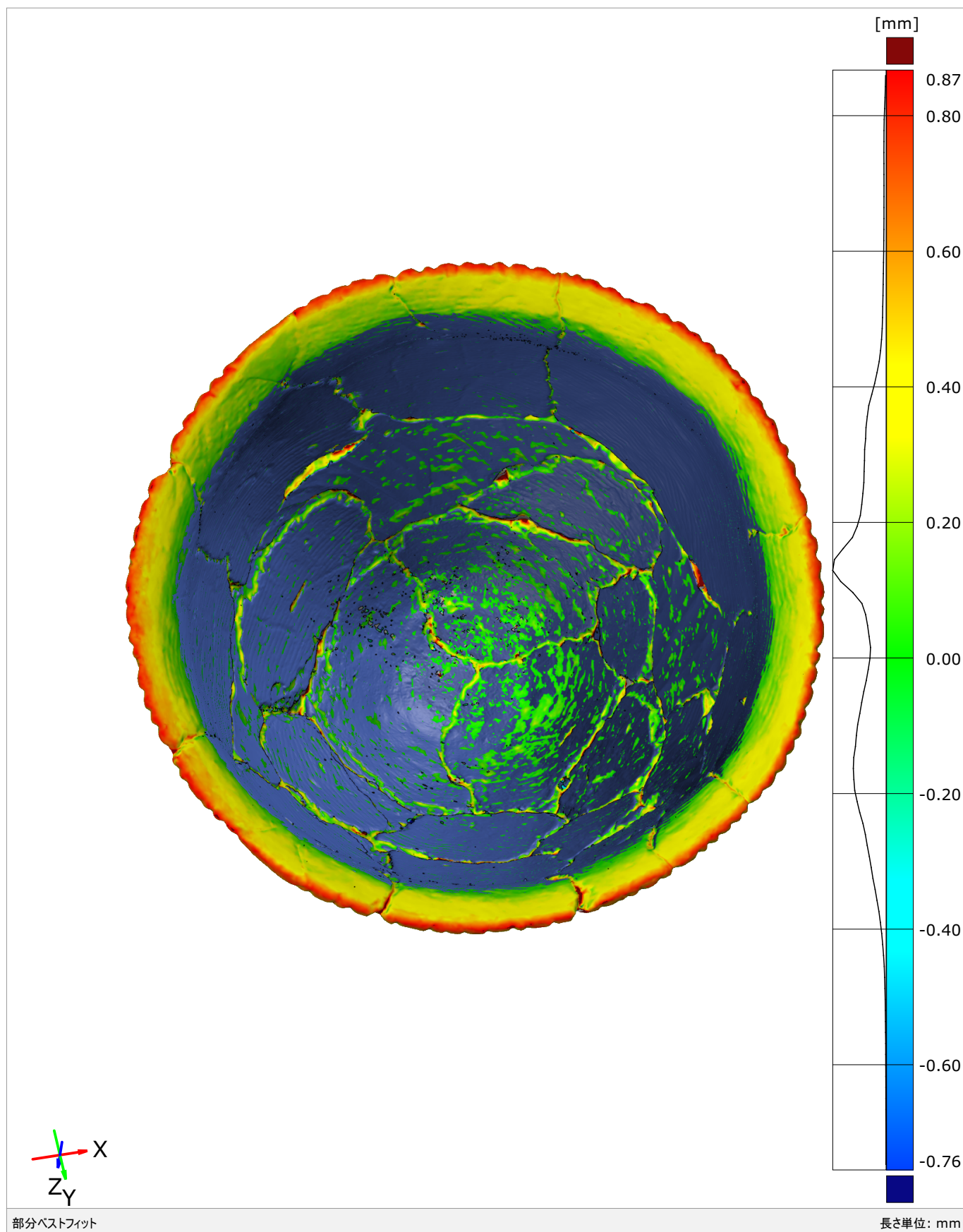

タイトルなし

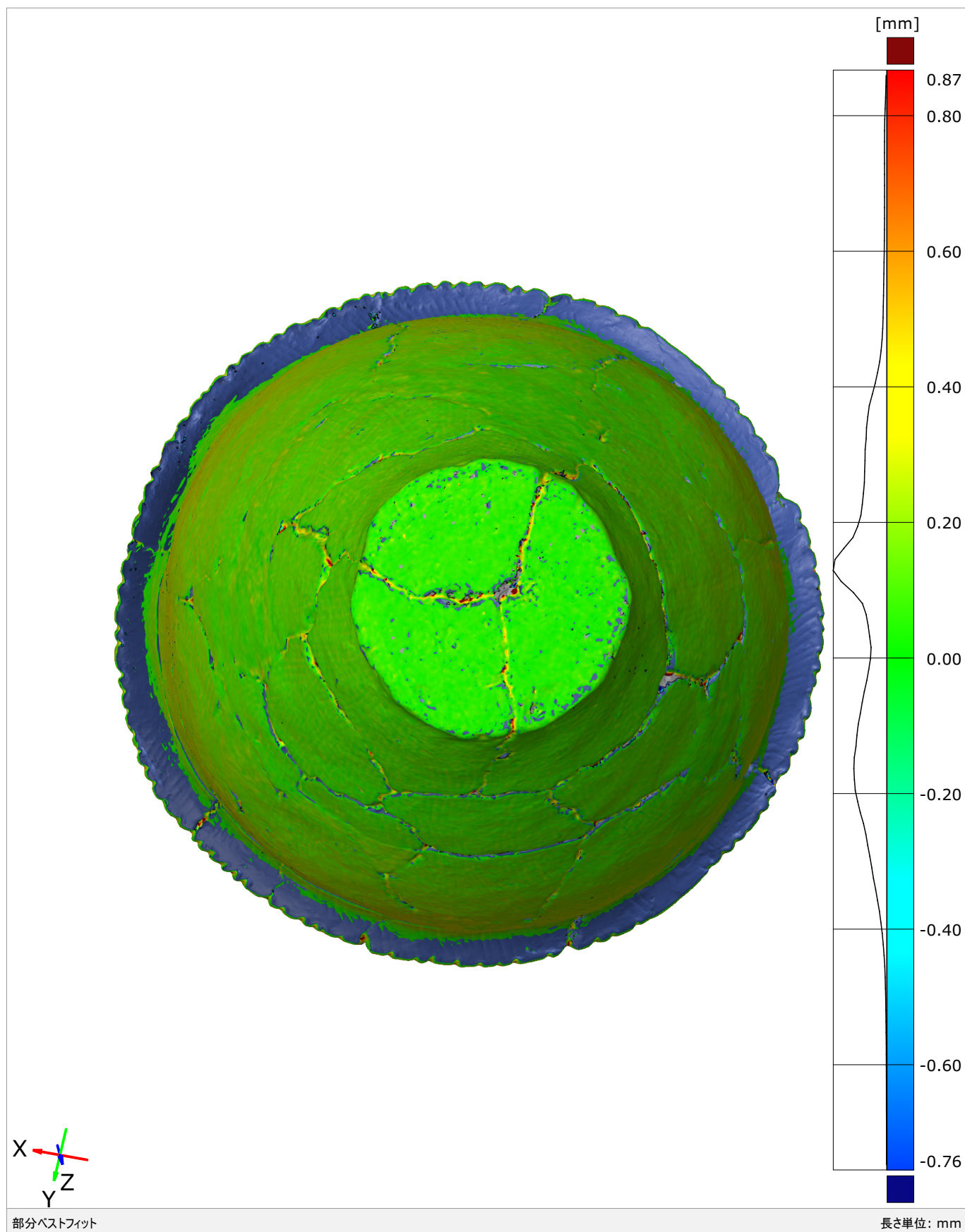

Supplement: S14 Fig — (PDF) [file pone.0270660.s014.pdf]

タイトルなし

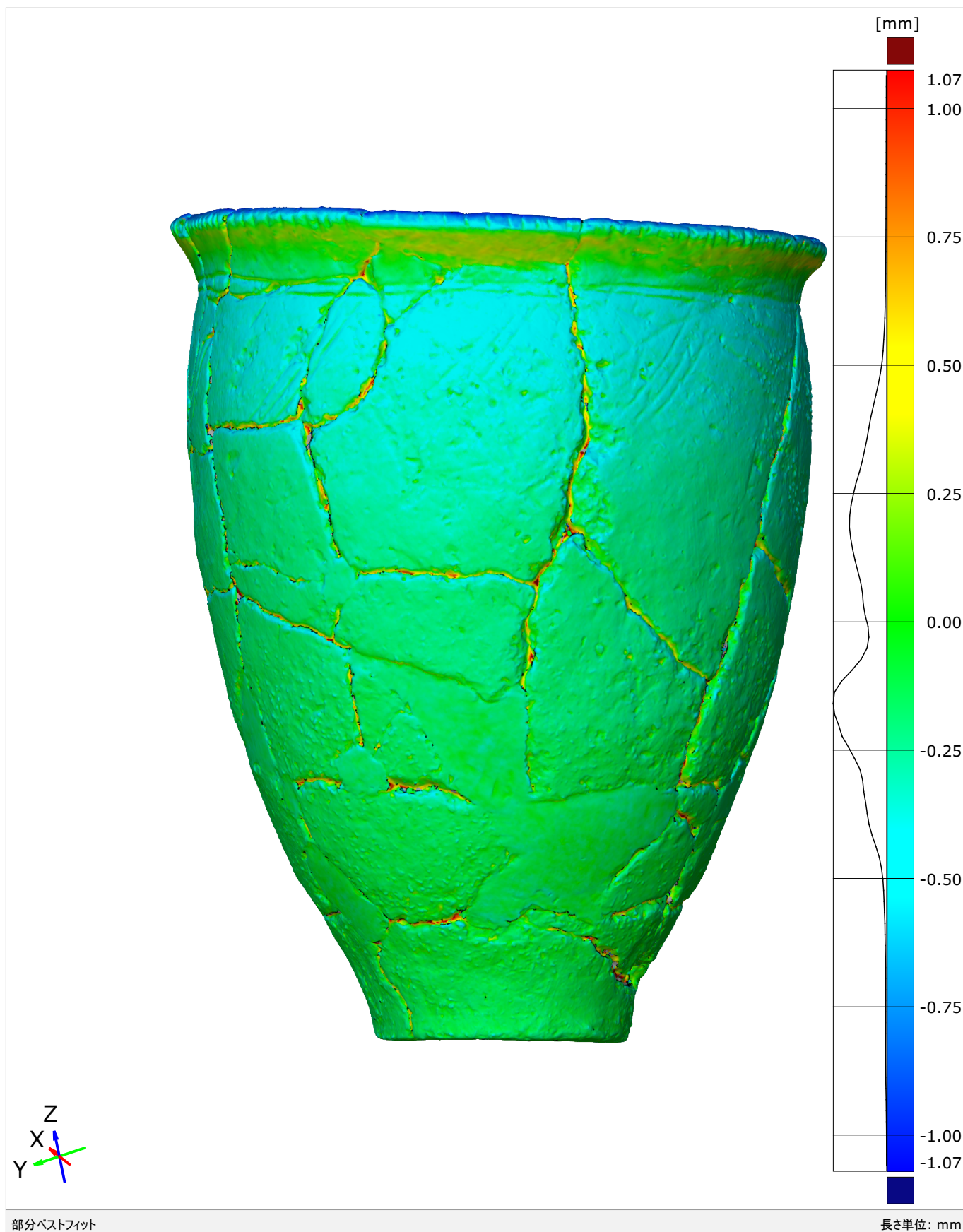

タイトルなし

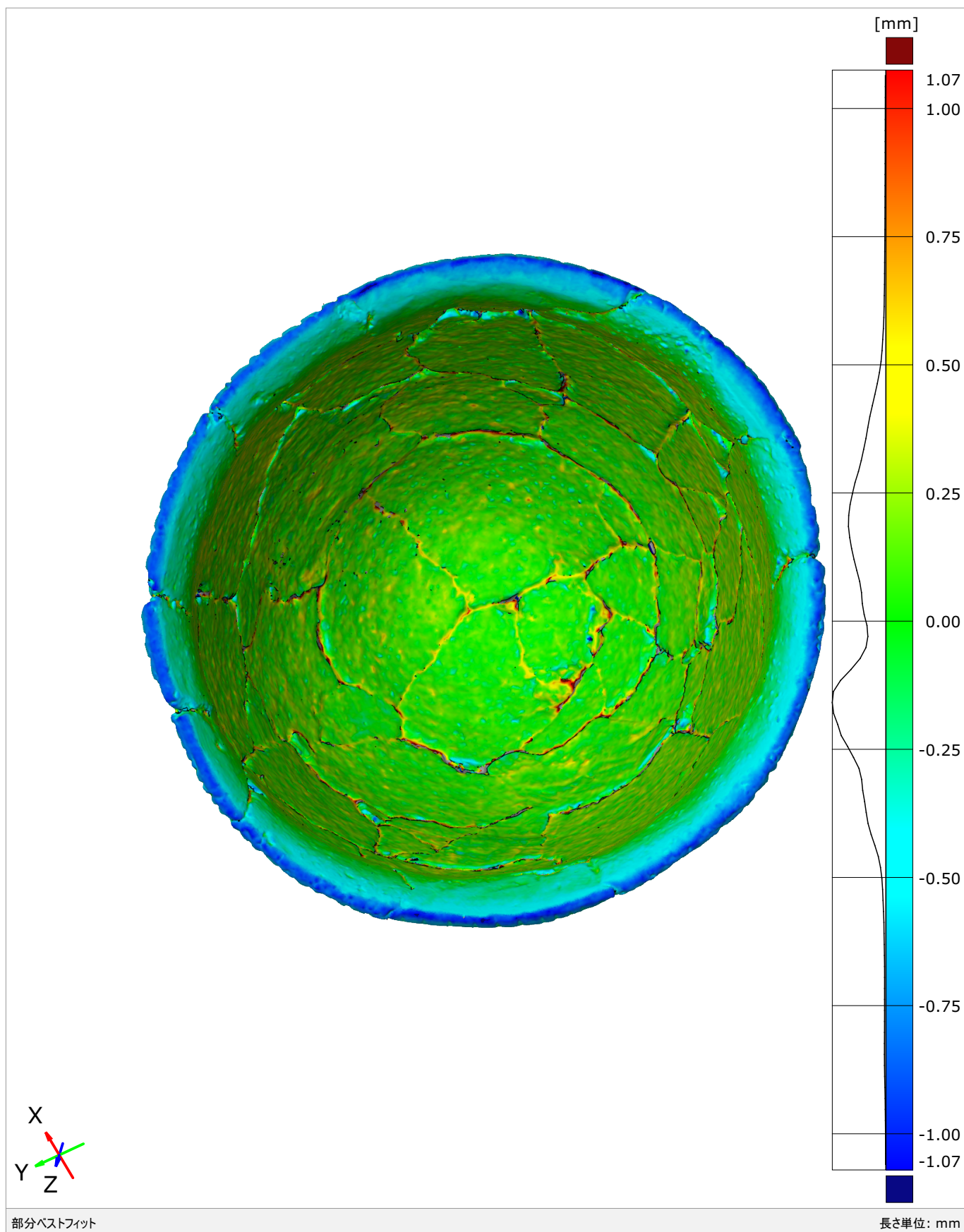

タイトルなし

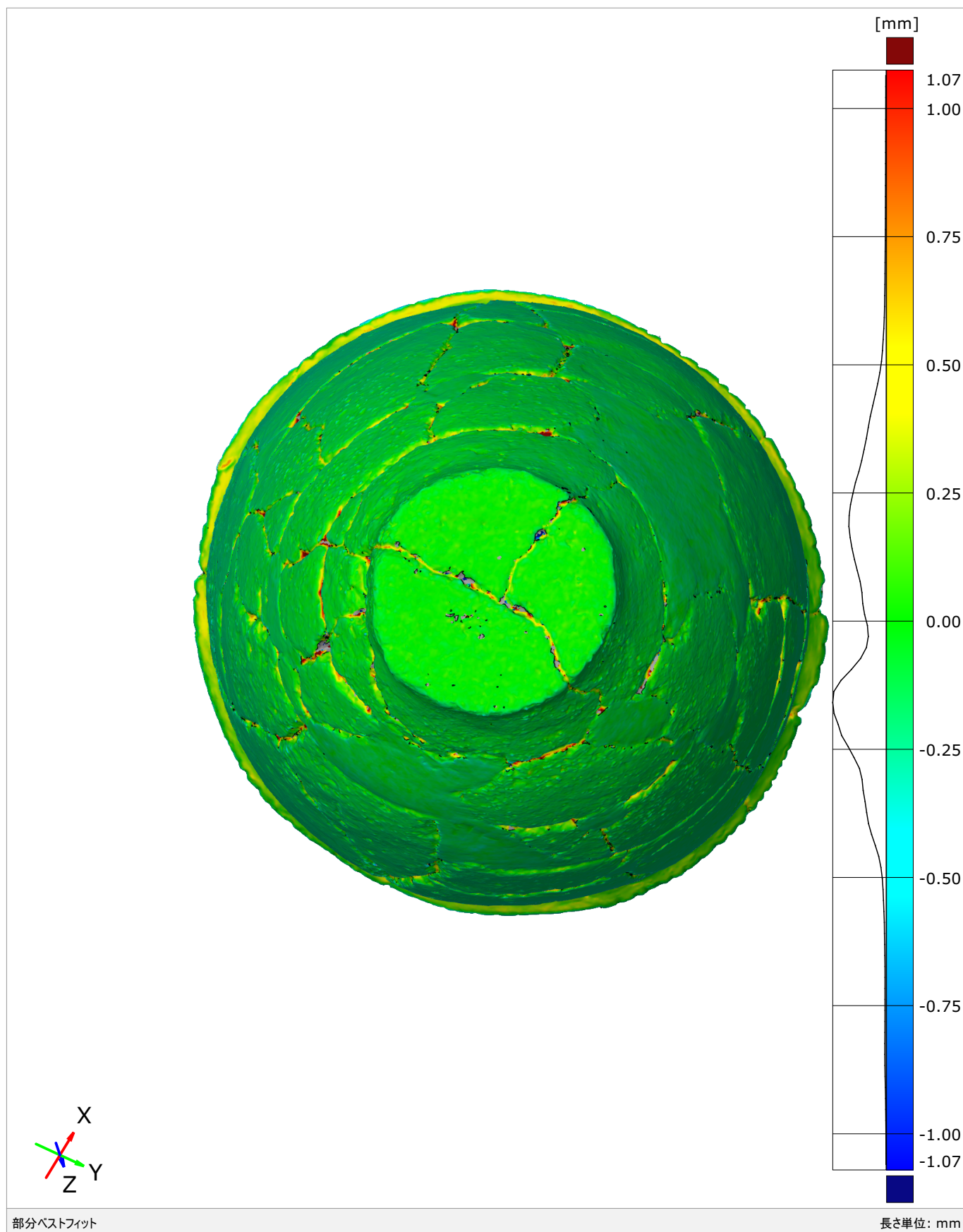

Supplement: S15 Fig — (PDF) [file pone.0270660.s015.pdf]

タイトルなし

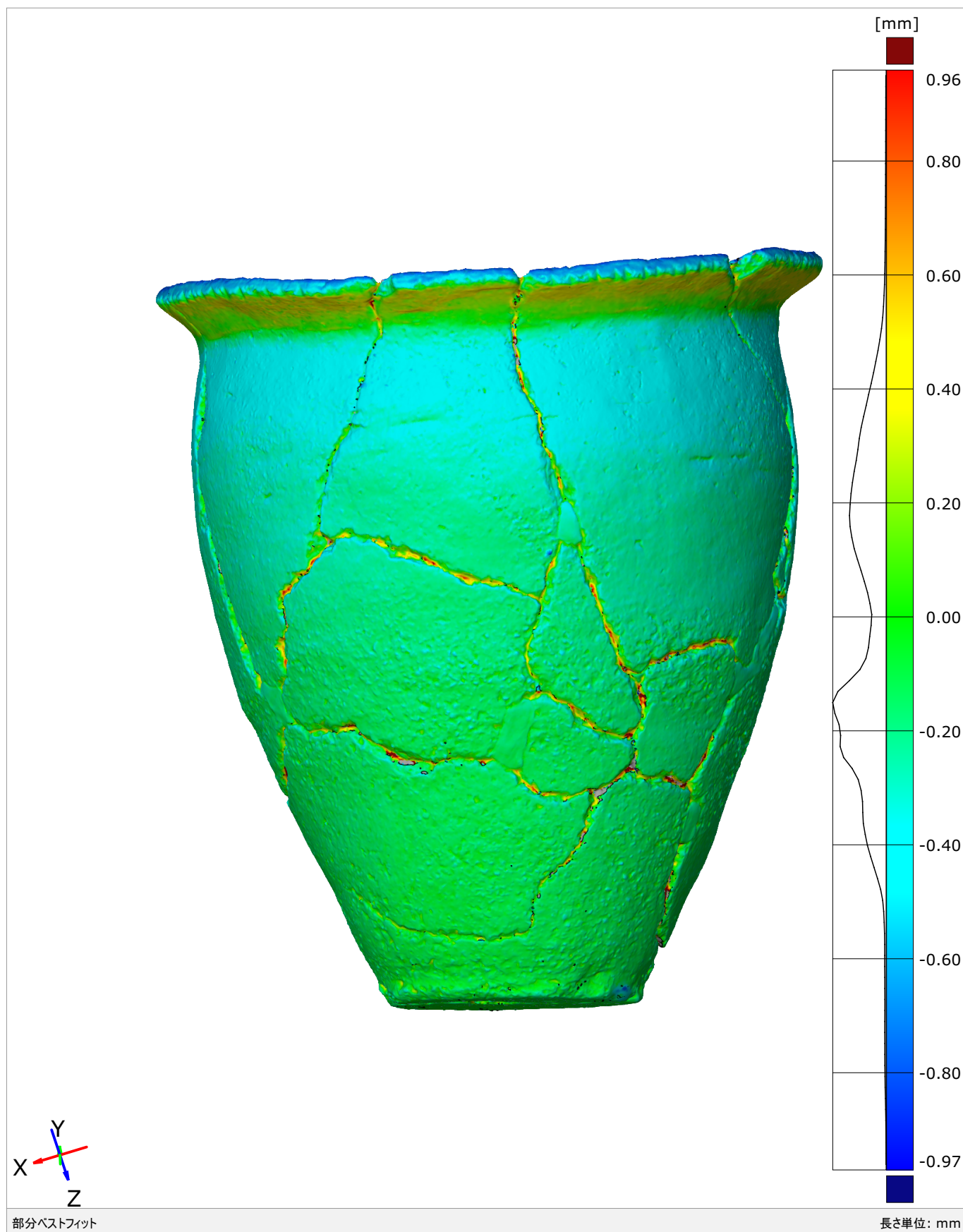

タイトルなし

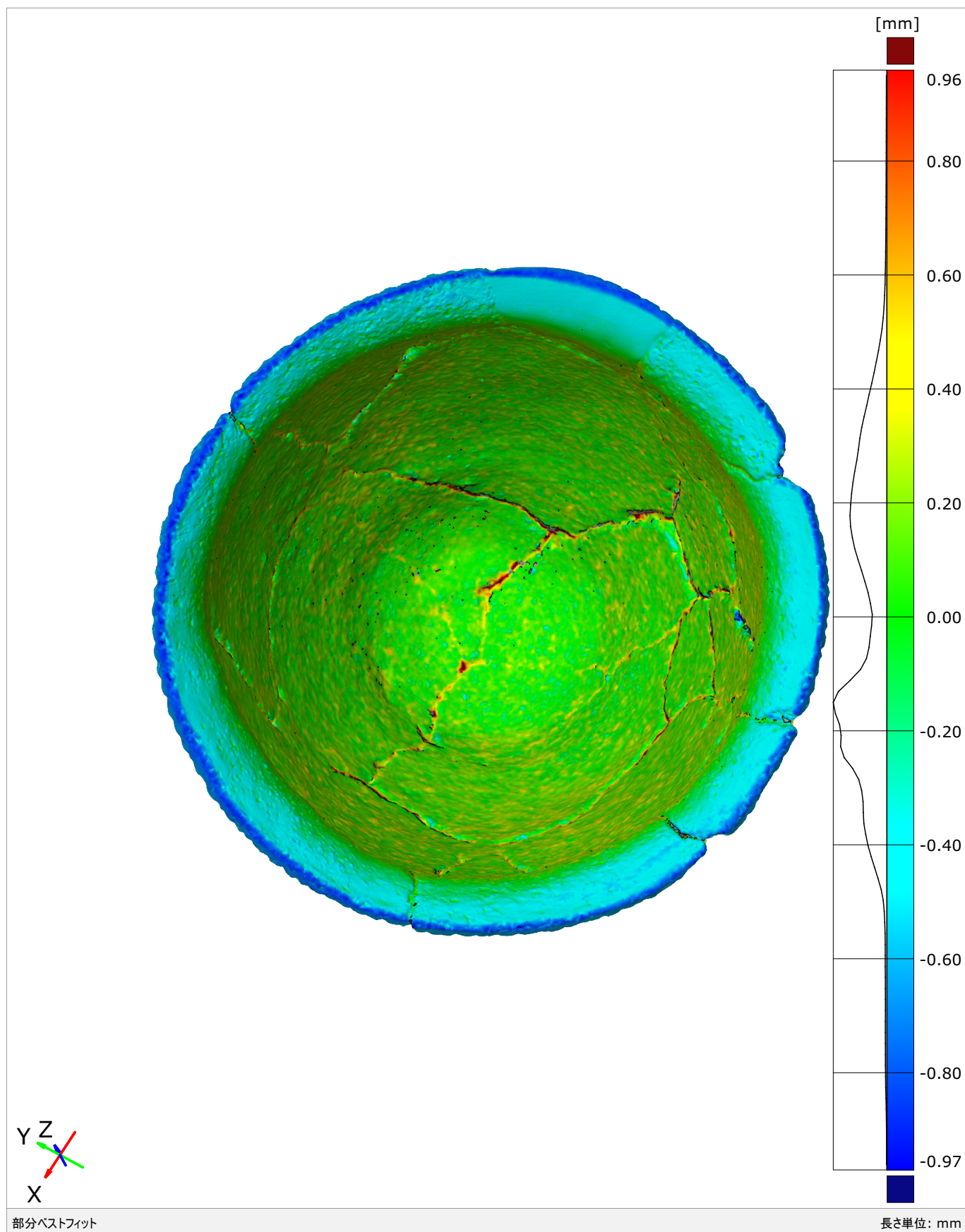

タイトルなし

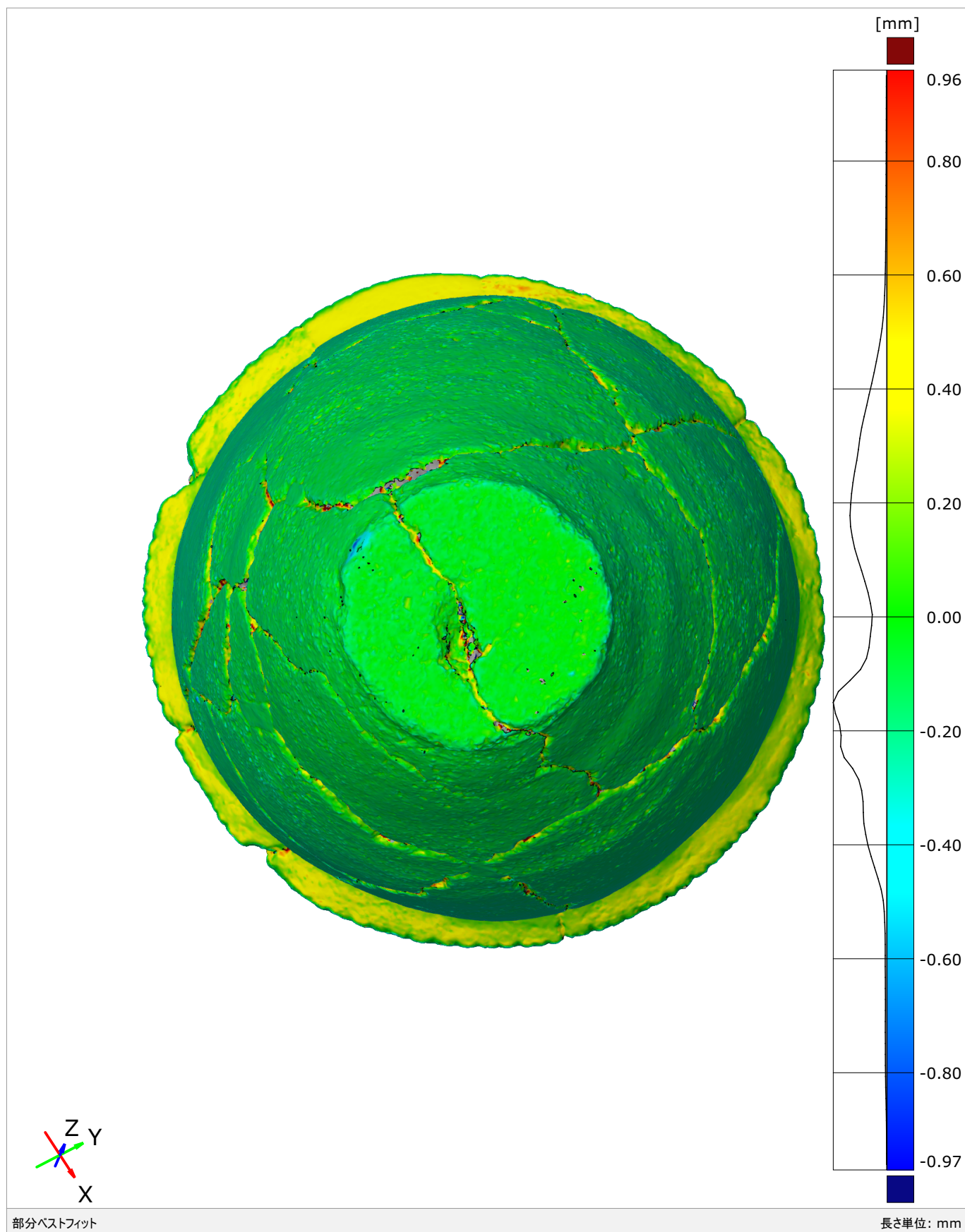

Supplement: S16 Fig — (PDF) [file pone.0270660.s016.pdf]

タイトルなし

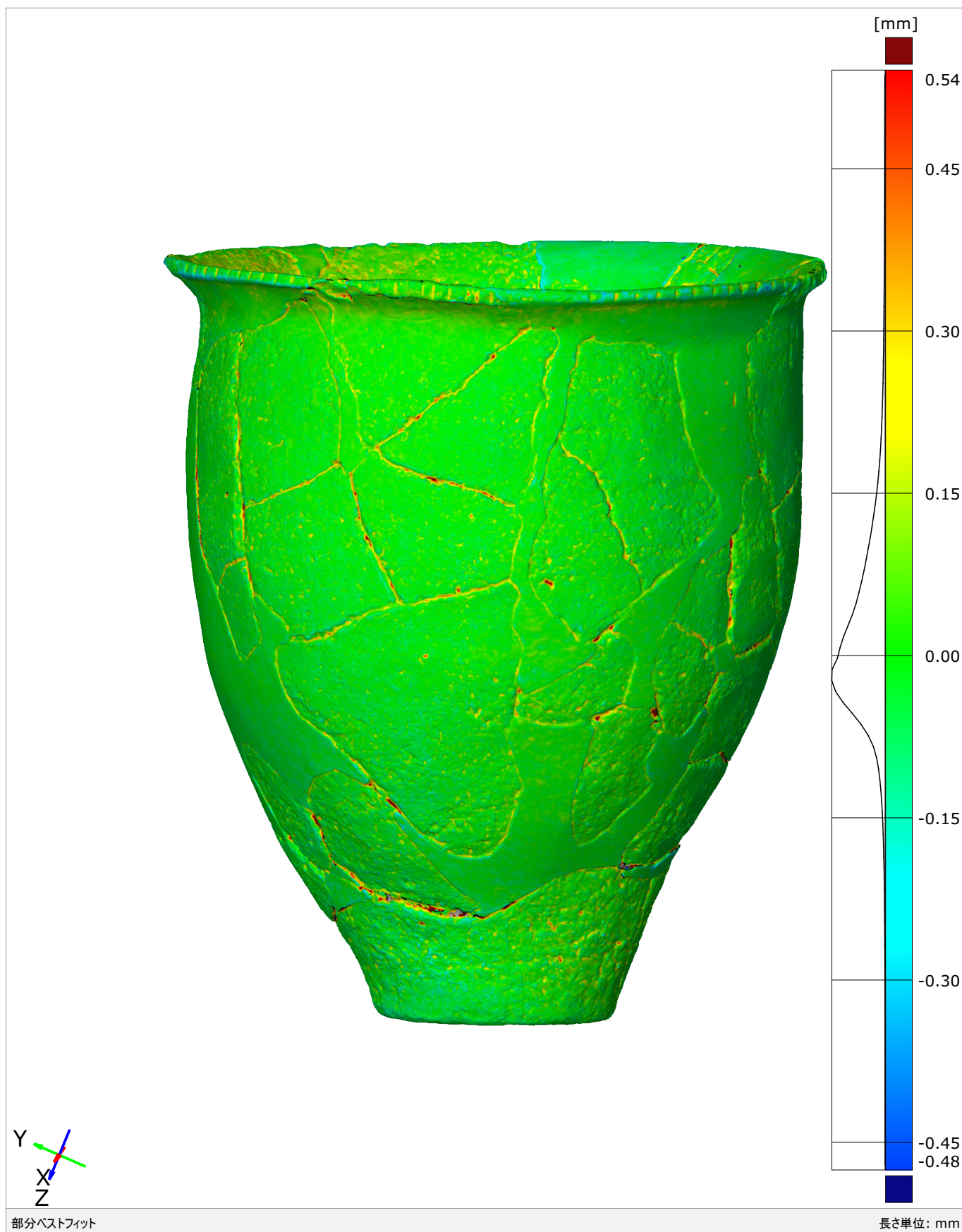

タイトルなし

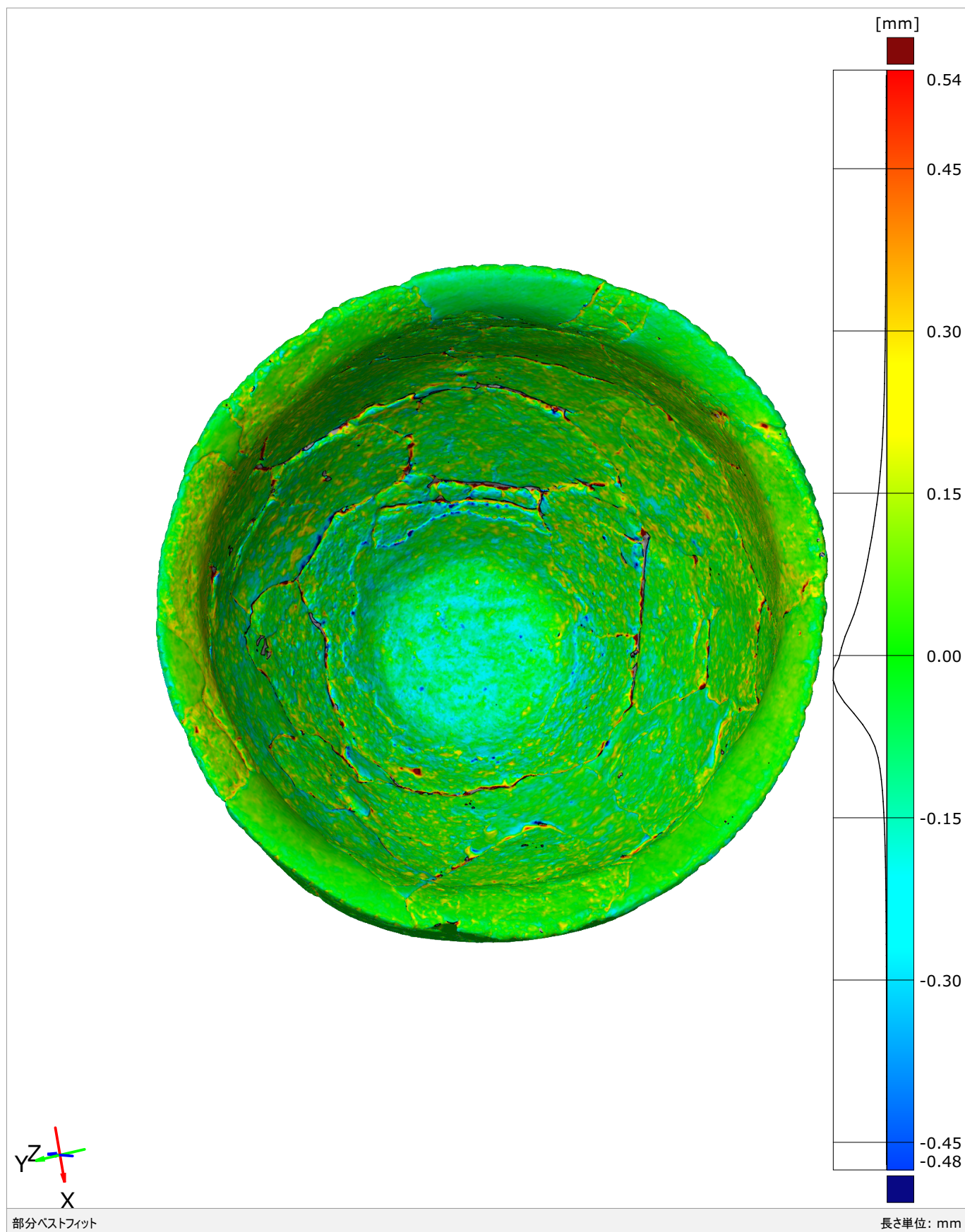

タイトルなし

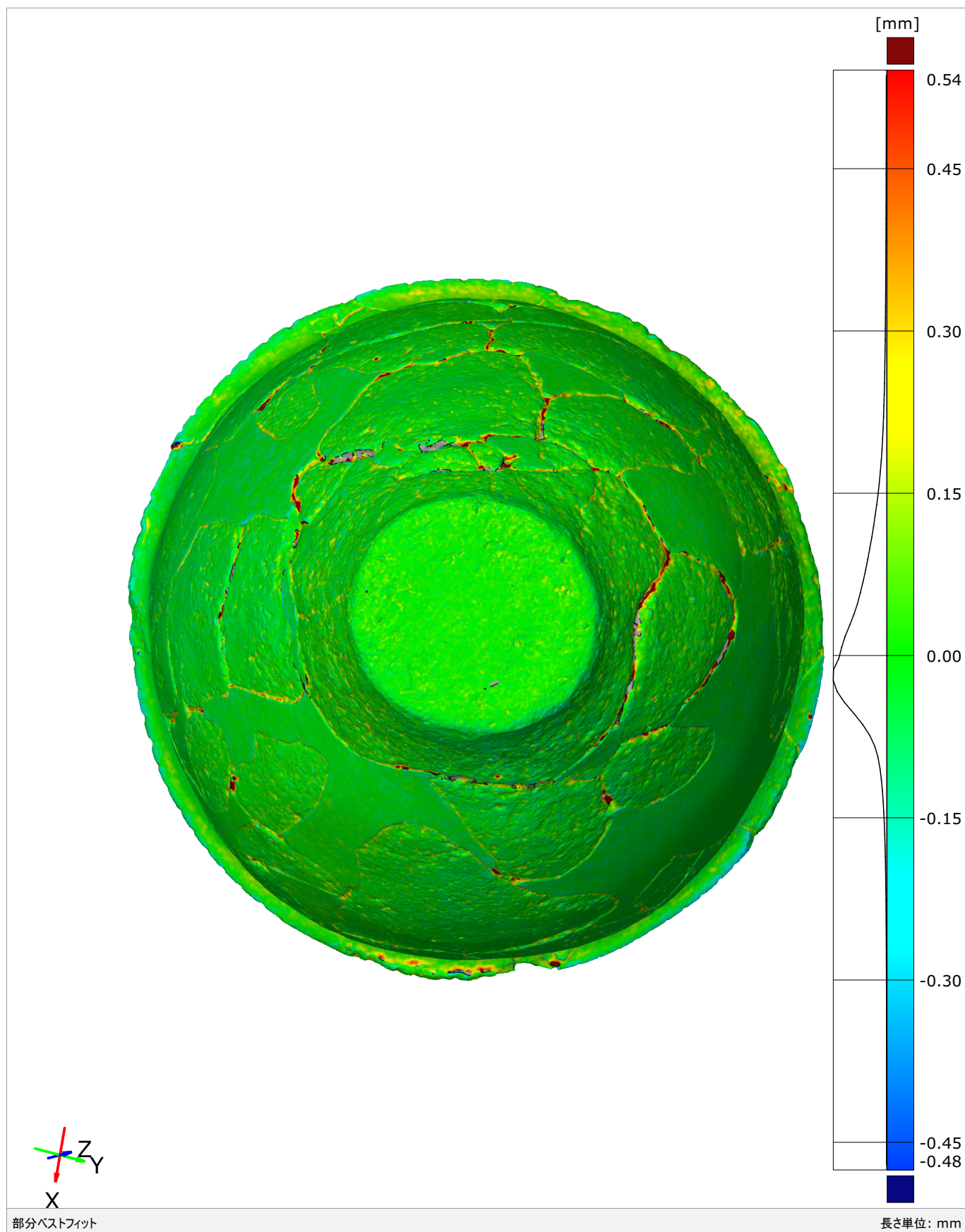

Supplement: S17 Fig — (PDF) [file pone.0270660.s017.pdf]

タイトルなし

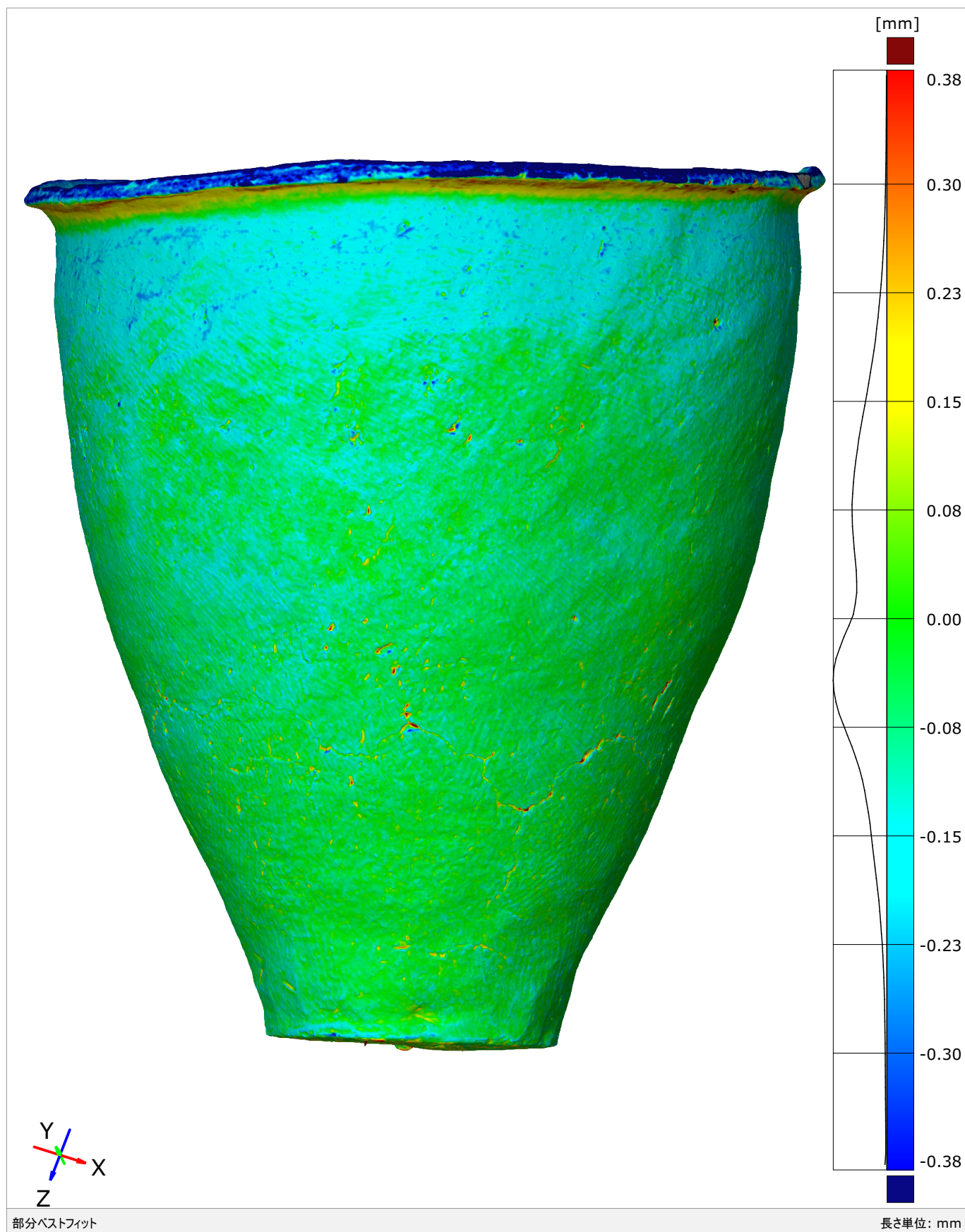

タイトルなし

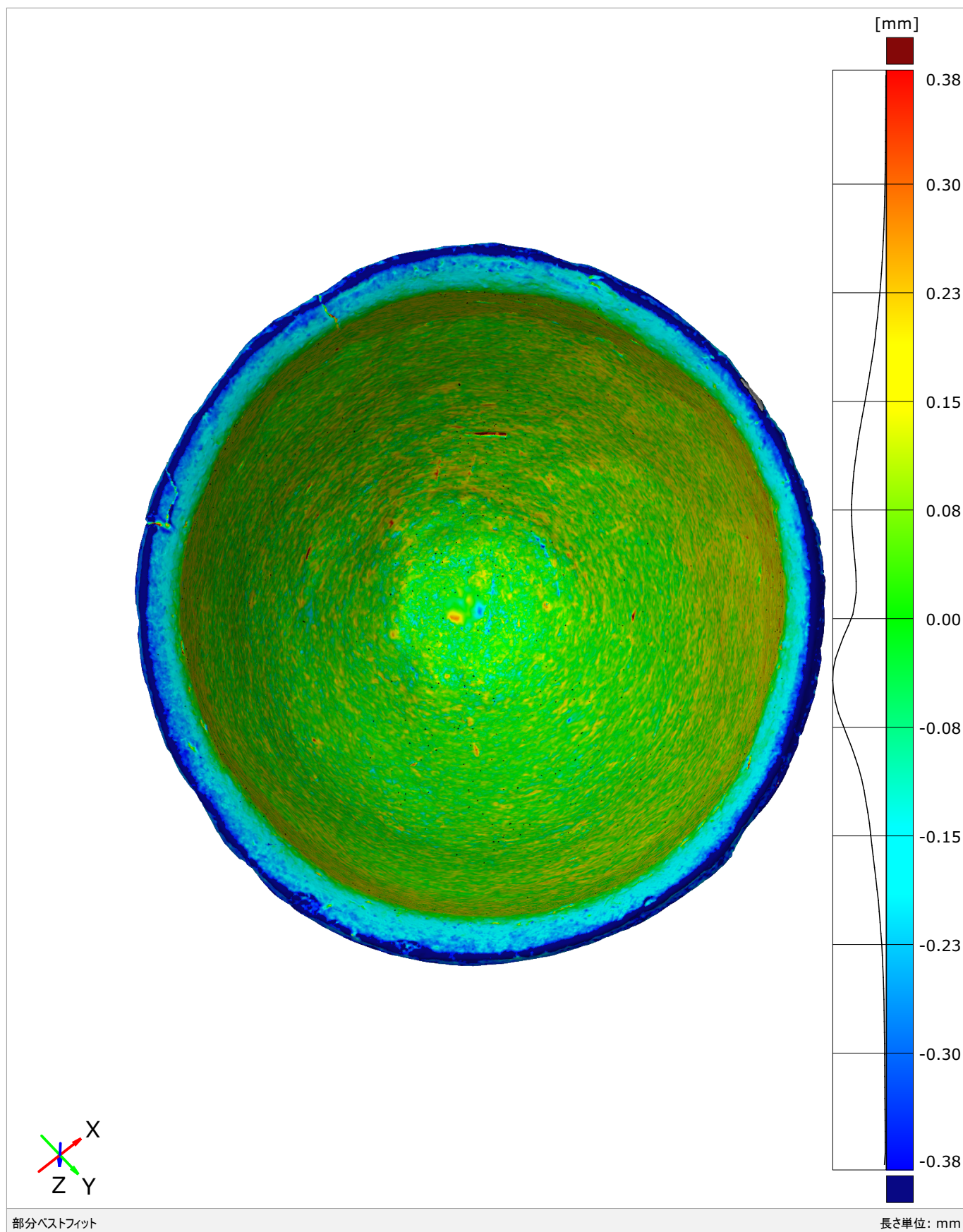

タイトルなし

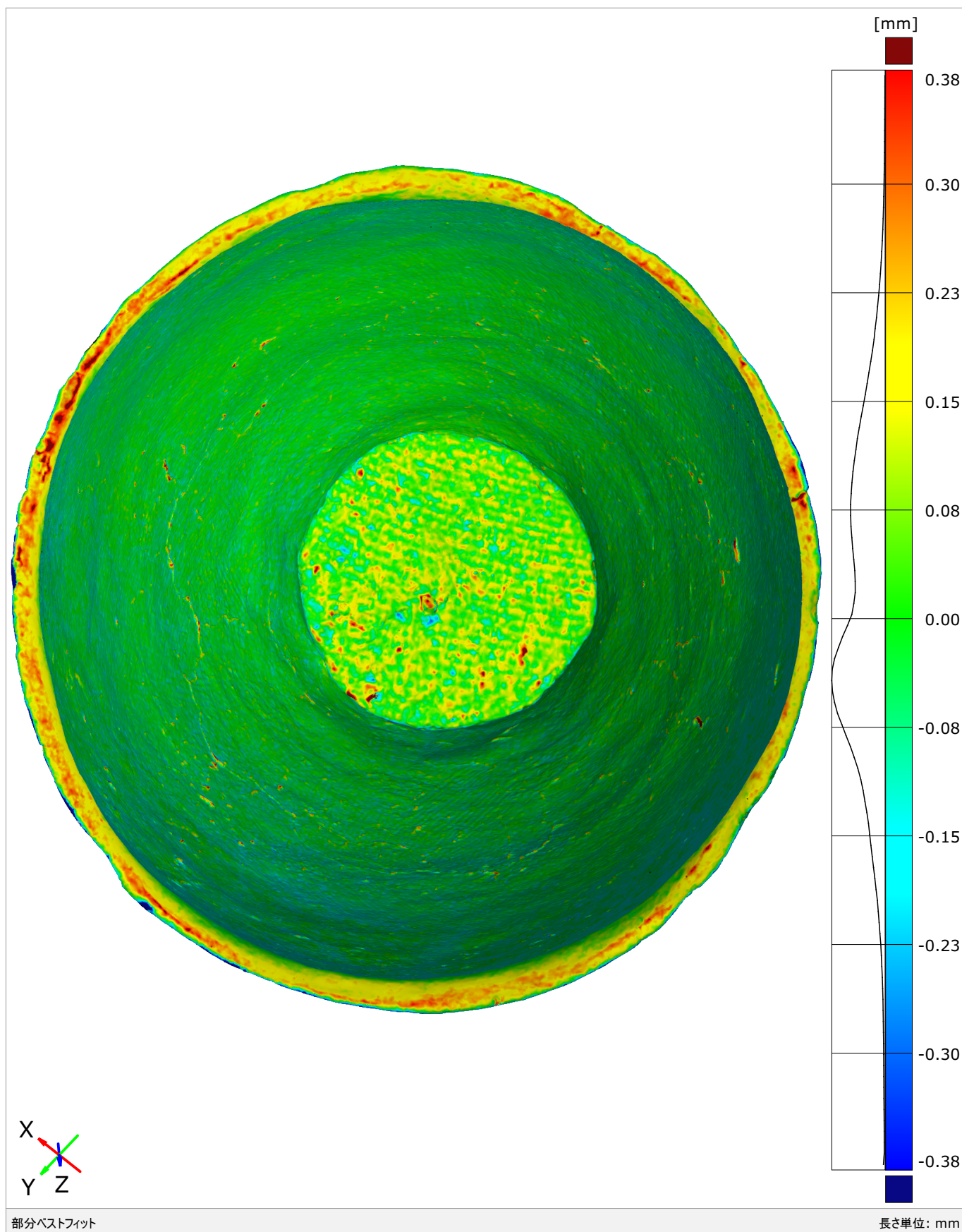

Supplement: S18 Fig — (PDF) [file pone.0270660.s018.pdf]

タイトルなし

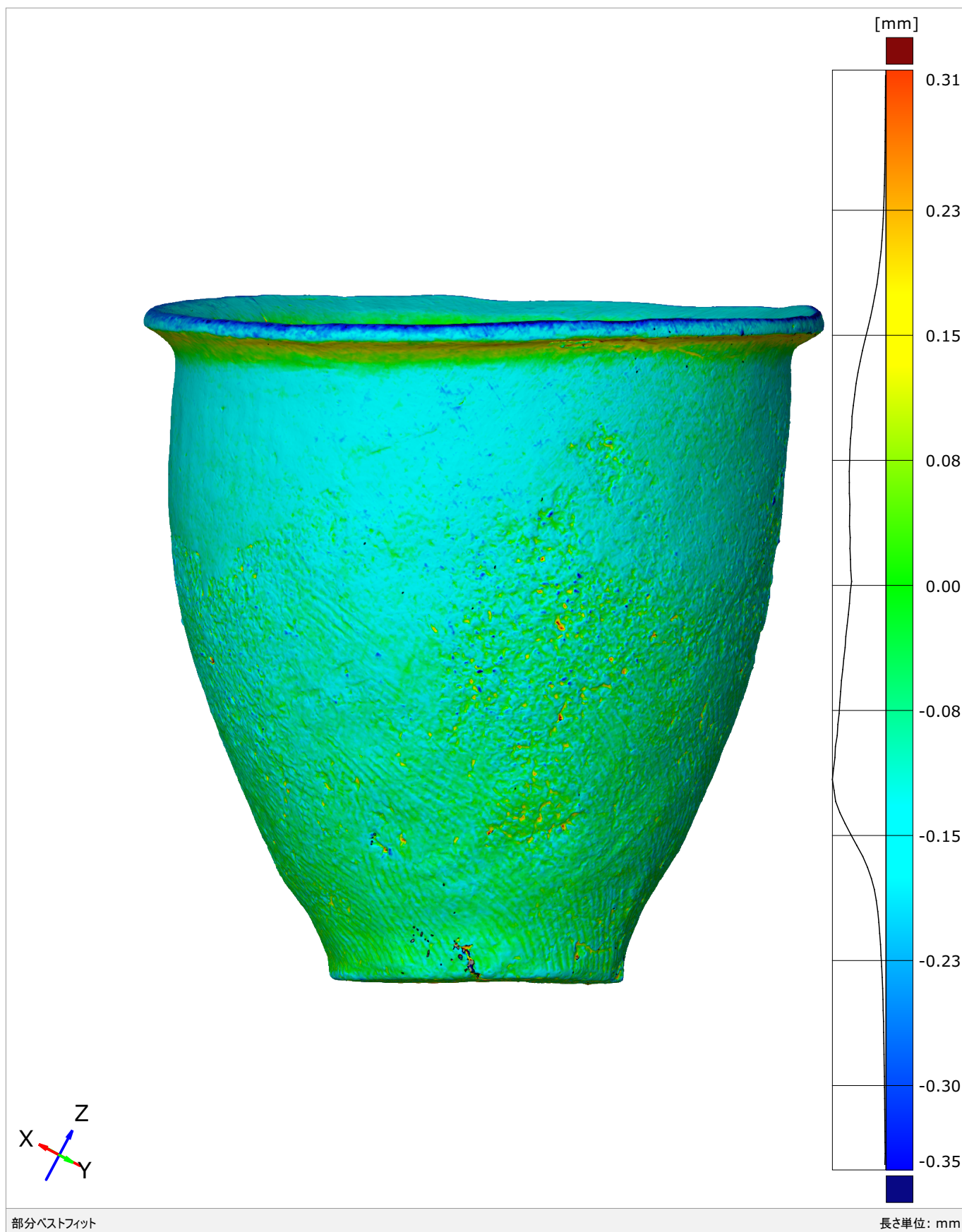

タイトルなし

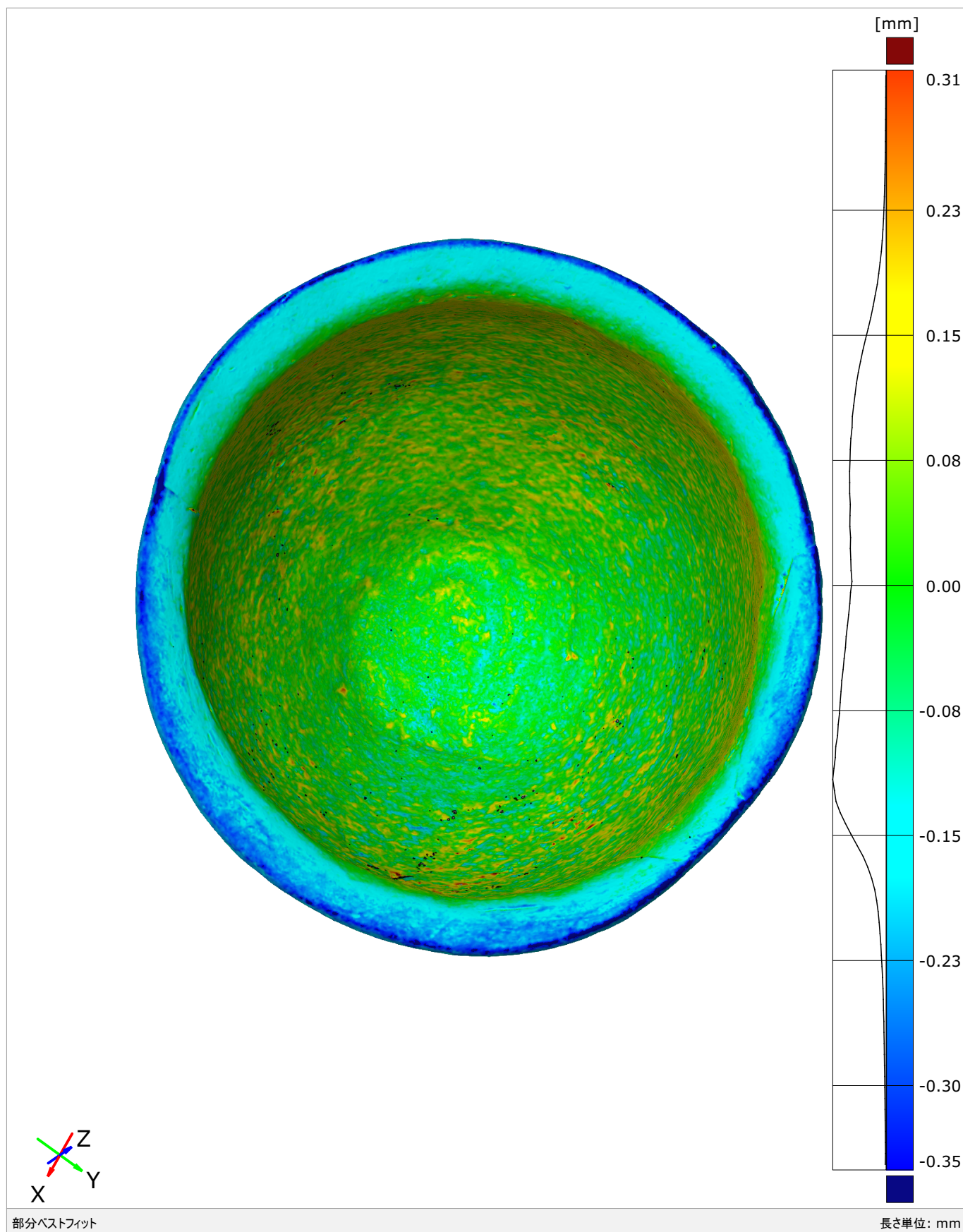

タイトルなし

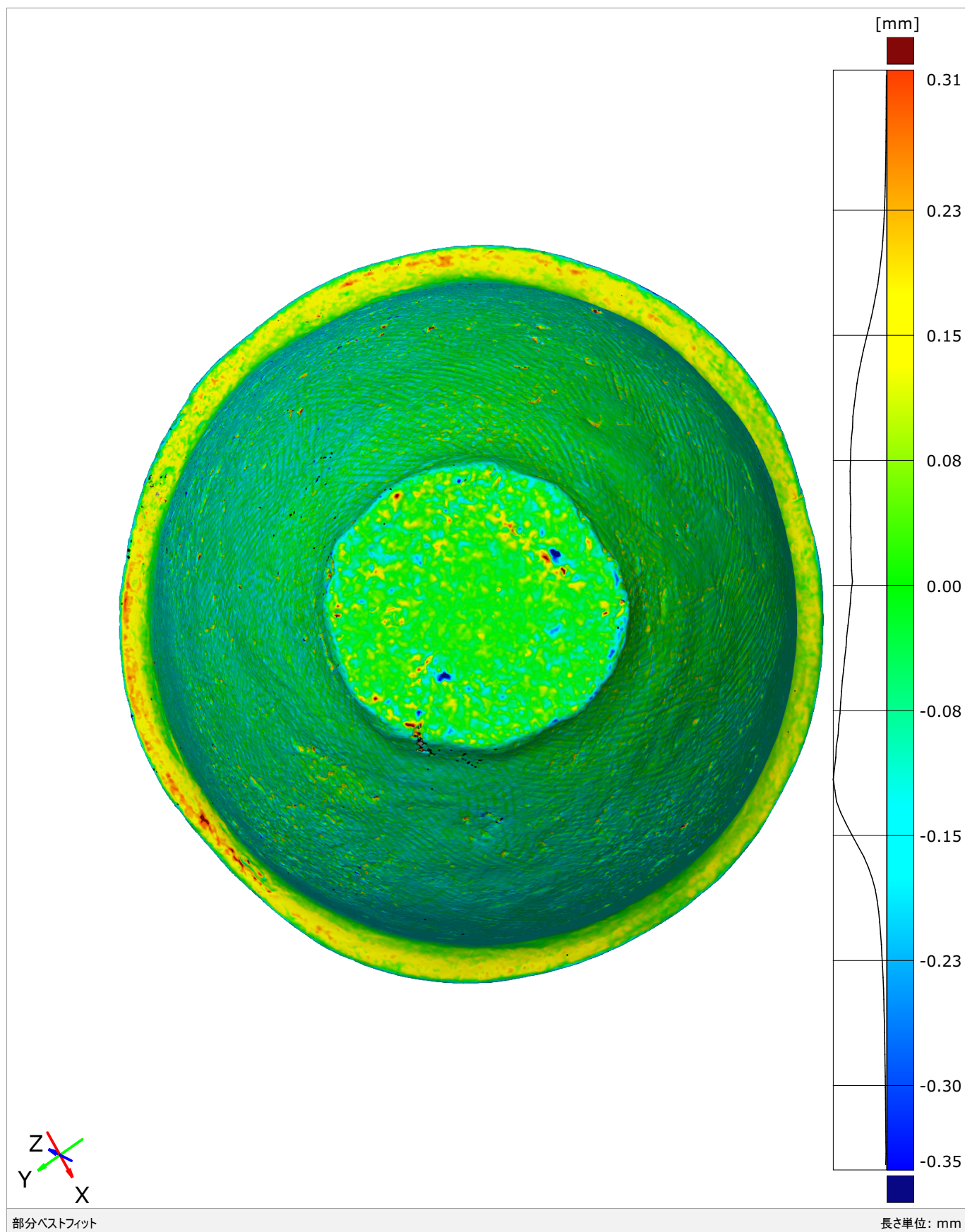

Supplement: S19 Fig — (PDF) [file pone.0270660.s019.pdf]

タイトルなし

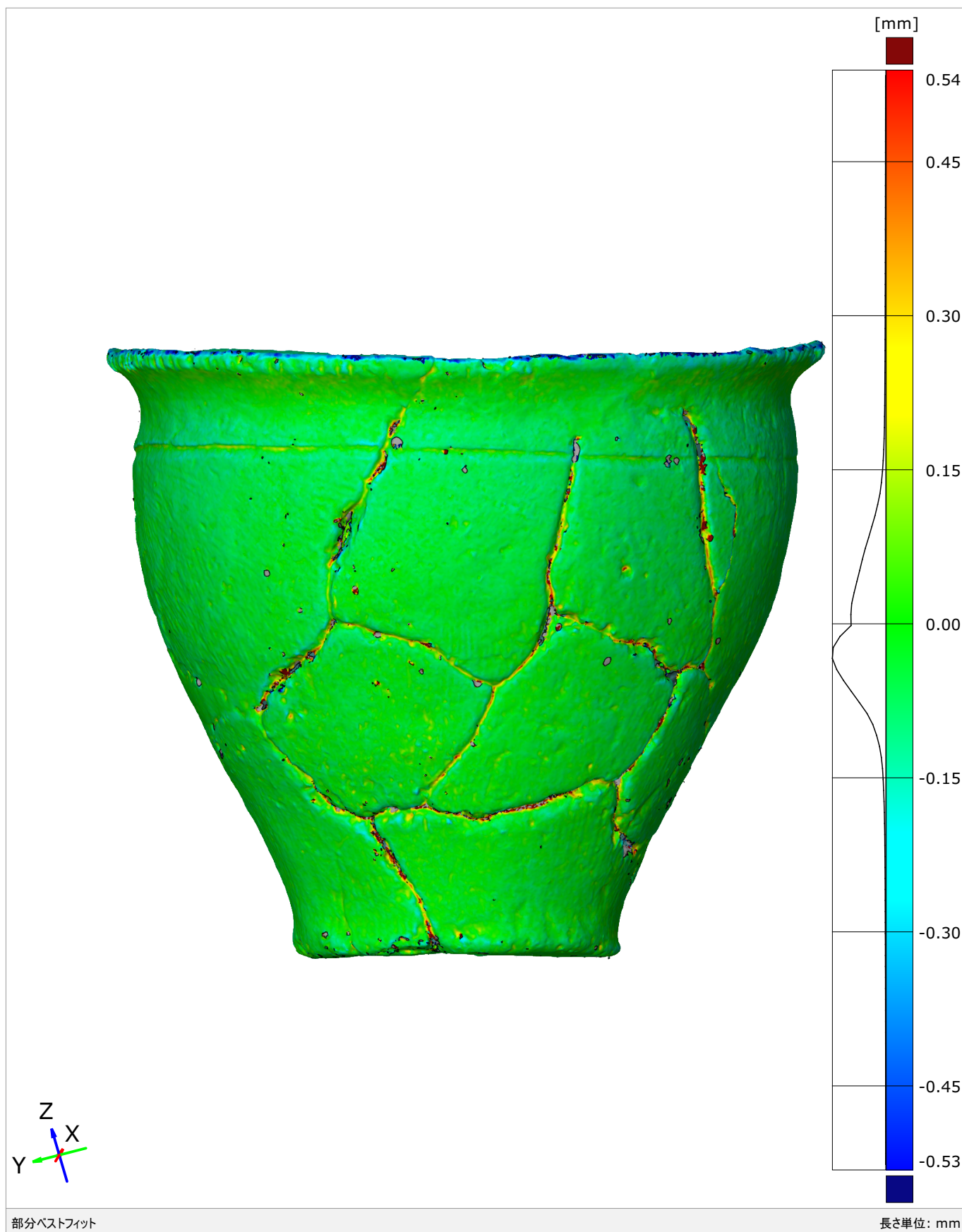

タイトルなし

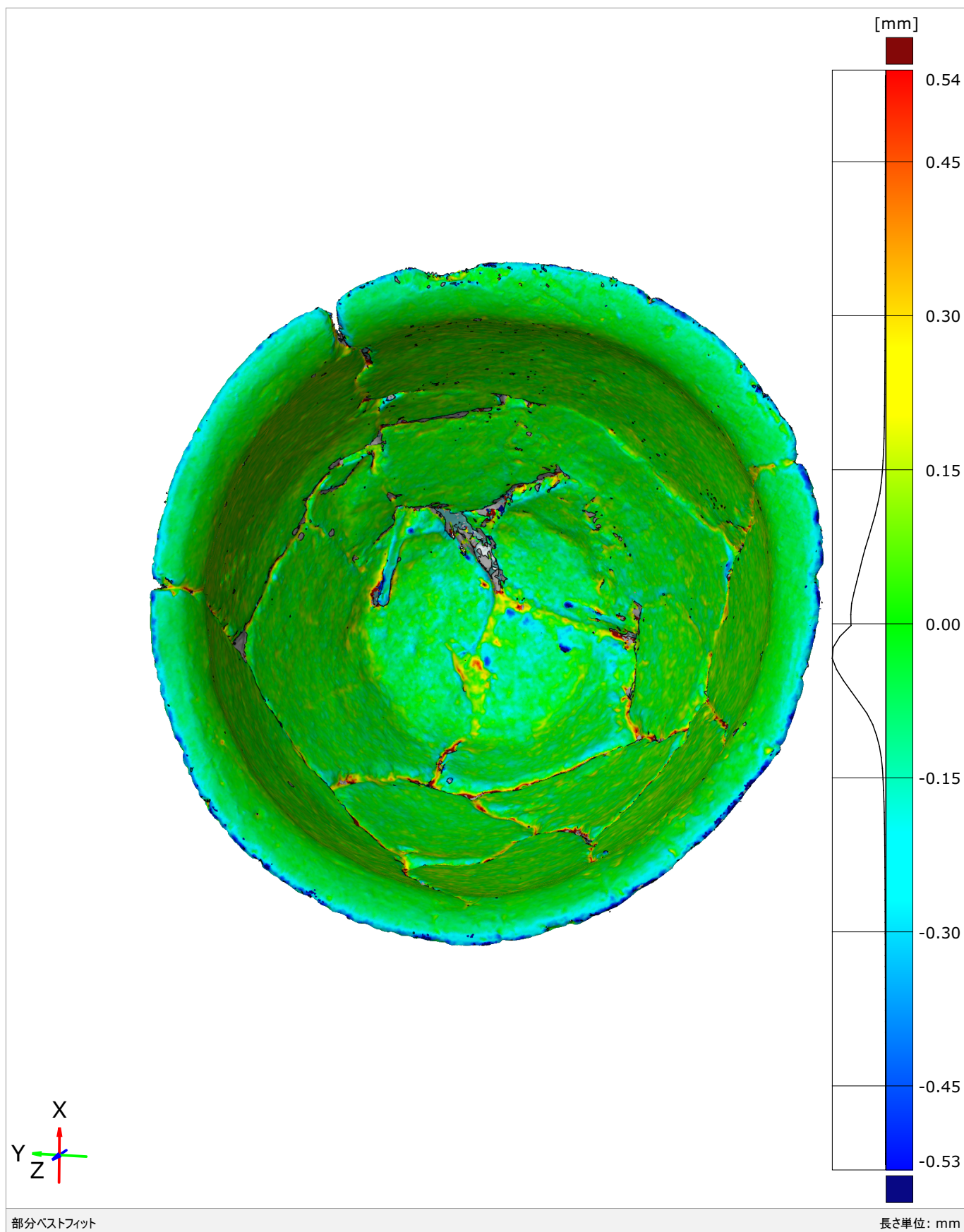

タイトルなし

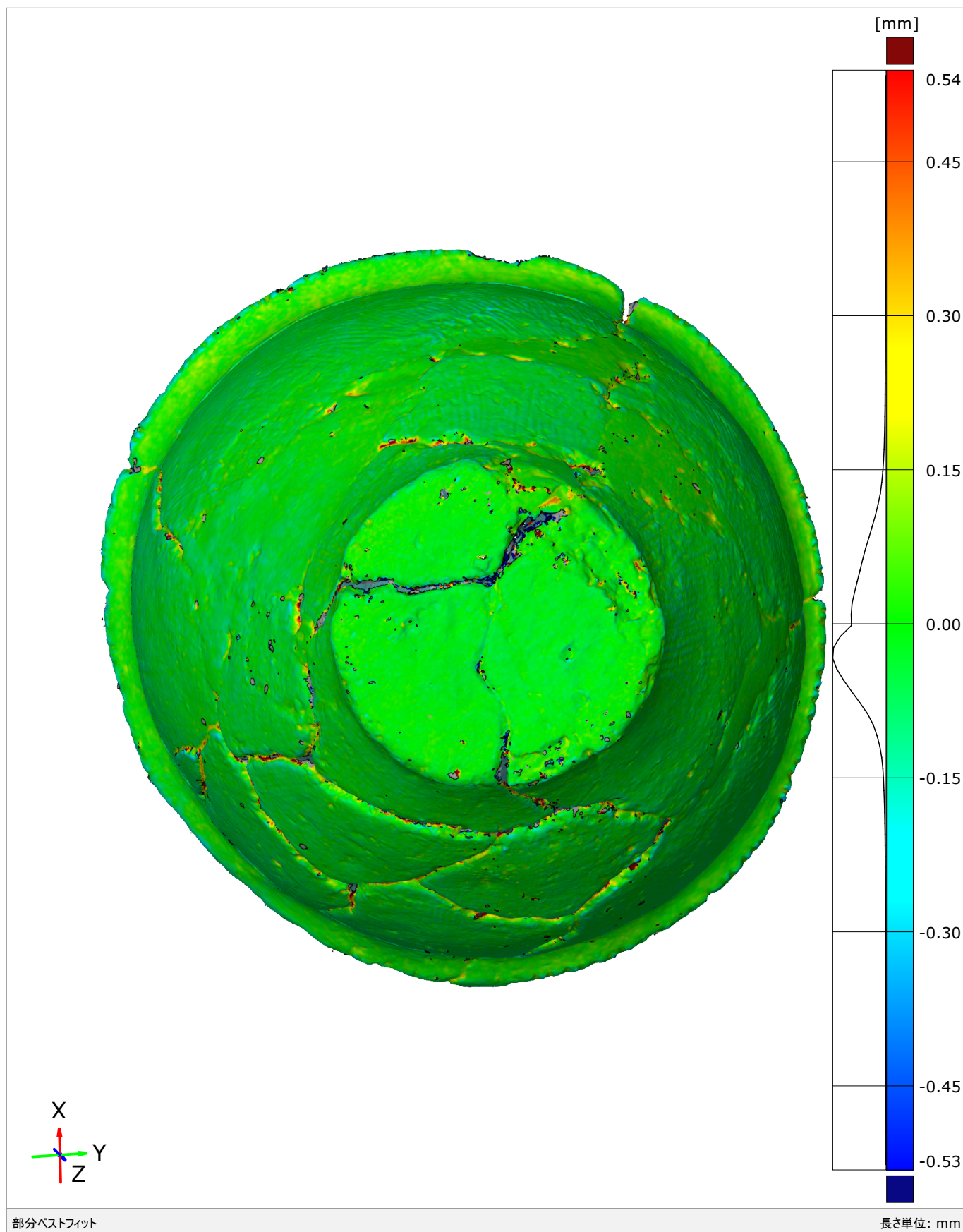

Supplement: S20 Fig — (PDF) [file pone.0270660.s020.pdf]

タイトルなし

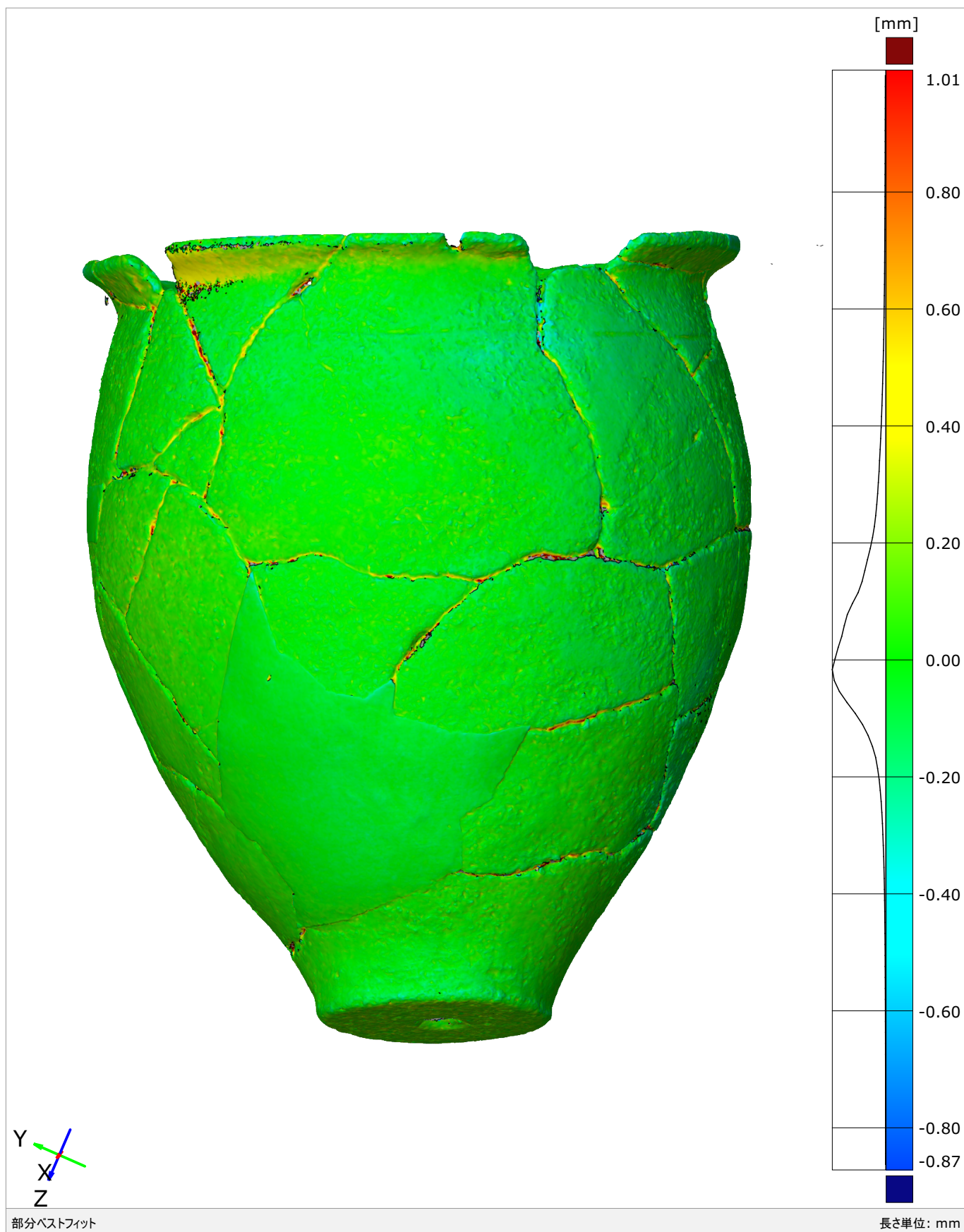

タイトルなし

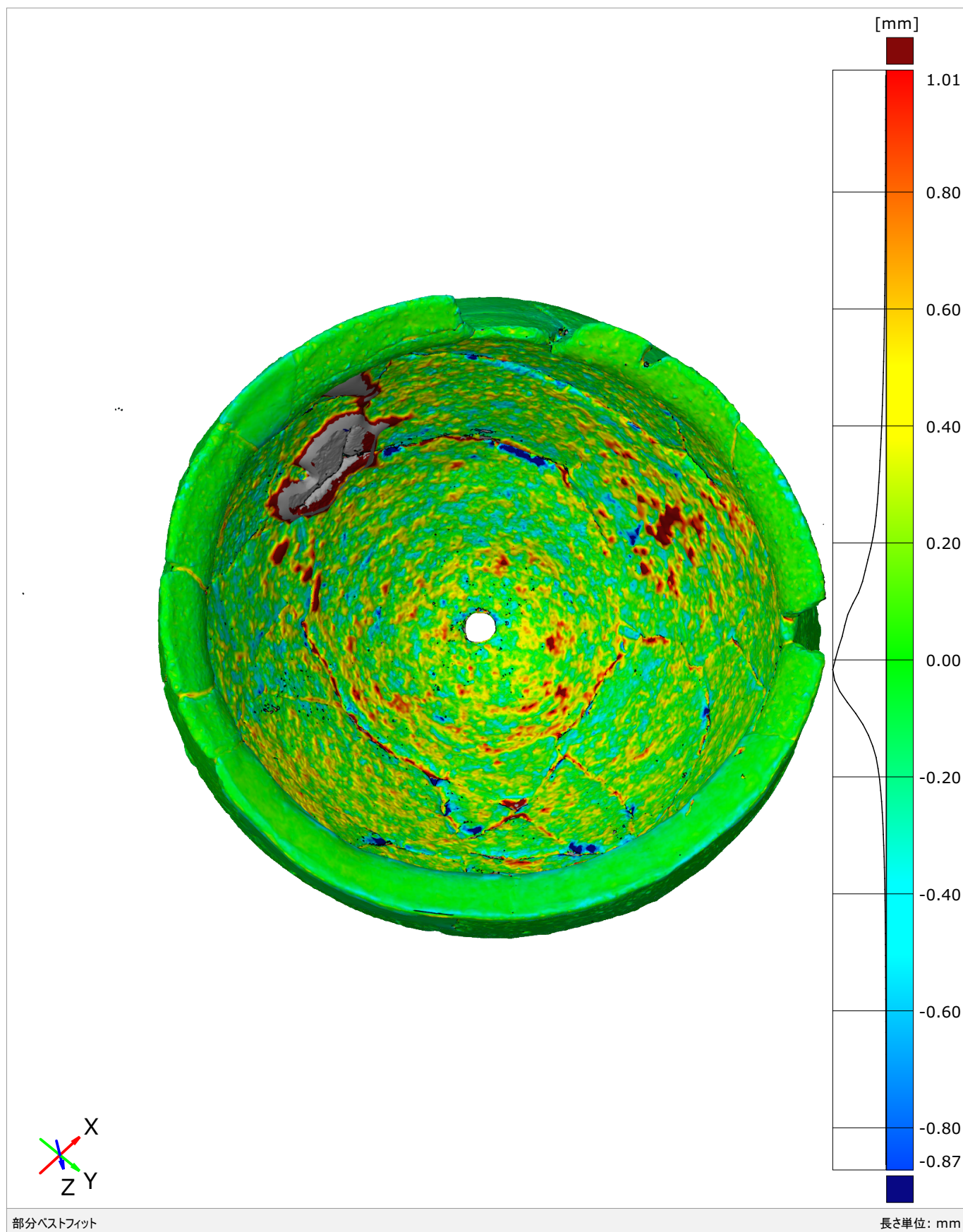

タイトルなし

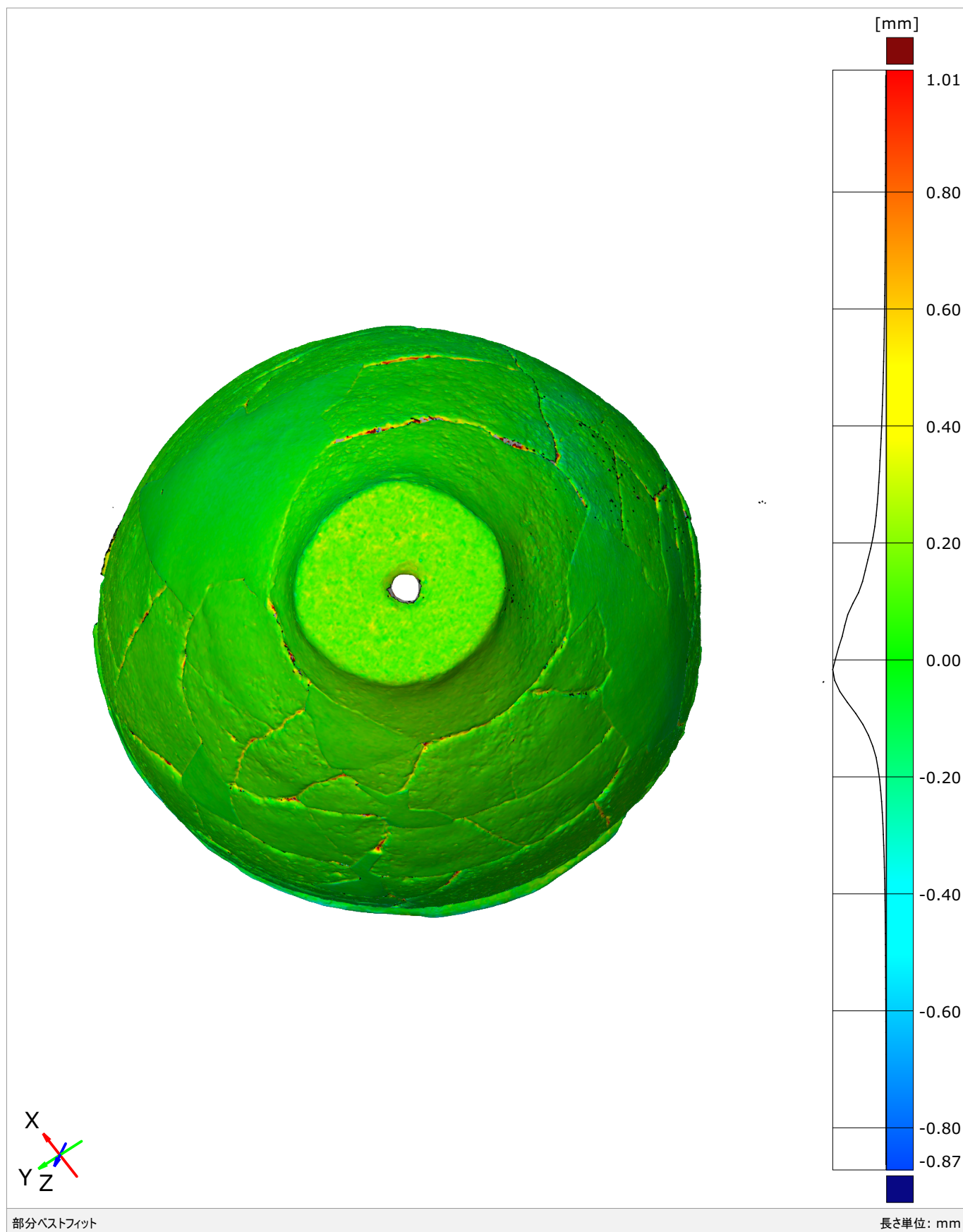

Supplement: S21 Fig — (PDF) [file pone.0270660.s021.pdf]

タイトルなし

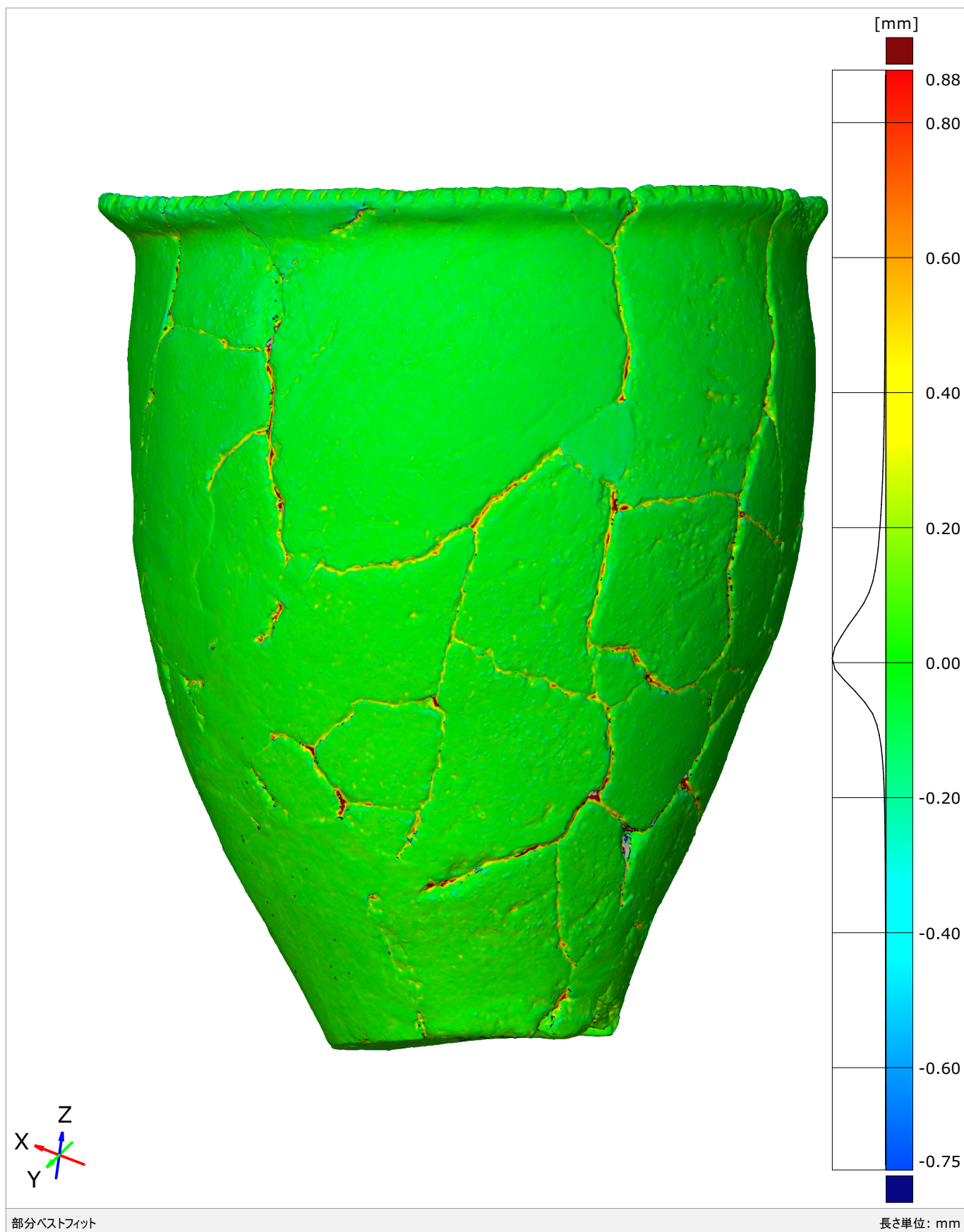

タイトルなし

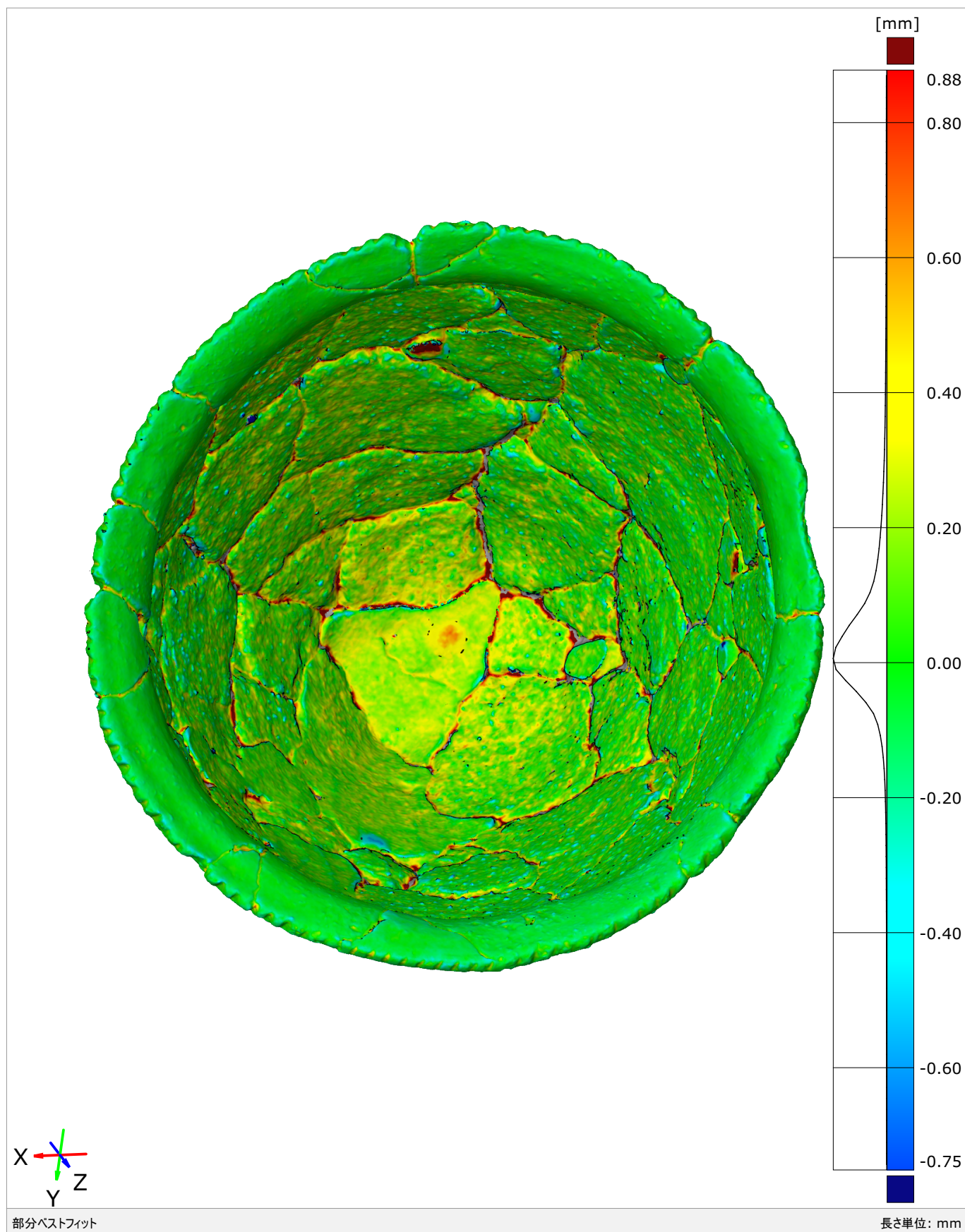

タイトルなし

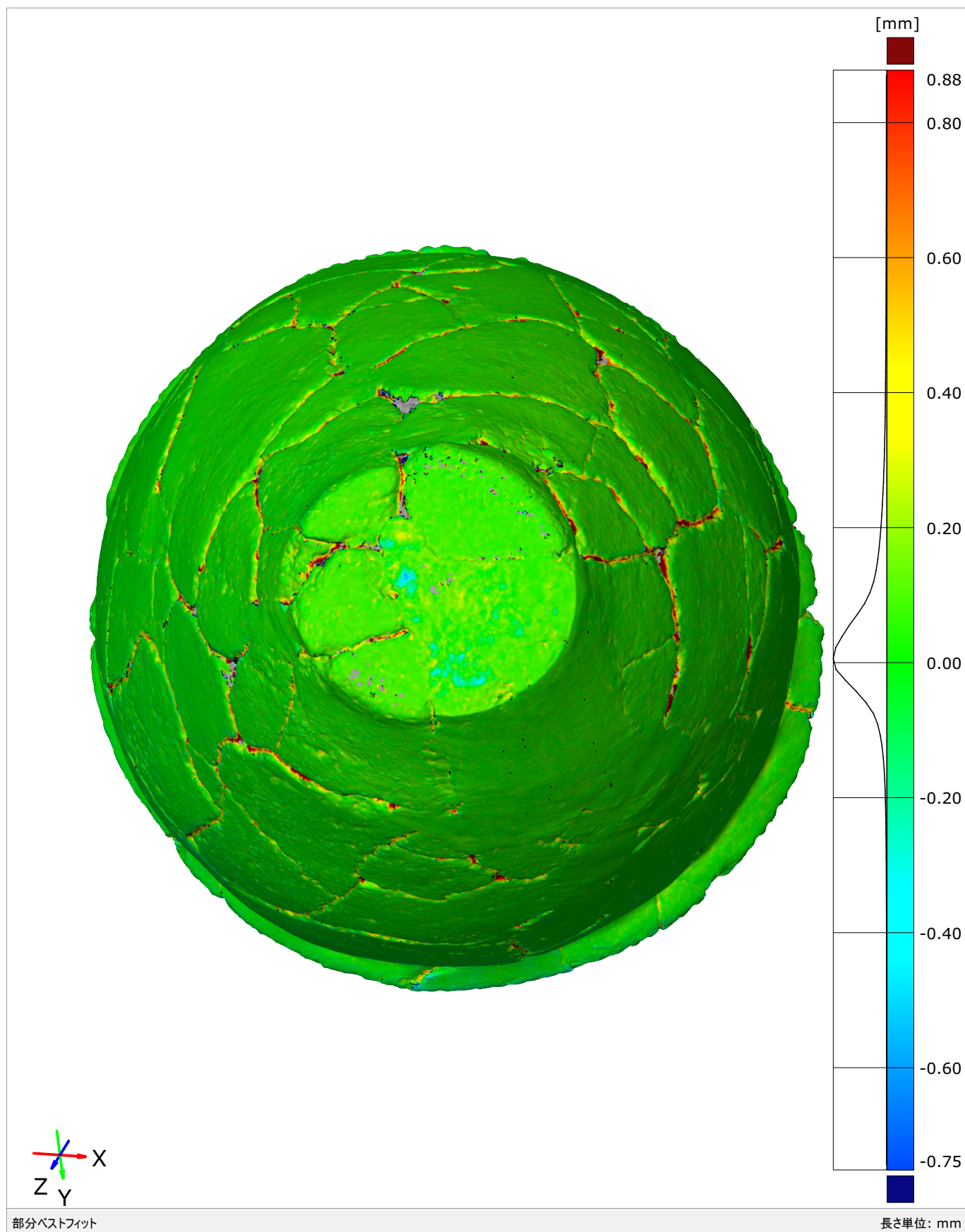

Supplement: S22 Fig — (PDF) [file pone.0270660.s022.pdf]

タイトルなし

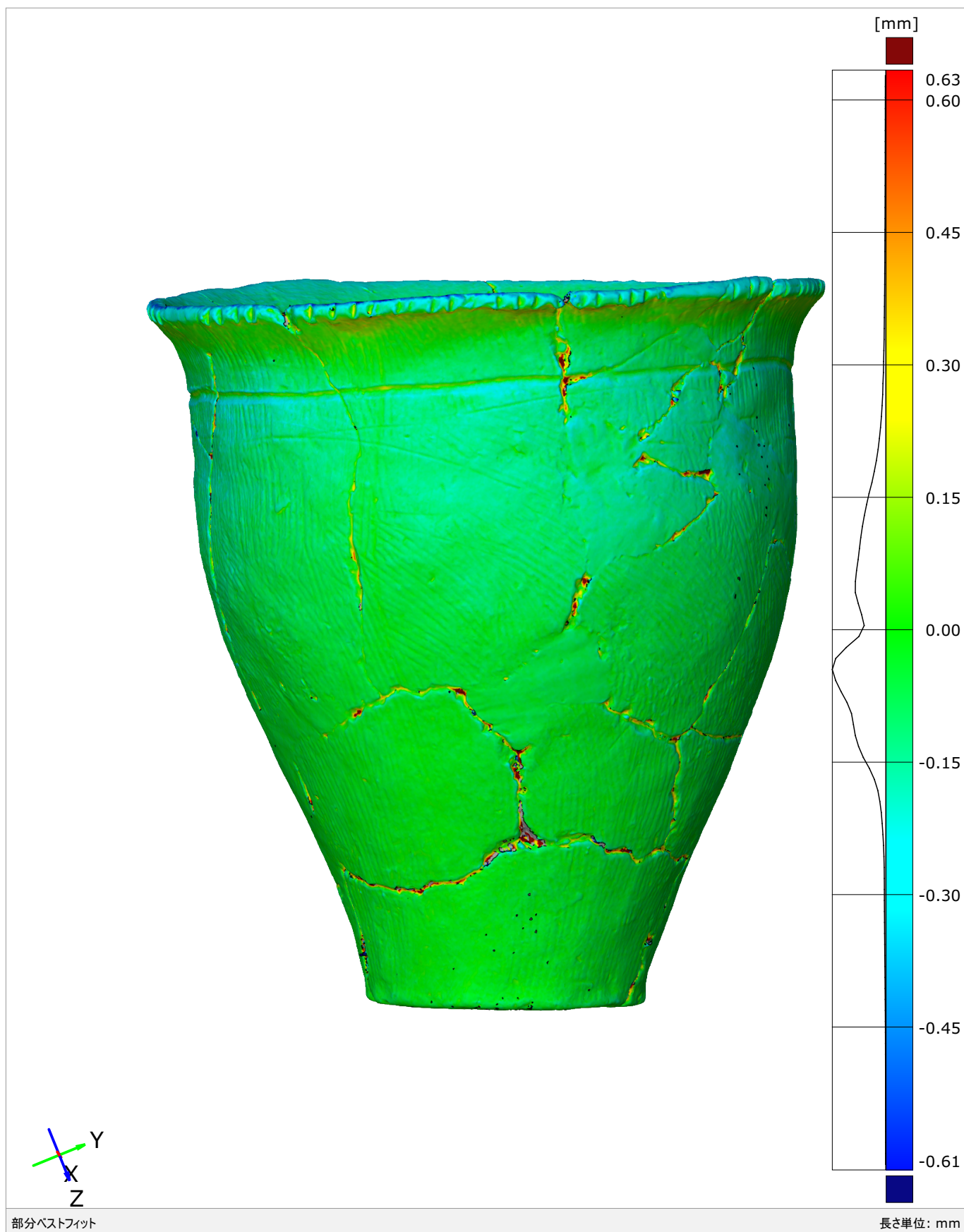

タイトルなし

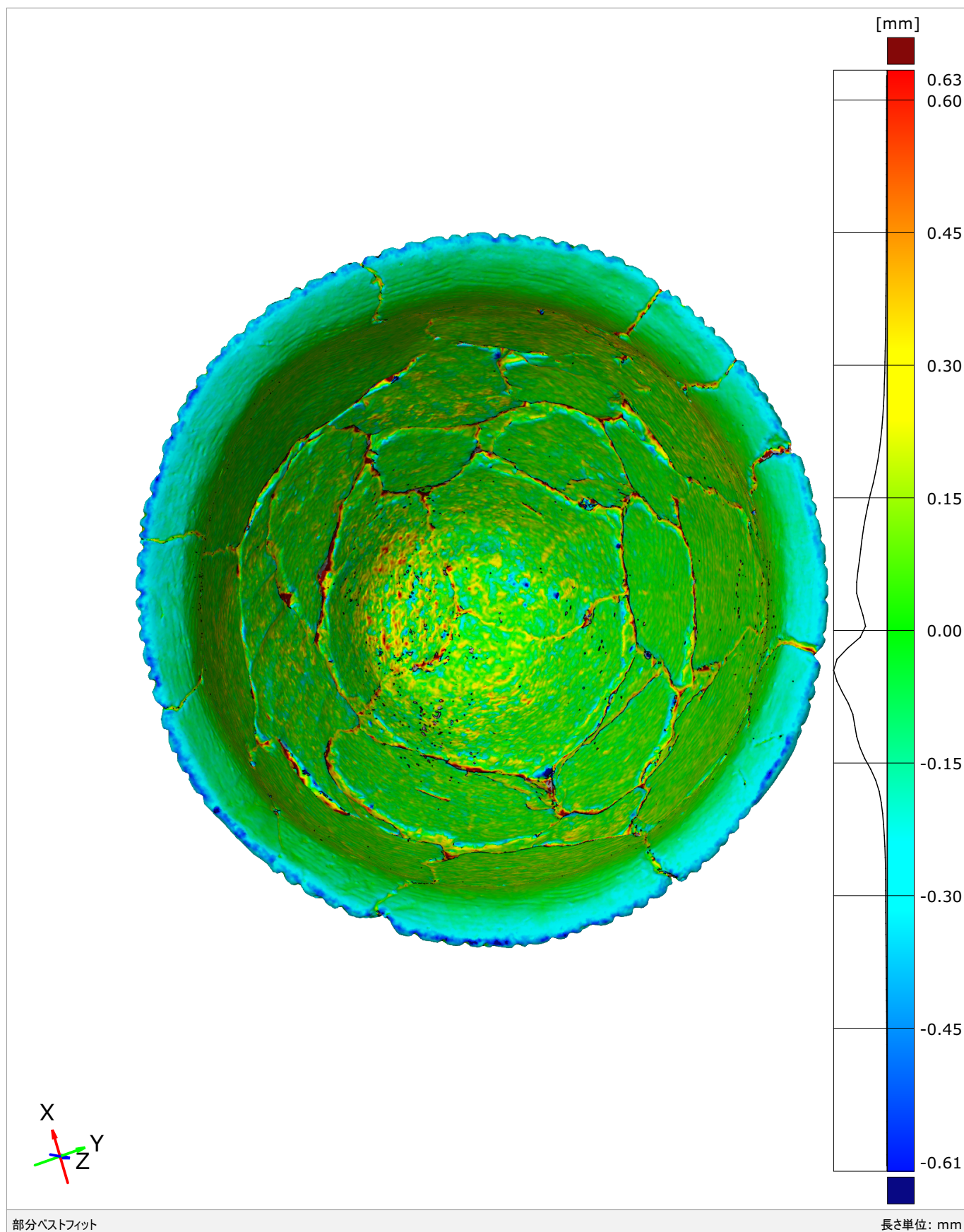

タイトルなし

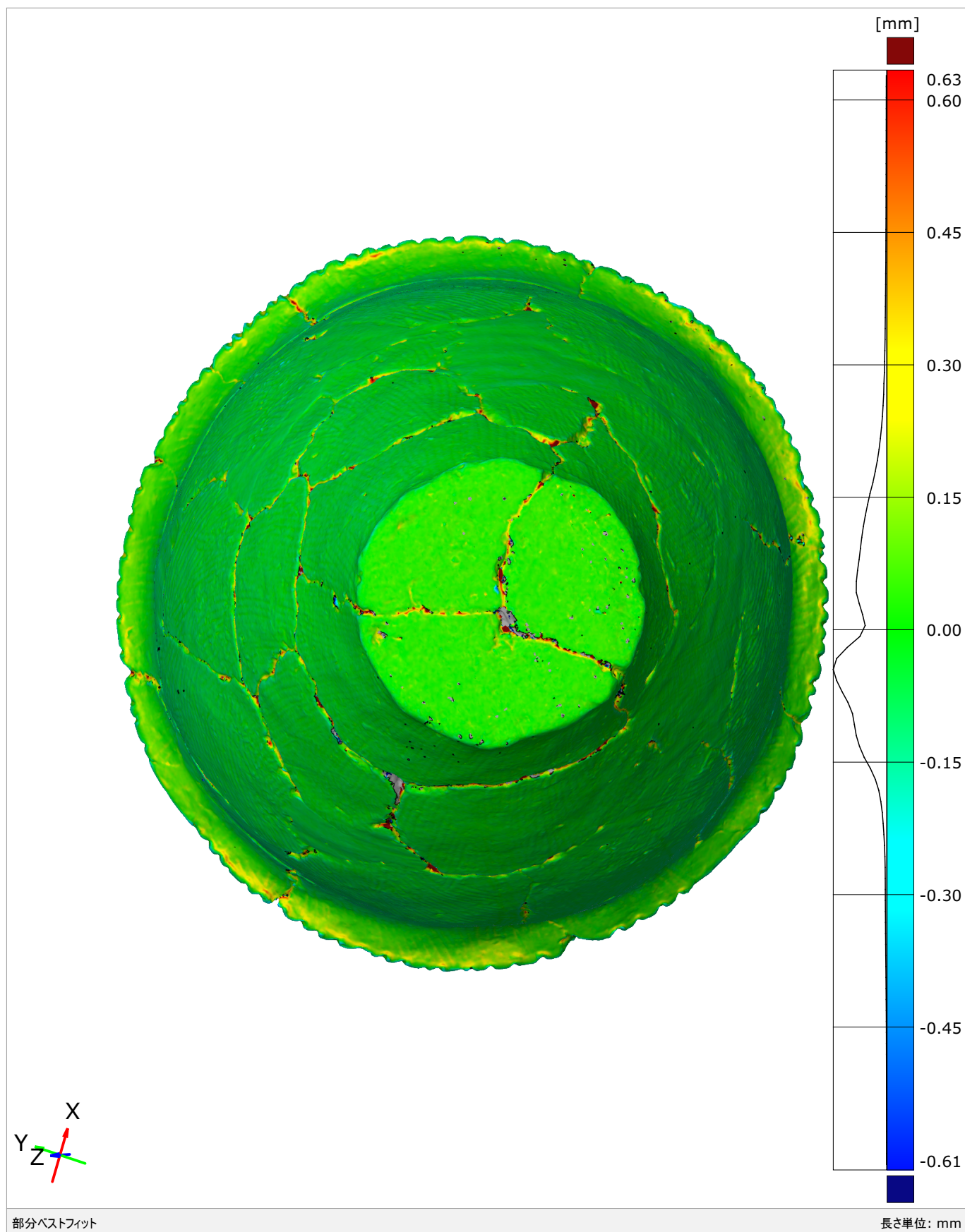

Supplement: S23 Fig — (PDF) [file pone.0270660.s023.pdf]

タイトルなし

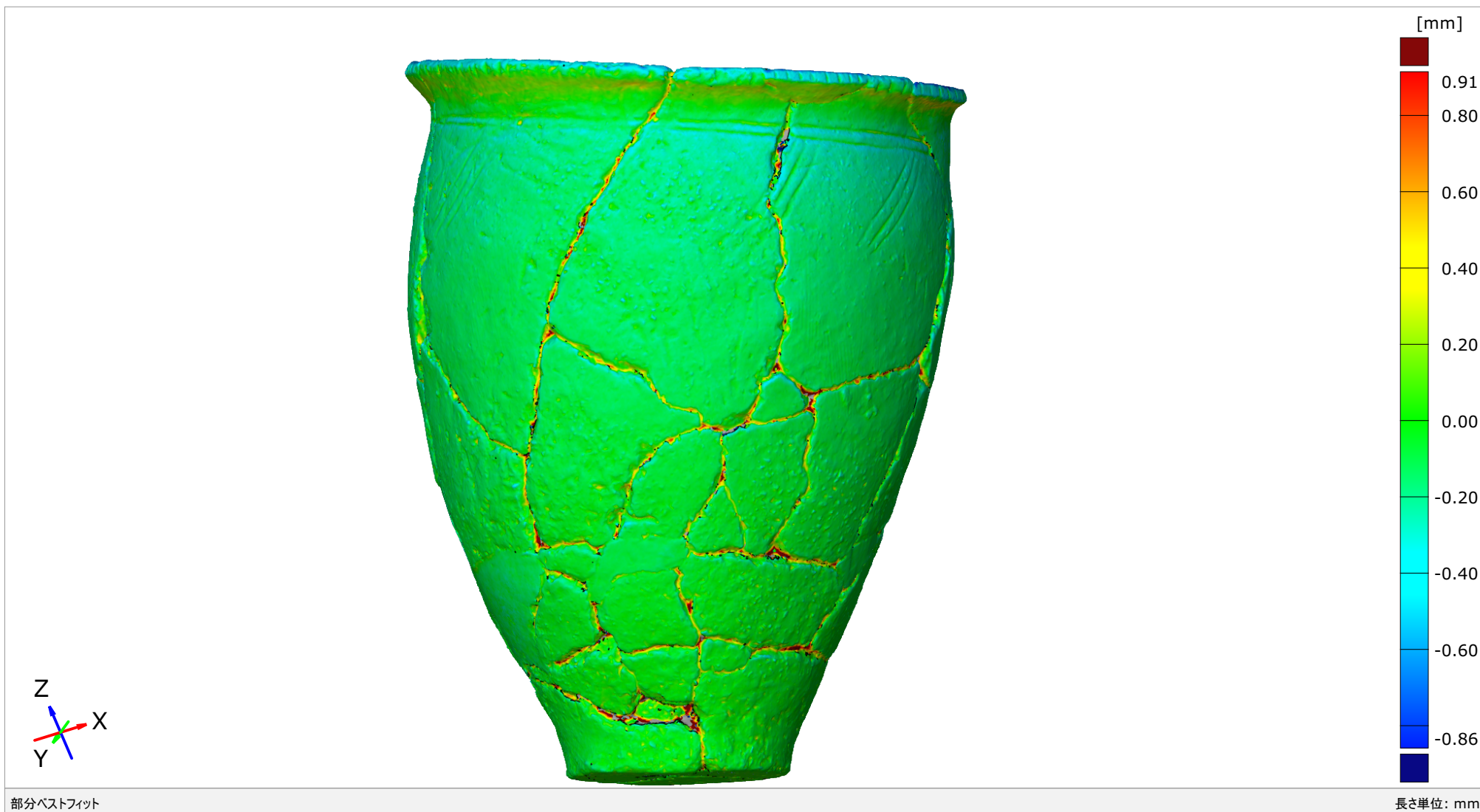

タイトルなし

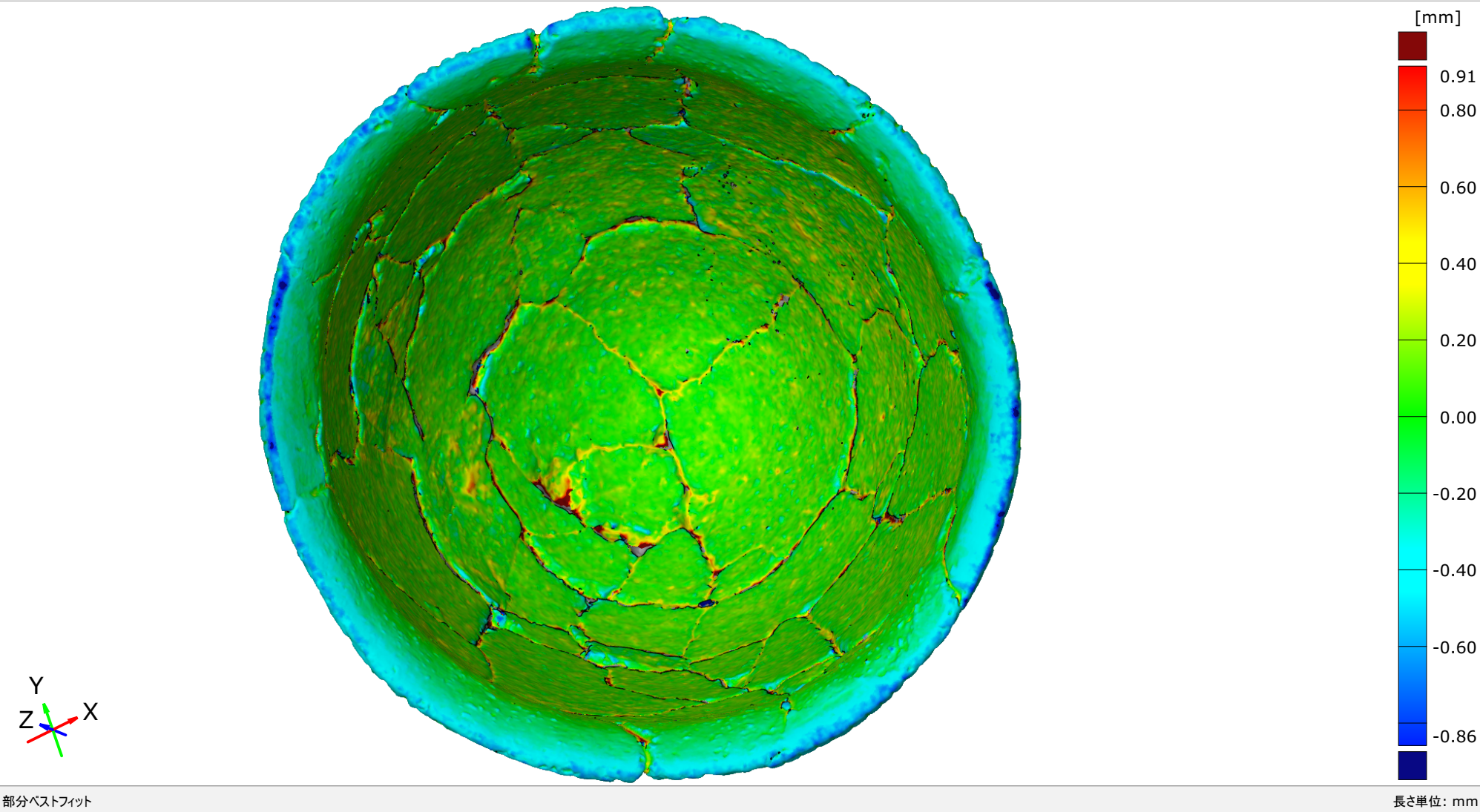

タイトルなし

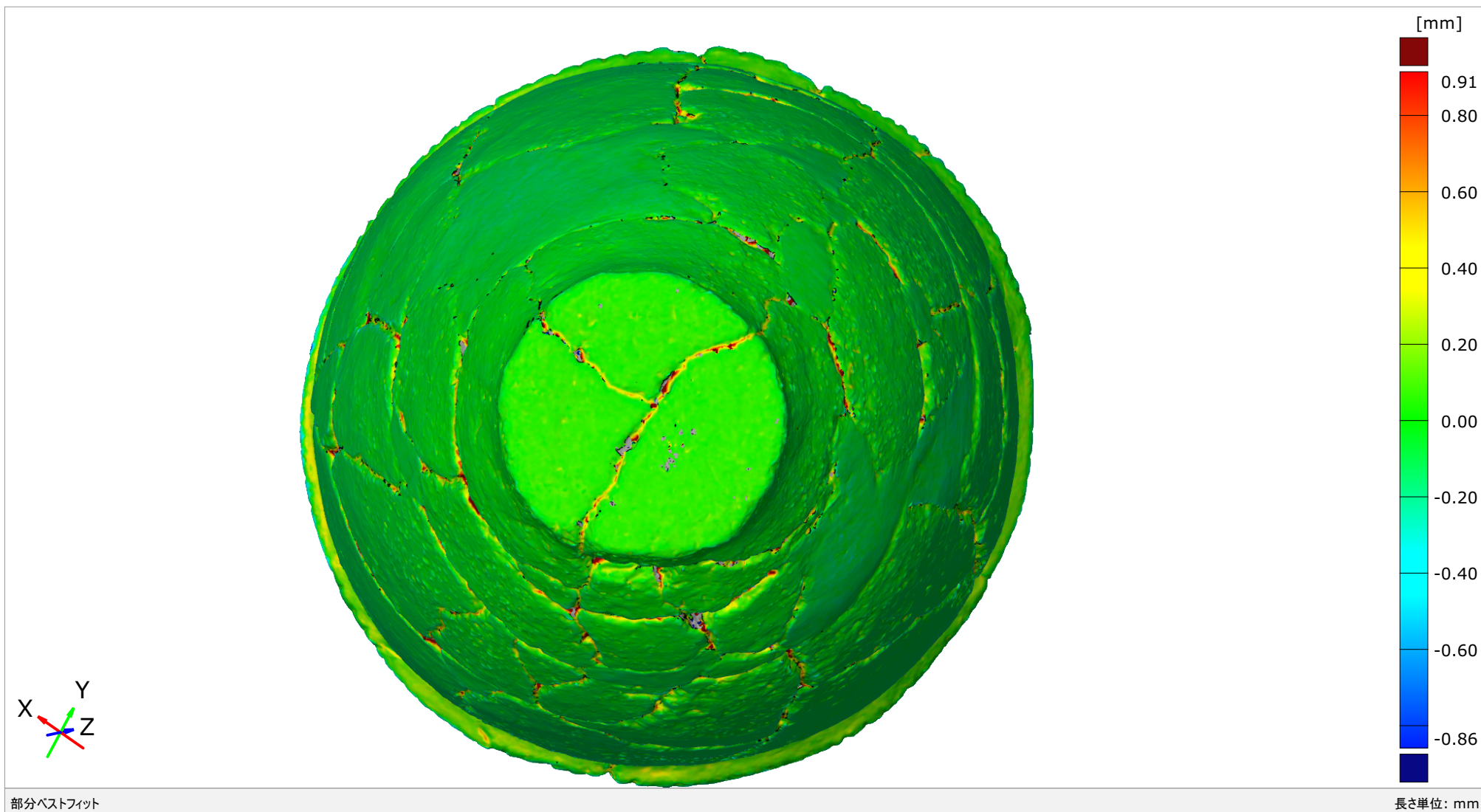

タイトルなし

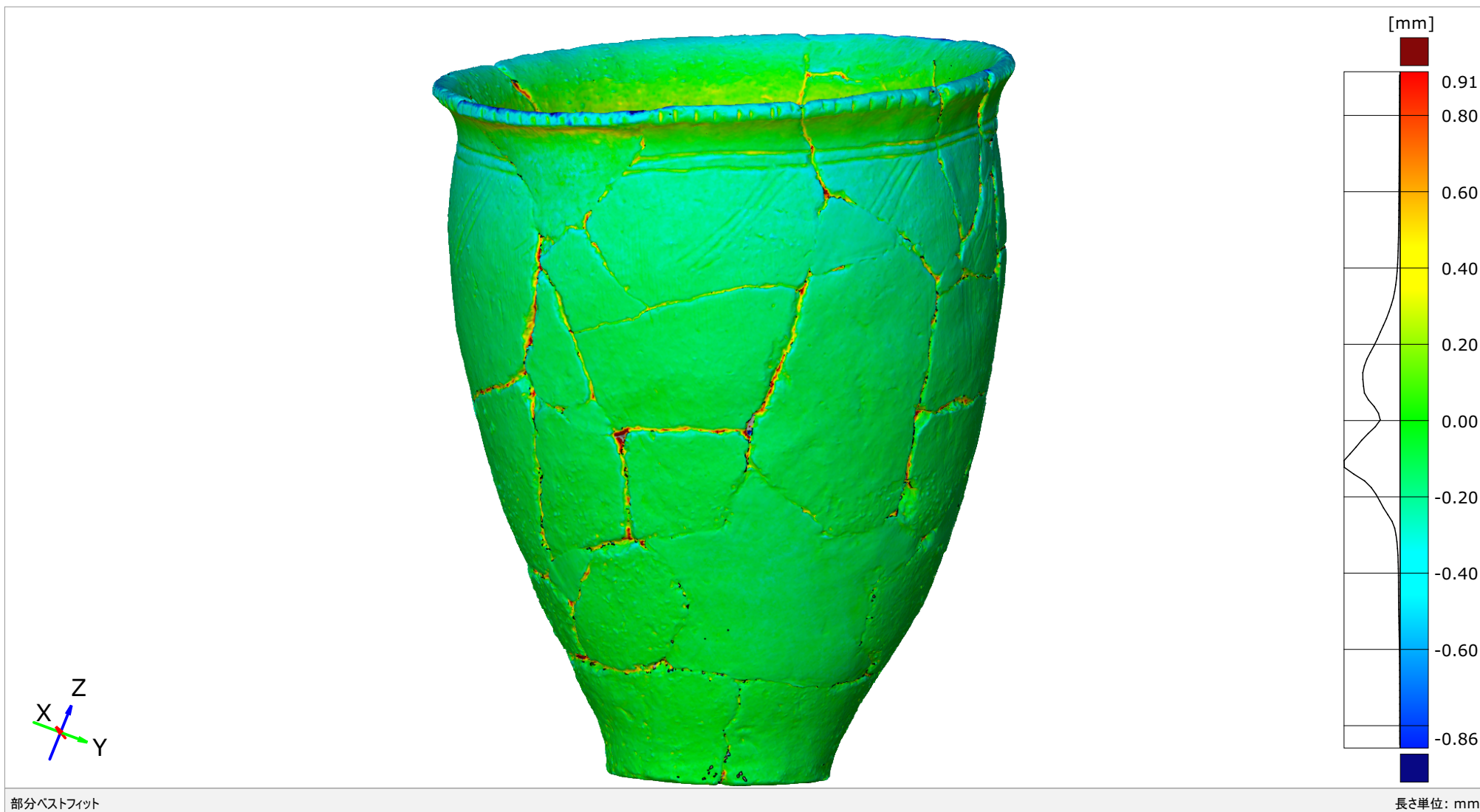

タイトルなし

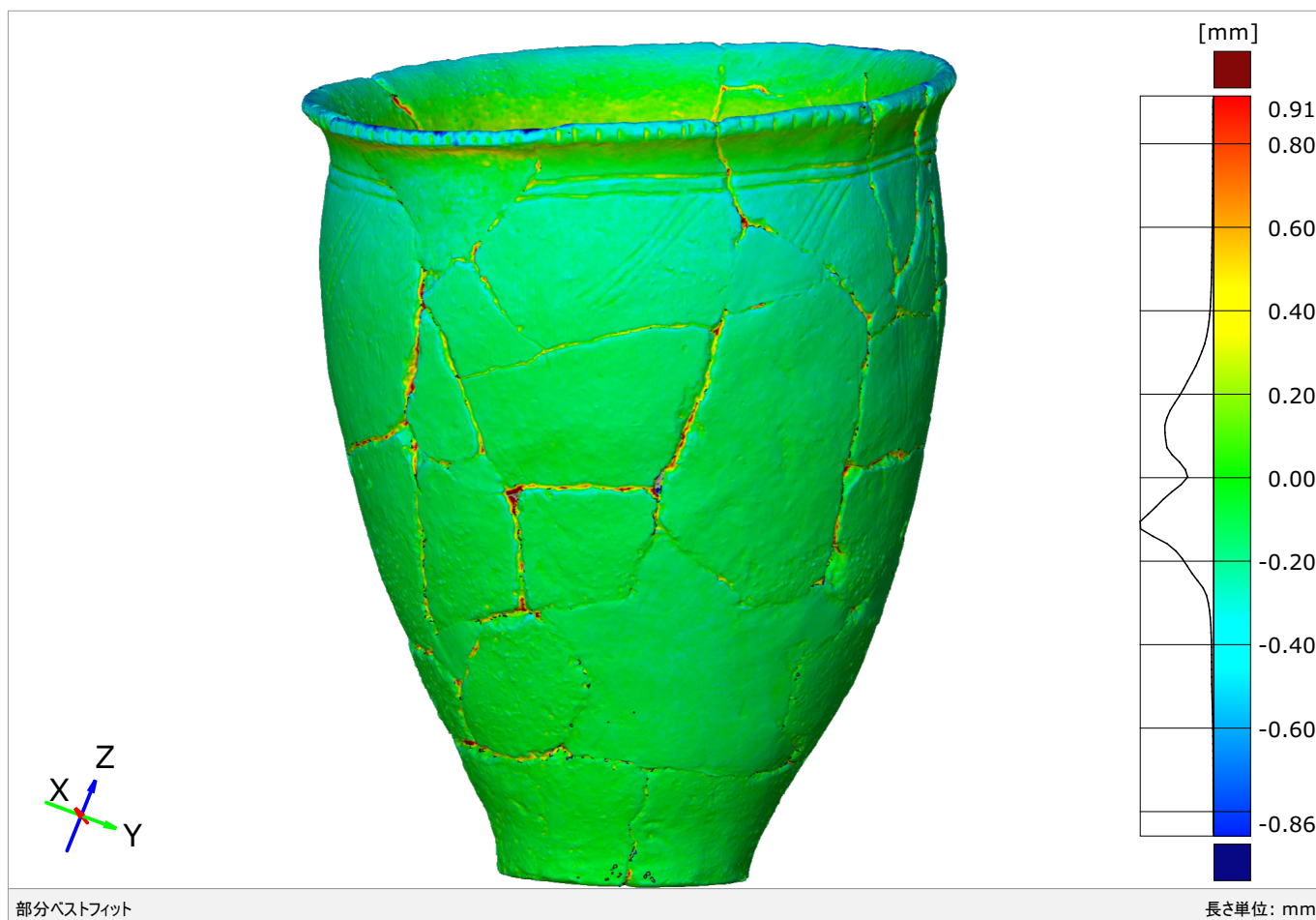

検査担当者:

会社名:

部署:

場所:

日付: 2021/01/17

プロジェクト:

部品:

部品番号:

バージョン:

ロットナンバー.:

システム:

Supplement: S24 Fig — (PDF) [file pone.0270660.s024.pdf]

タイトルなし

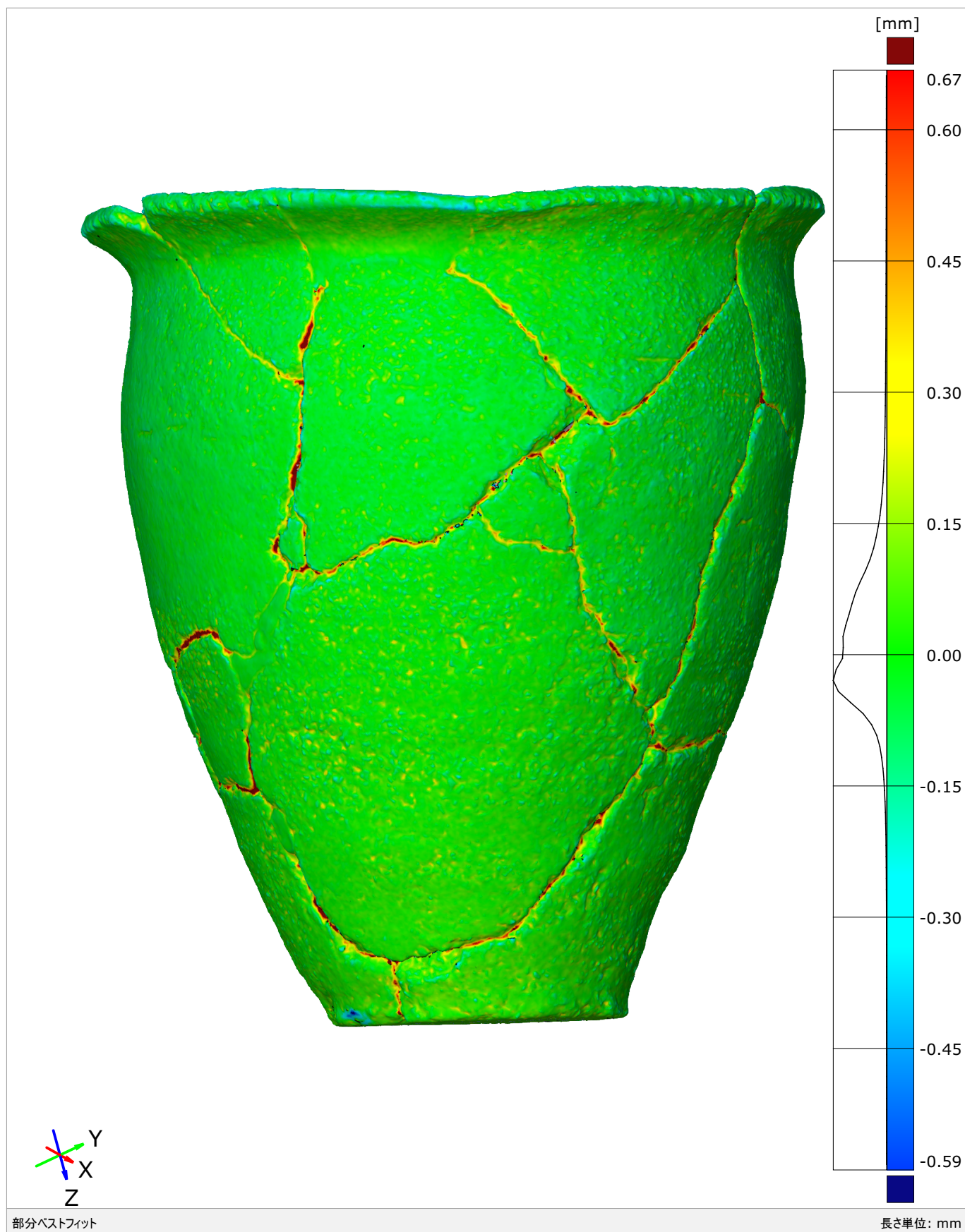

タイトルなし

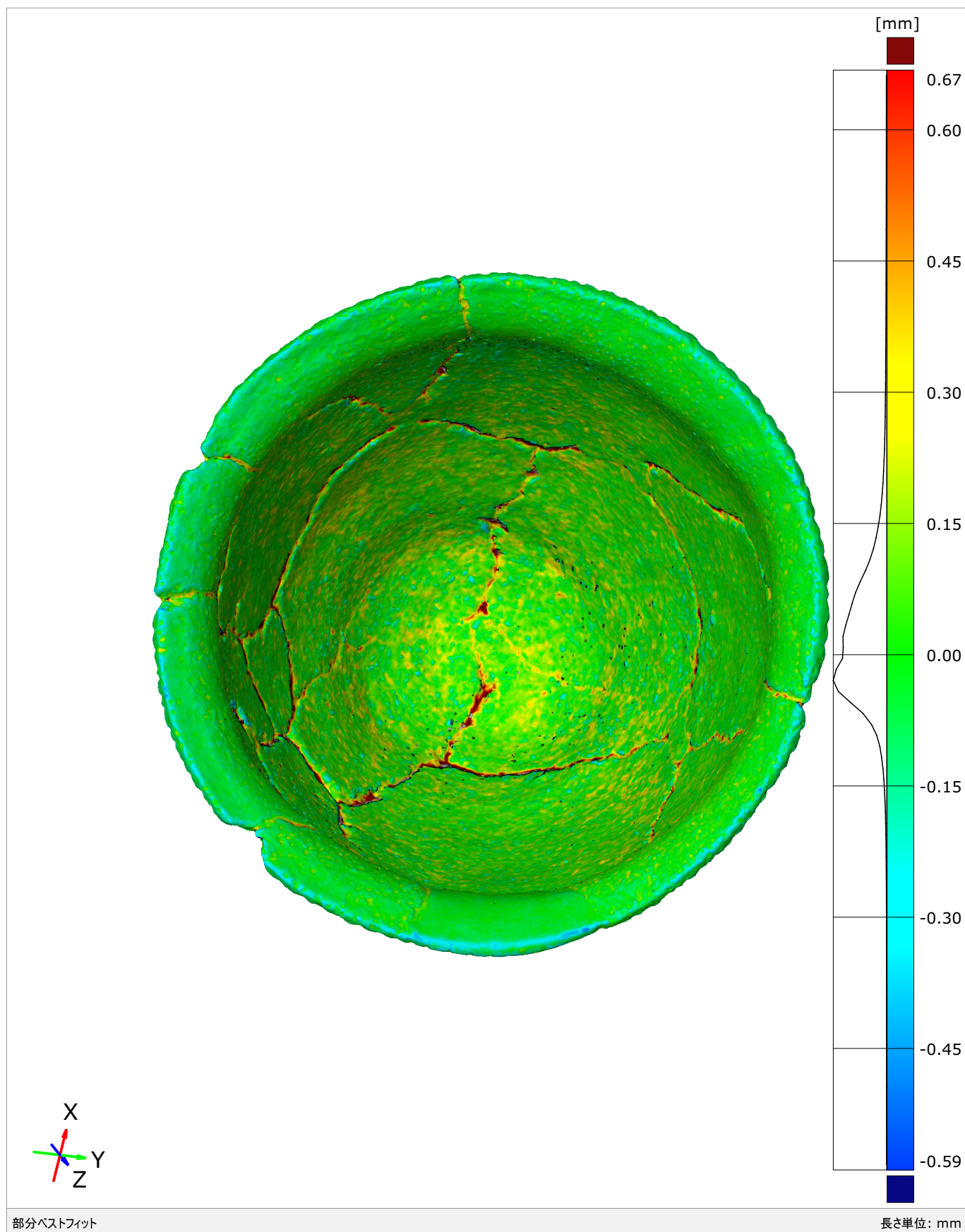

タイトルなし

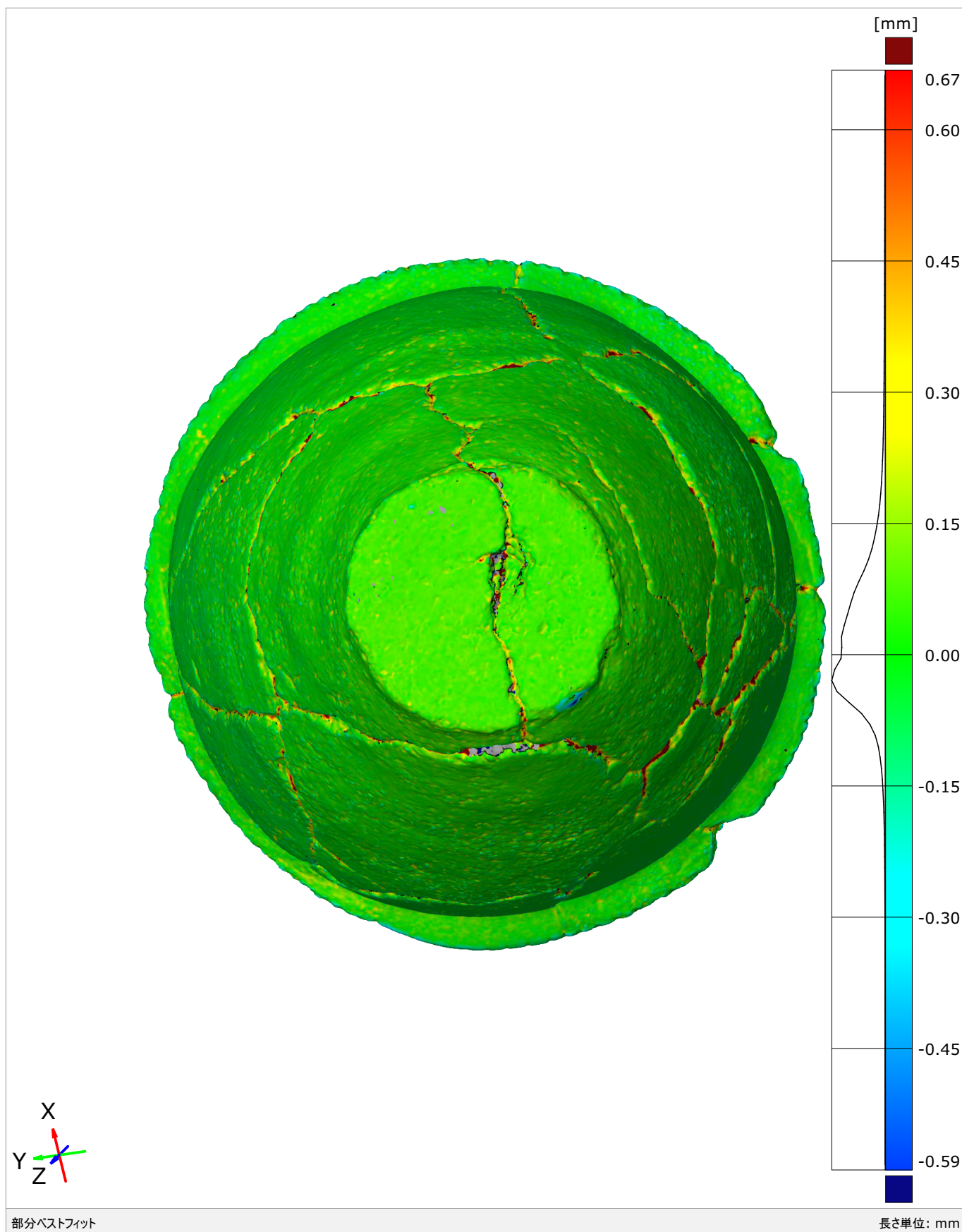

Supplement: S25 Fig — (PDF) [file pone.0270660.s025.pdf]

タイトルなし

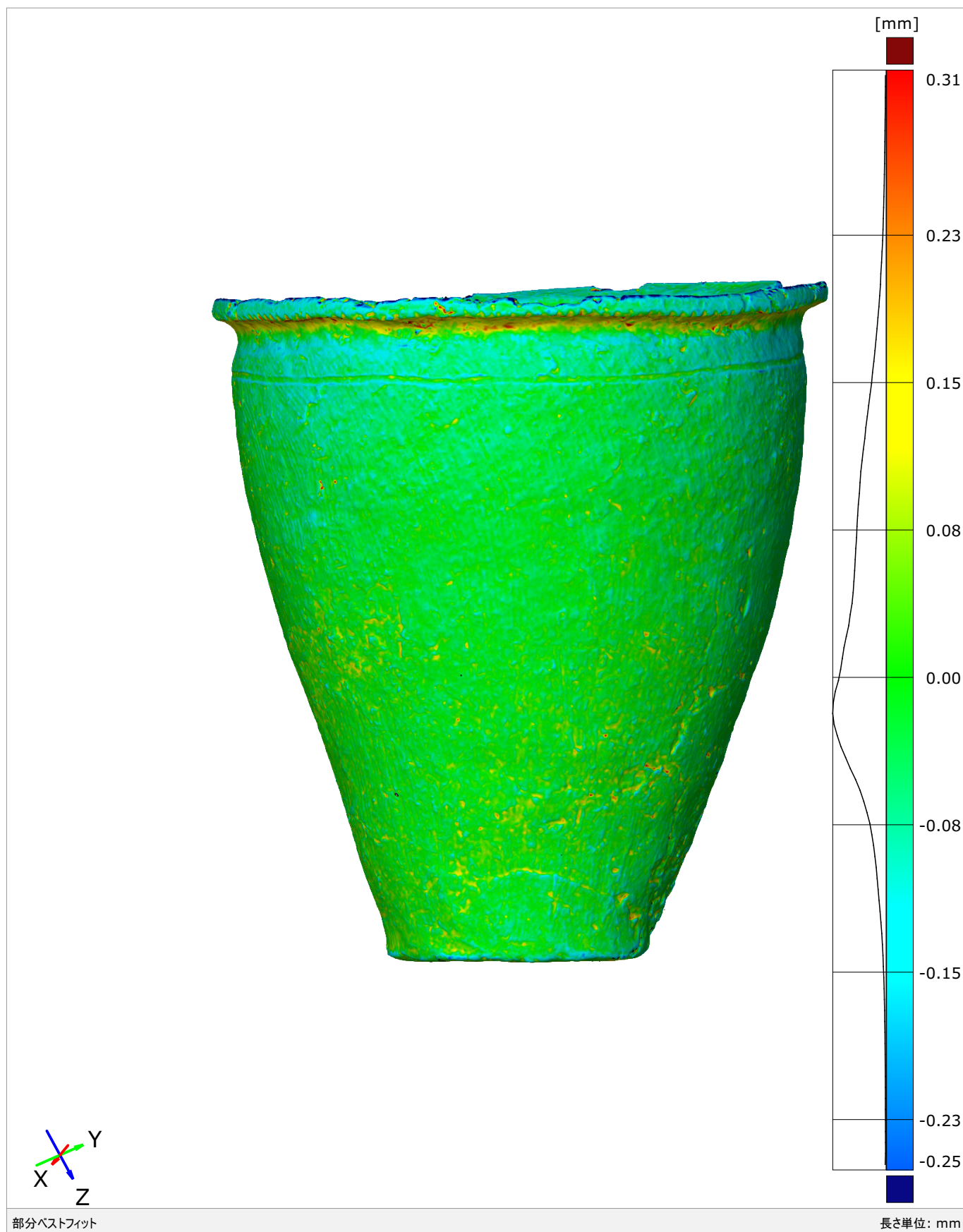

タイトルなし

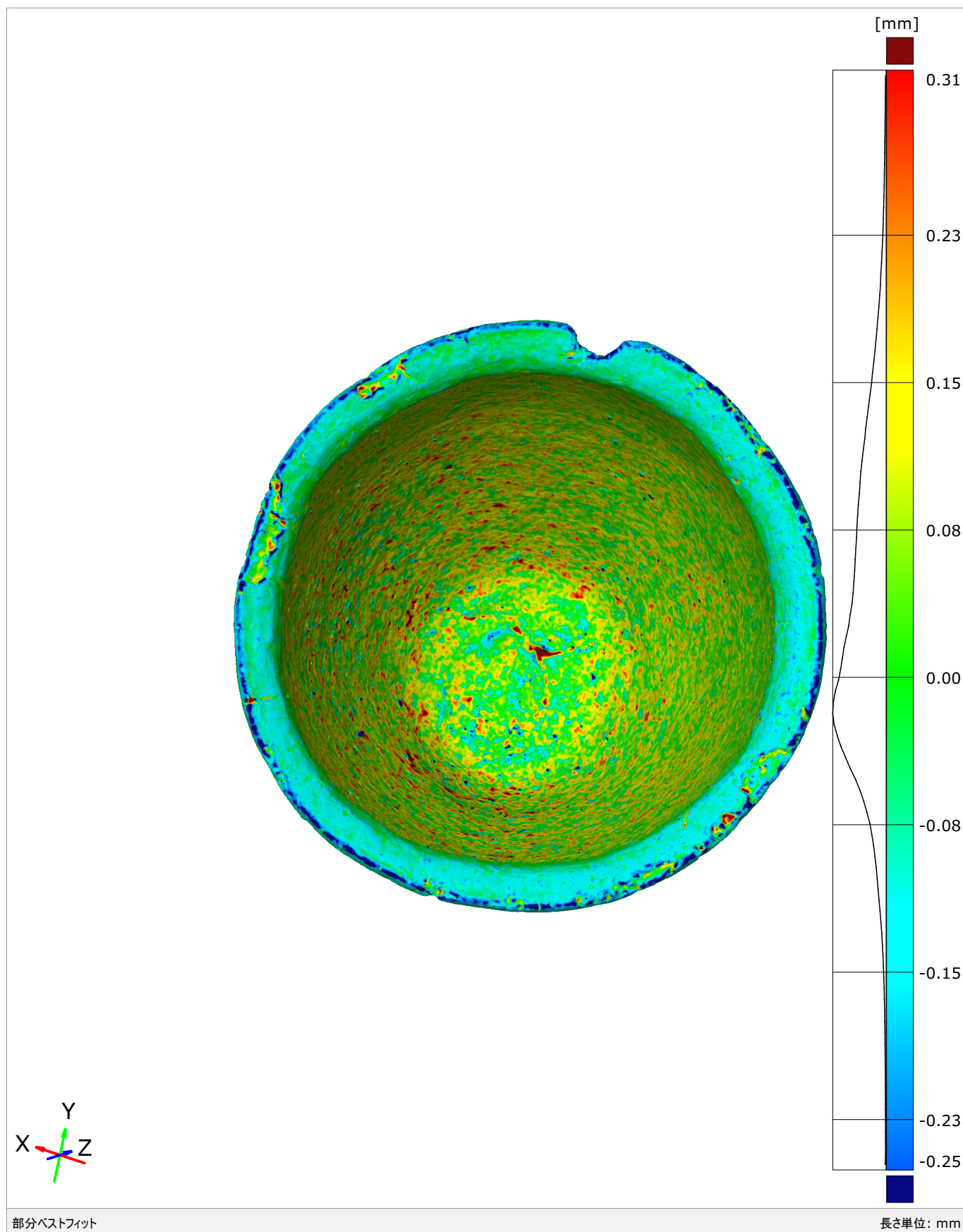

タイトルなし

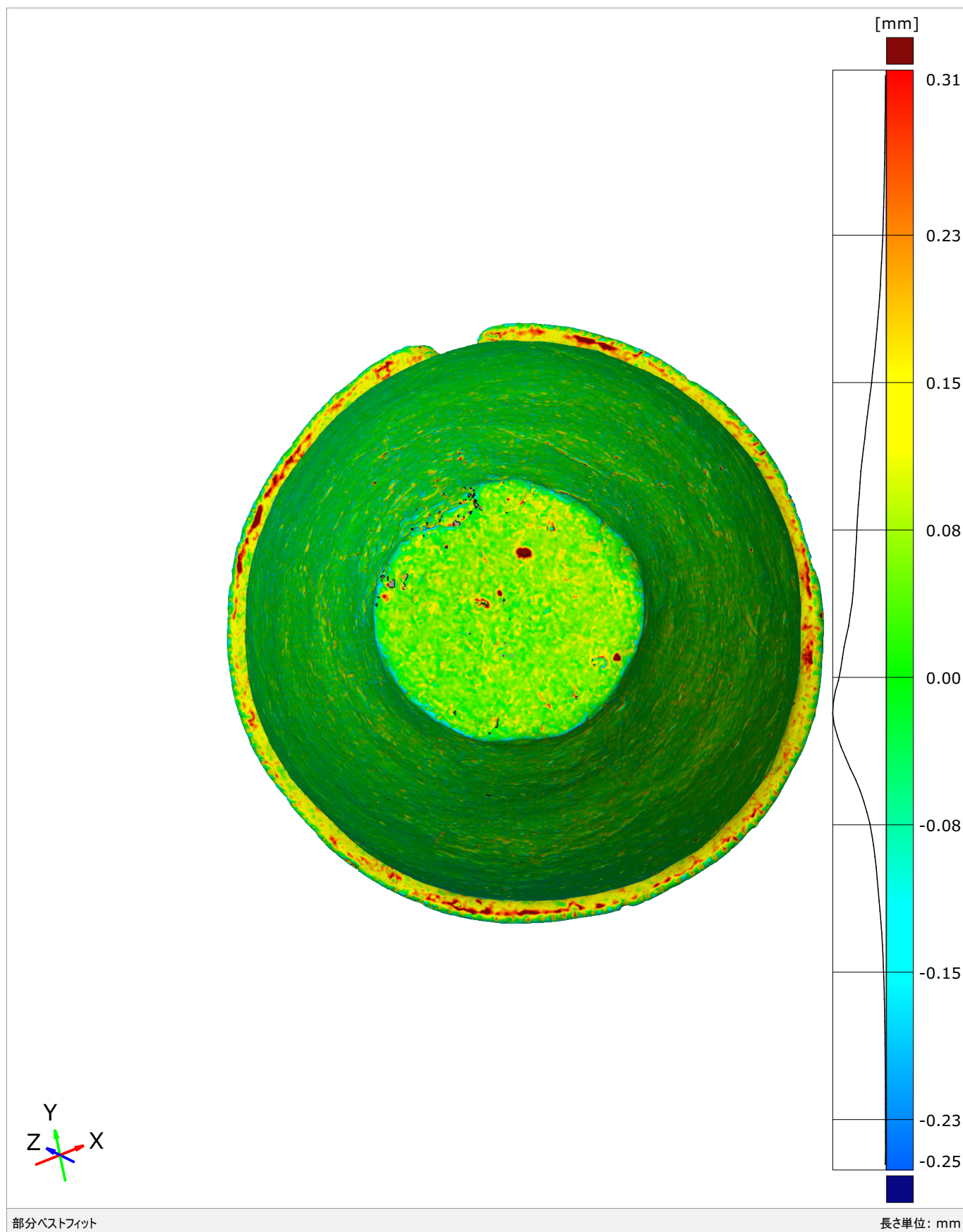

Supplement: S26 Fig — (PDF) [file pone.0270660.s026.pdf]

タイトルなし

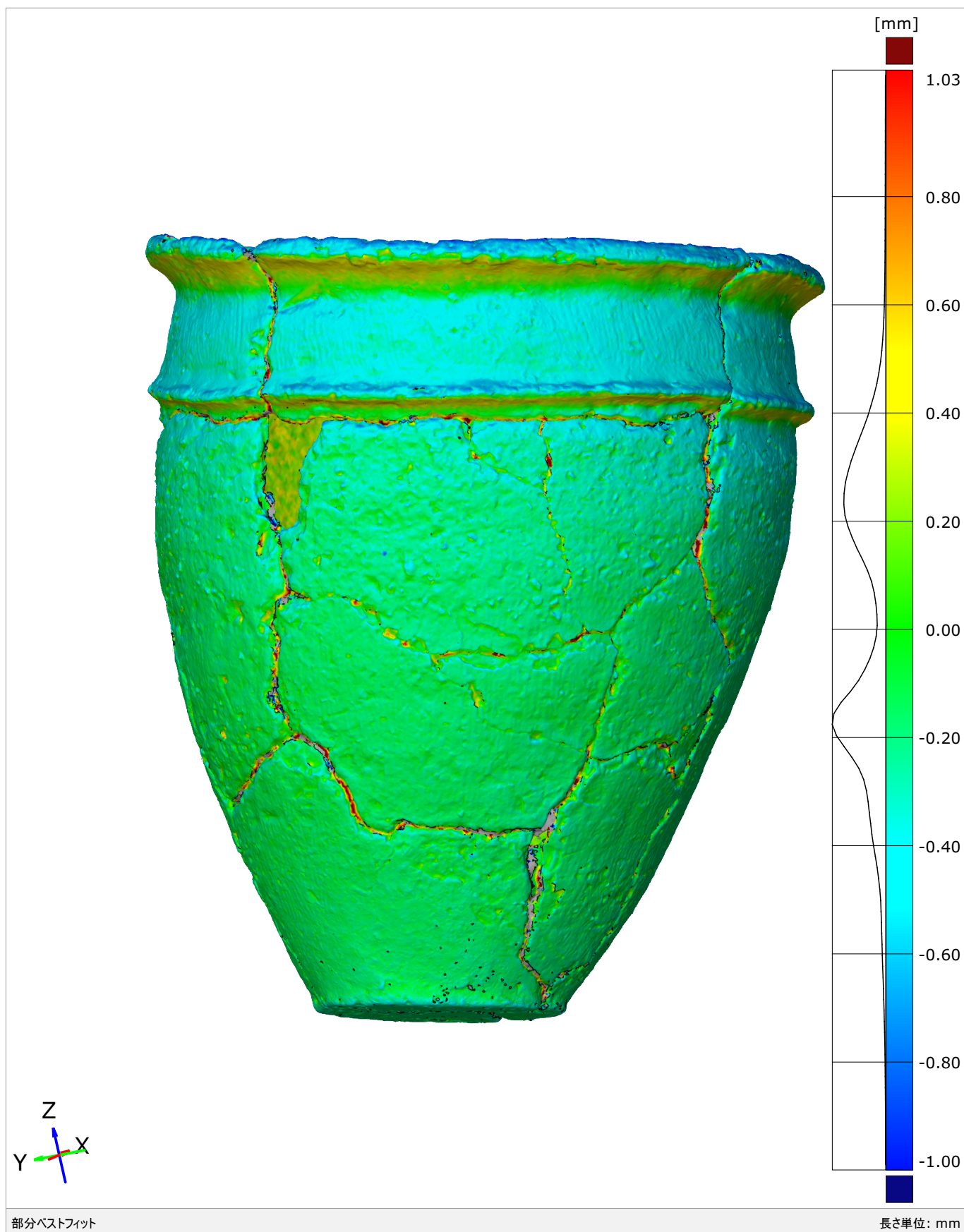

タイトルなし

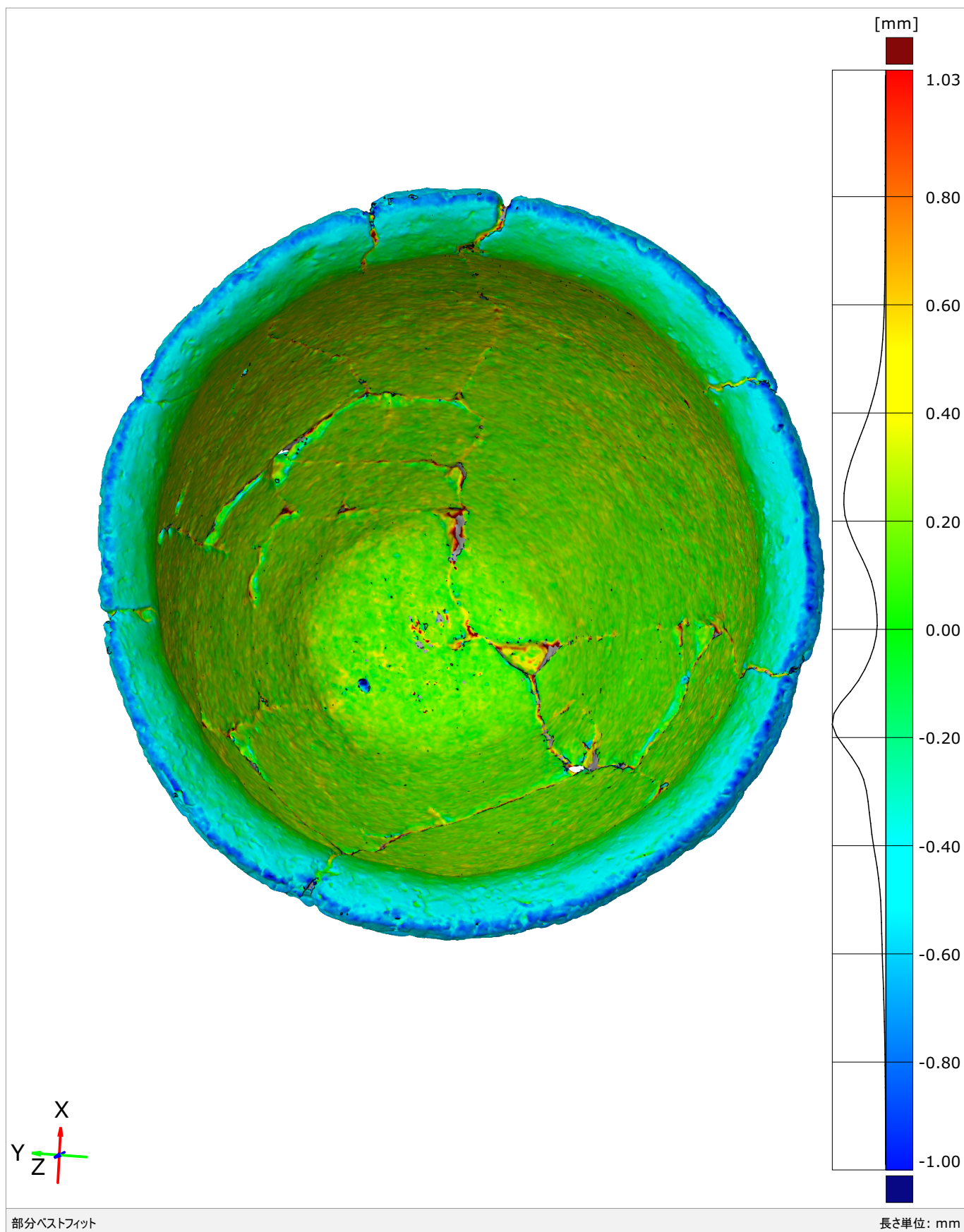

タイトルなし

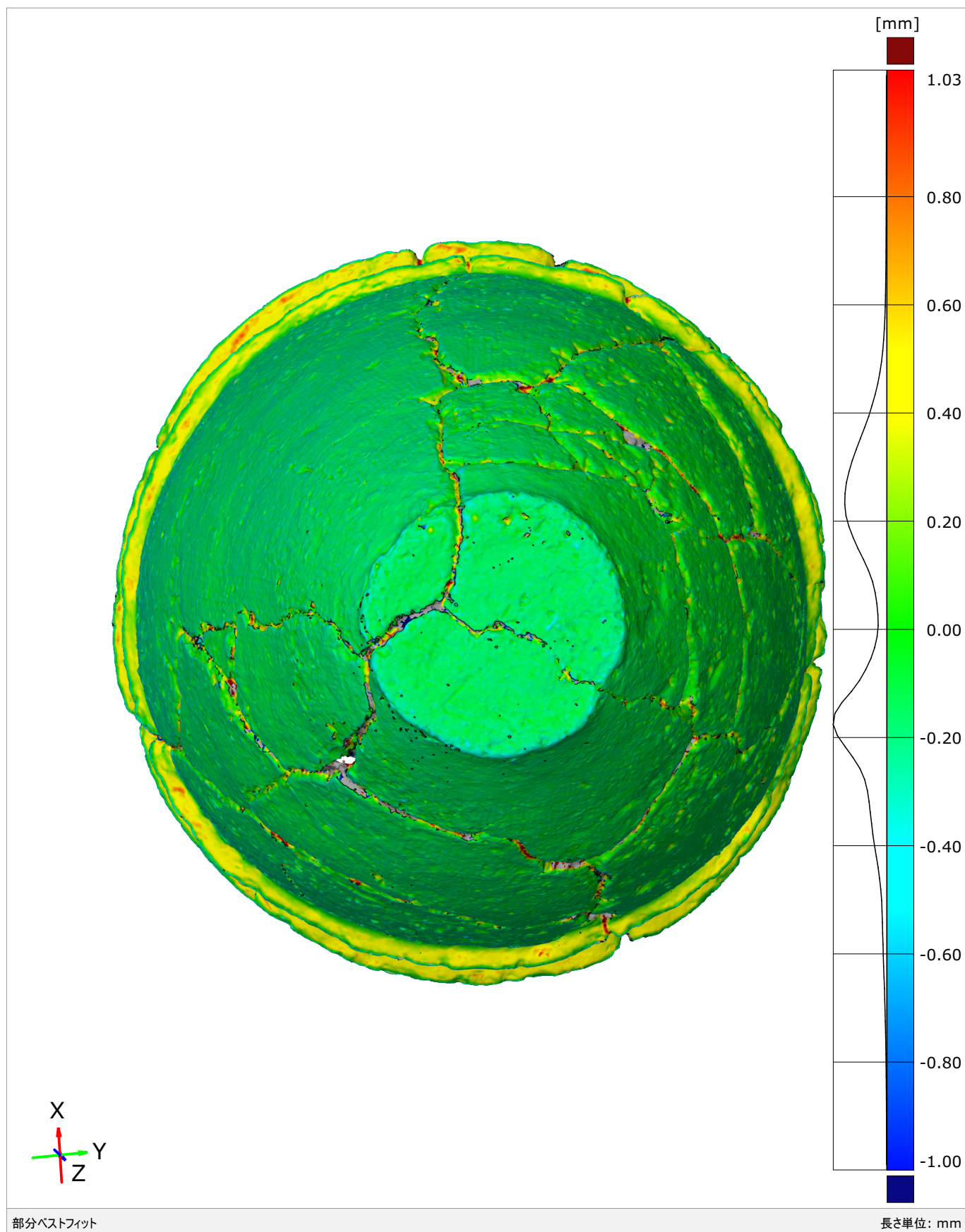

Supplement: S27 Fig — (PDF) [file pone.0270660.s027.pdf]

タイトルなし

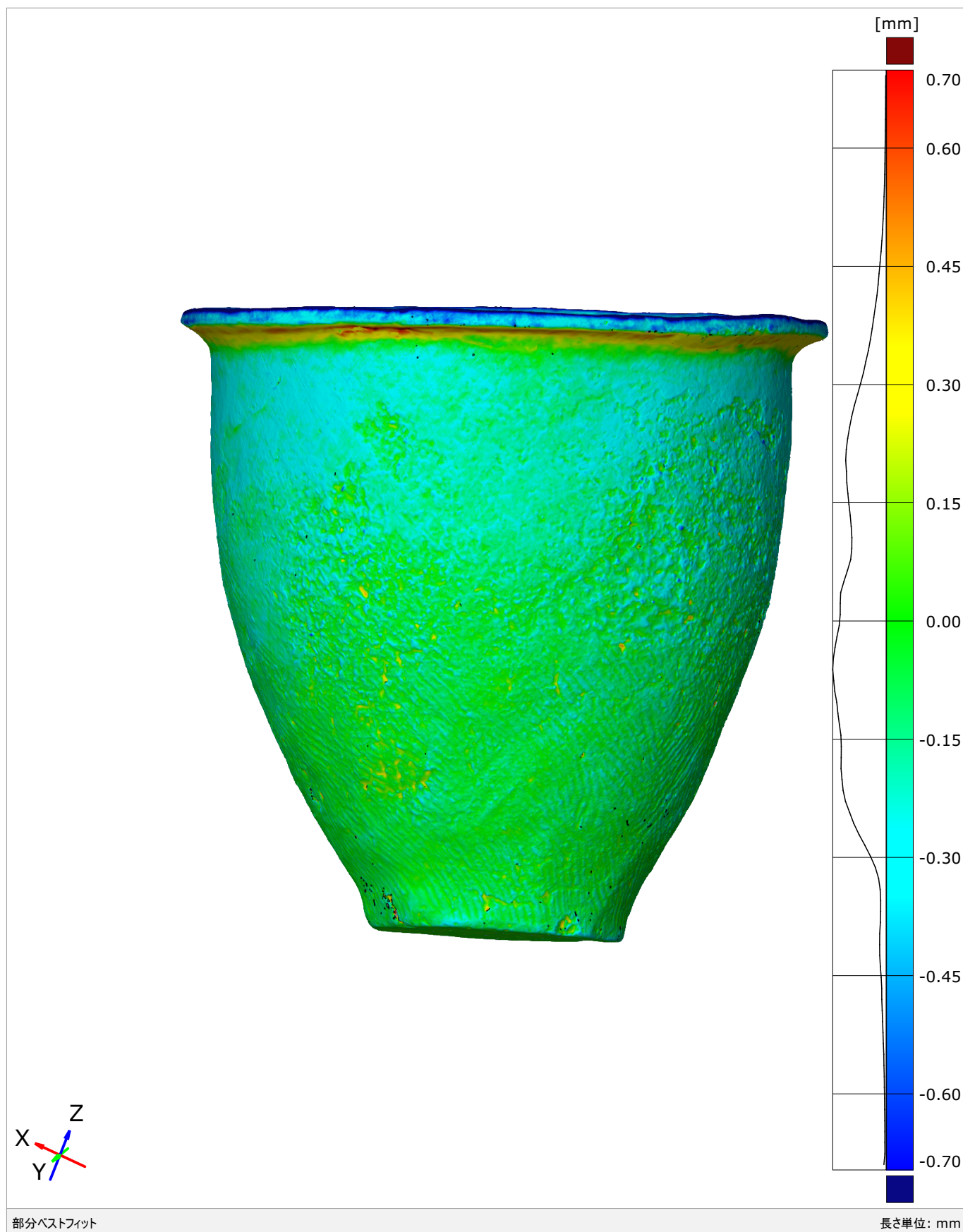

タイトルなし

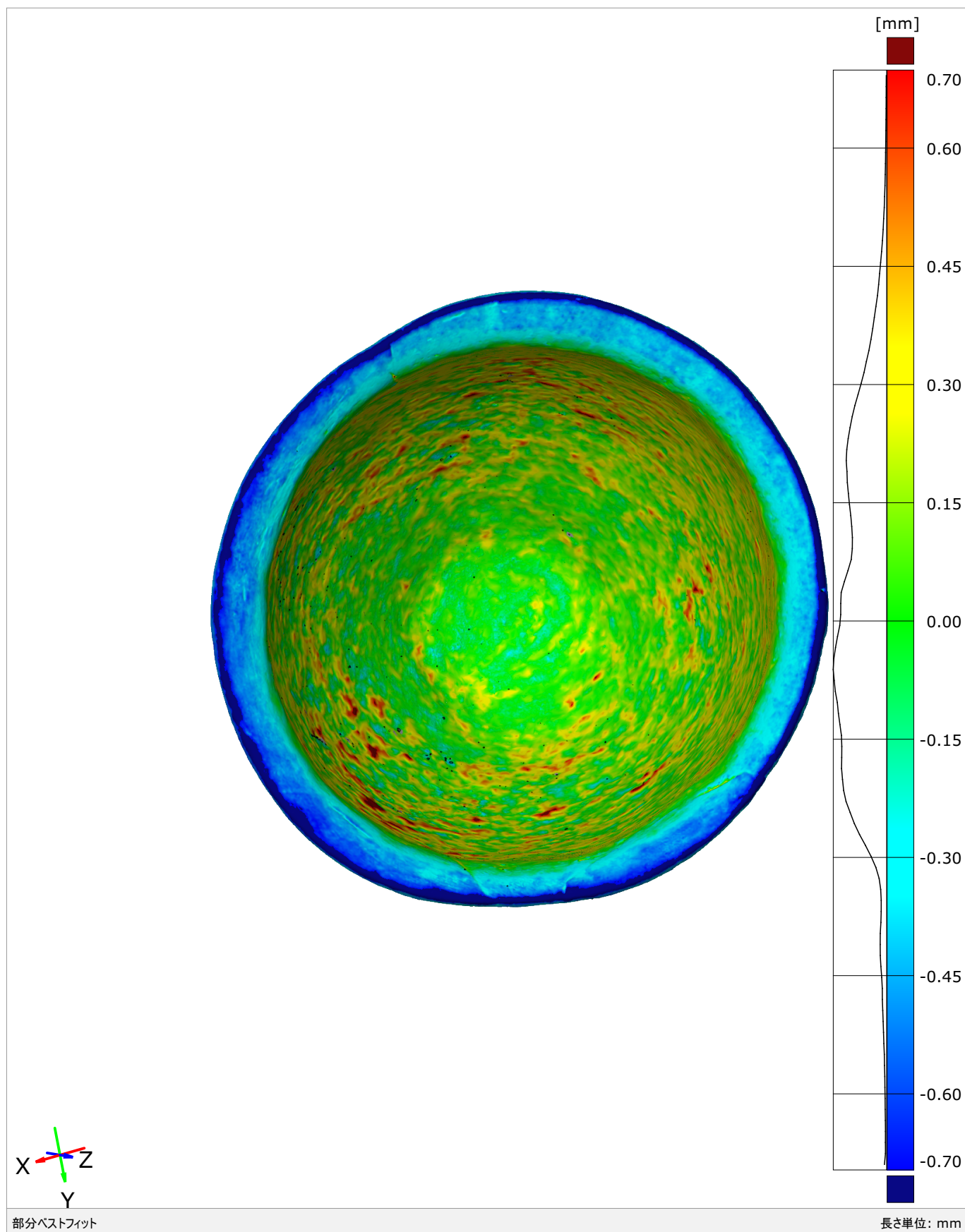

タイトルなし

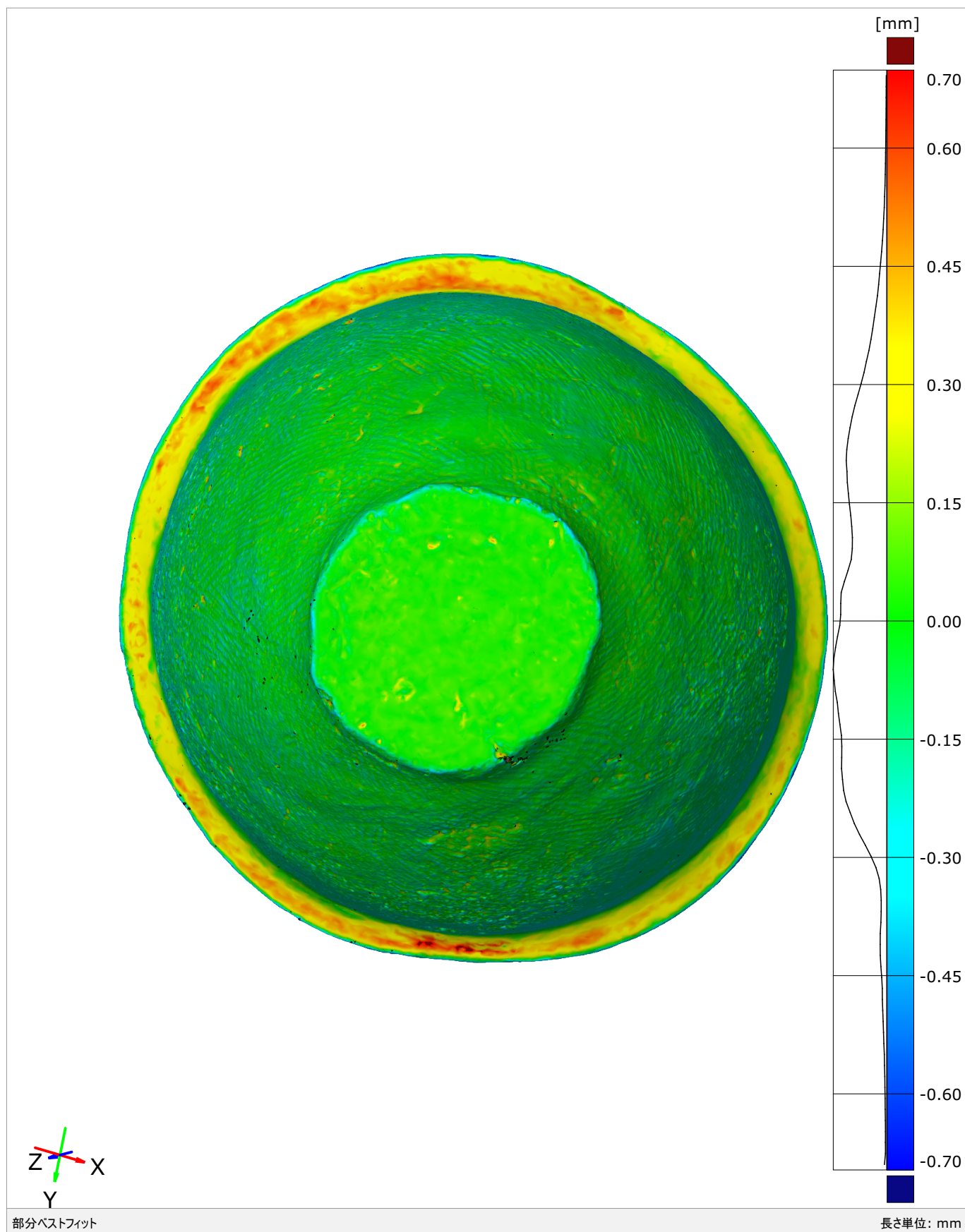

Supplement: S28 Fig — (PDF) [file pone.0270660.s028.pdf]

タイトルなし

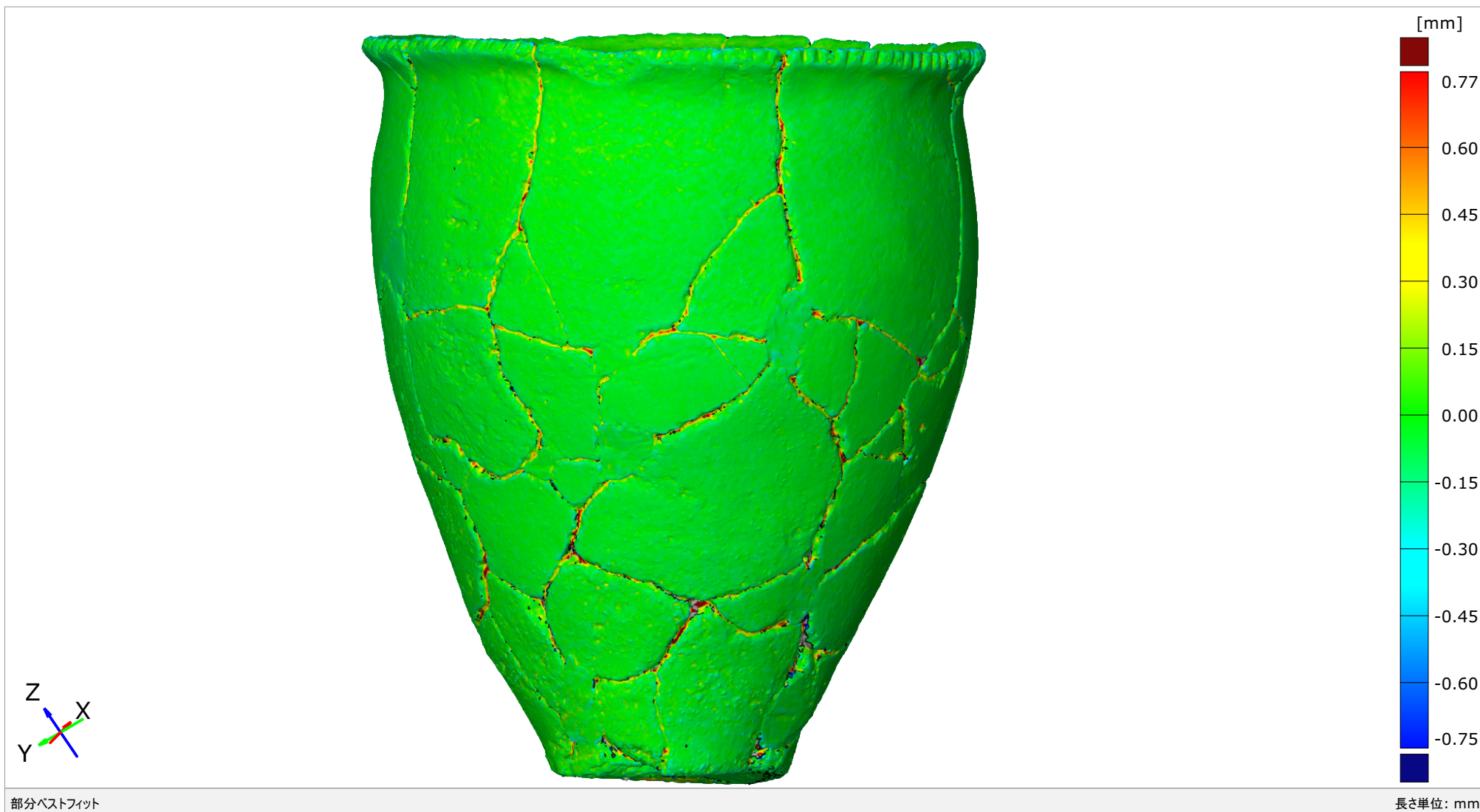

タイトルなし

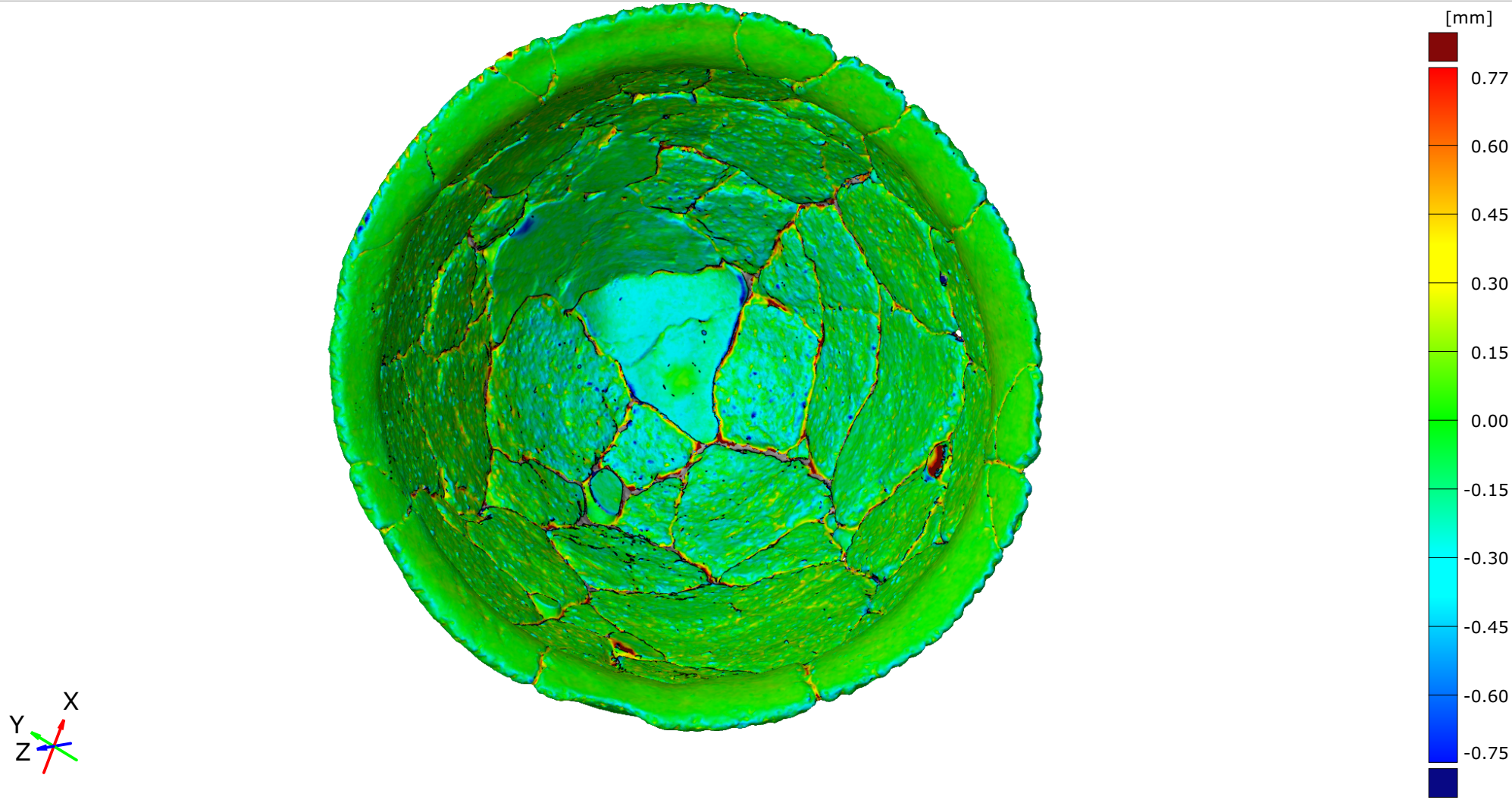

部分ベストフィット

長さ単位: mm

タイトルなし

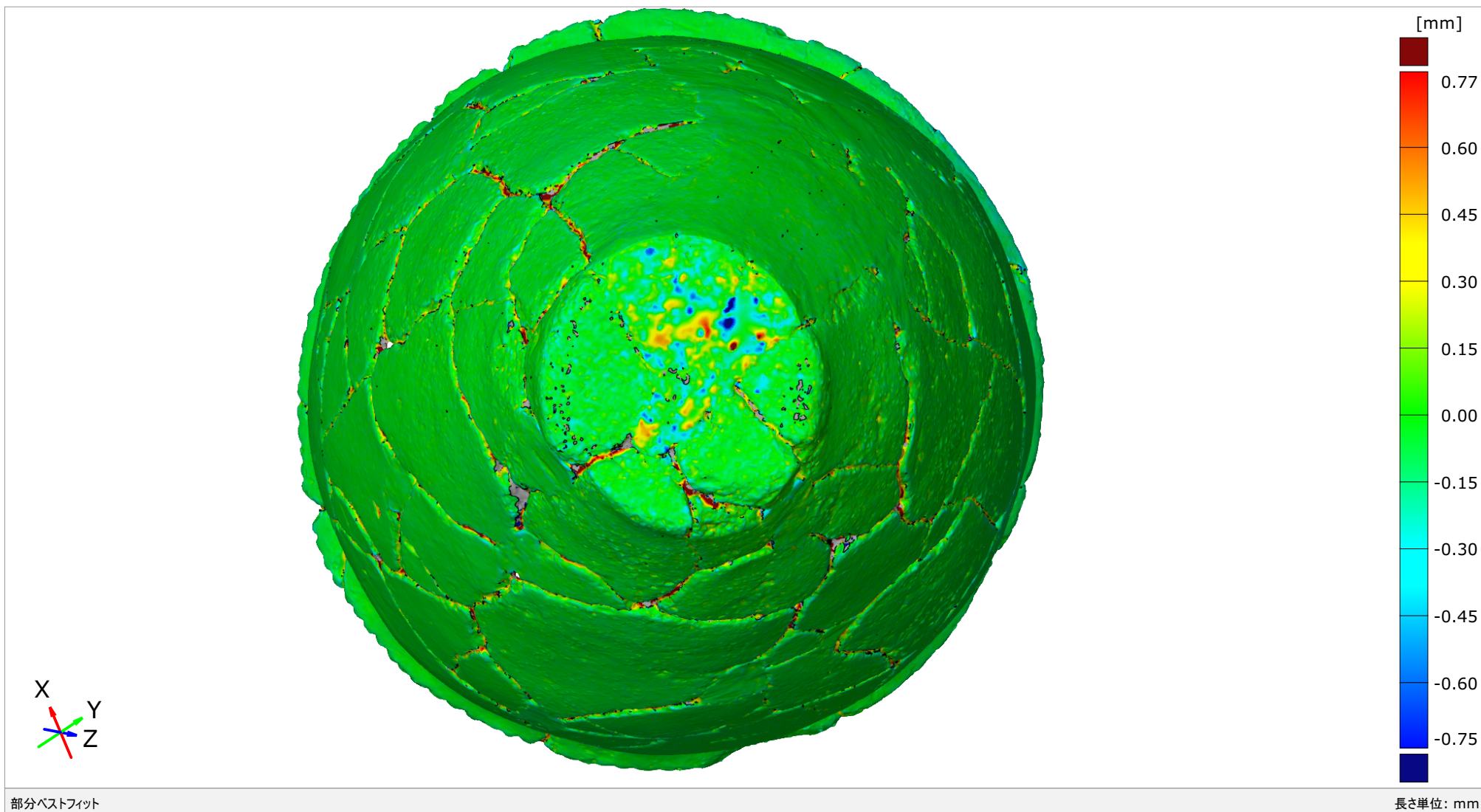

Supplement: S29 Fig — (PDF) [file pone.0270660.s029.pdf]

タイトルなし

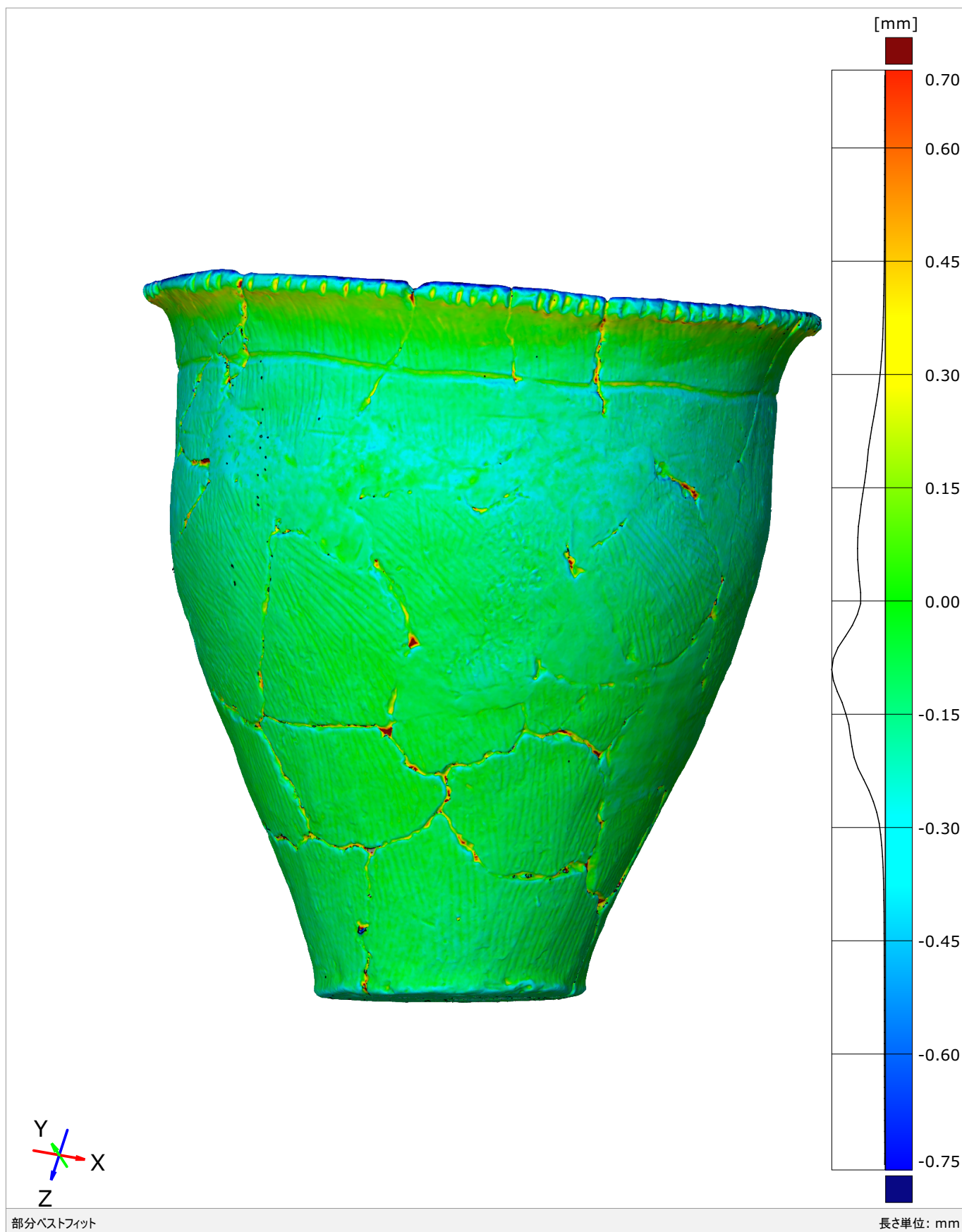

タイトルなし

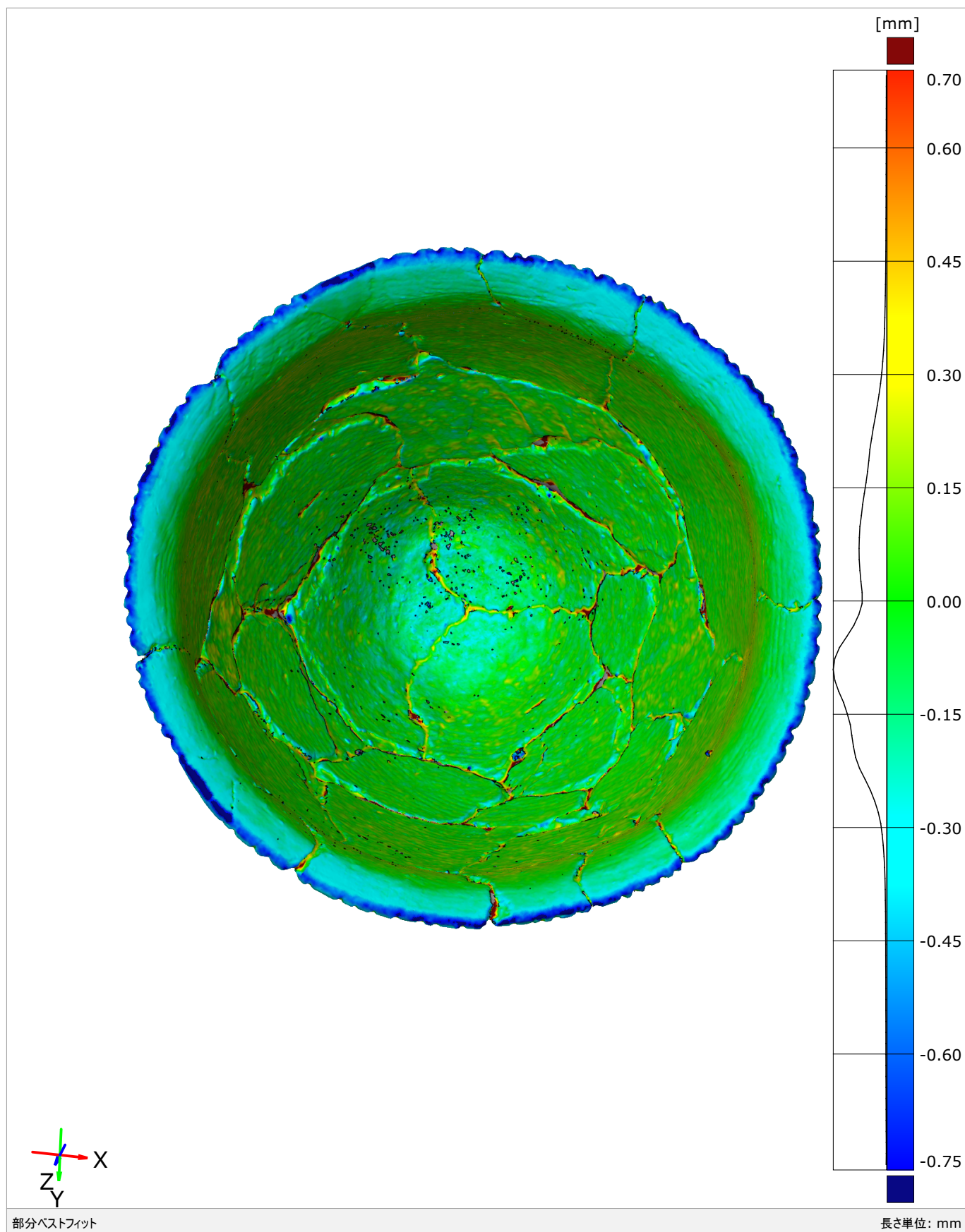

タイトルなし

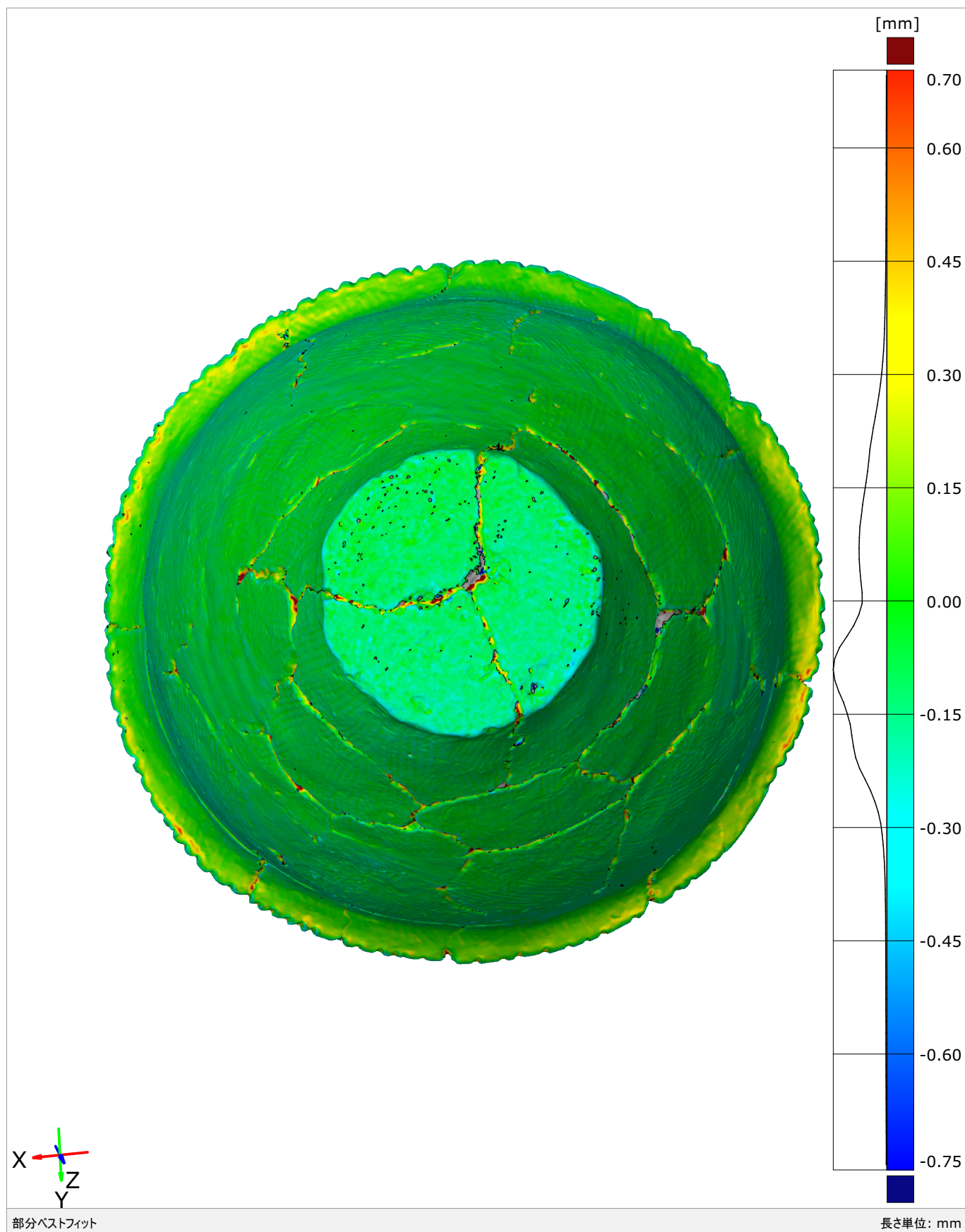

Supplement: S30 Fig — (PDF) [file pone.0270660.s030.pdf]

タイトルなし

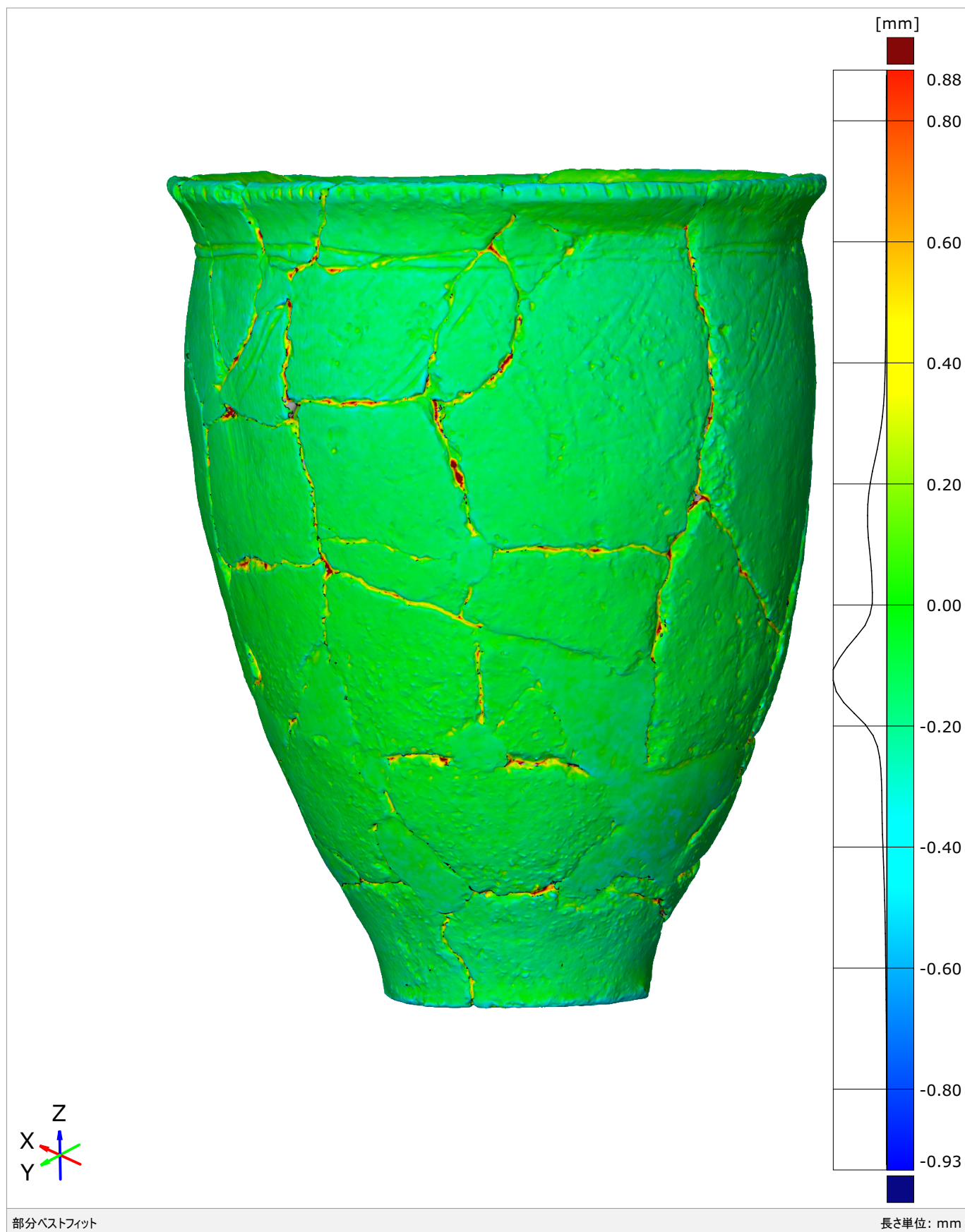

タイトルなし

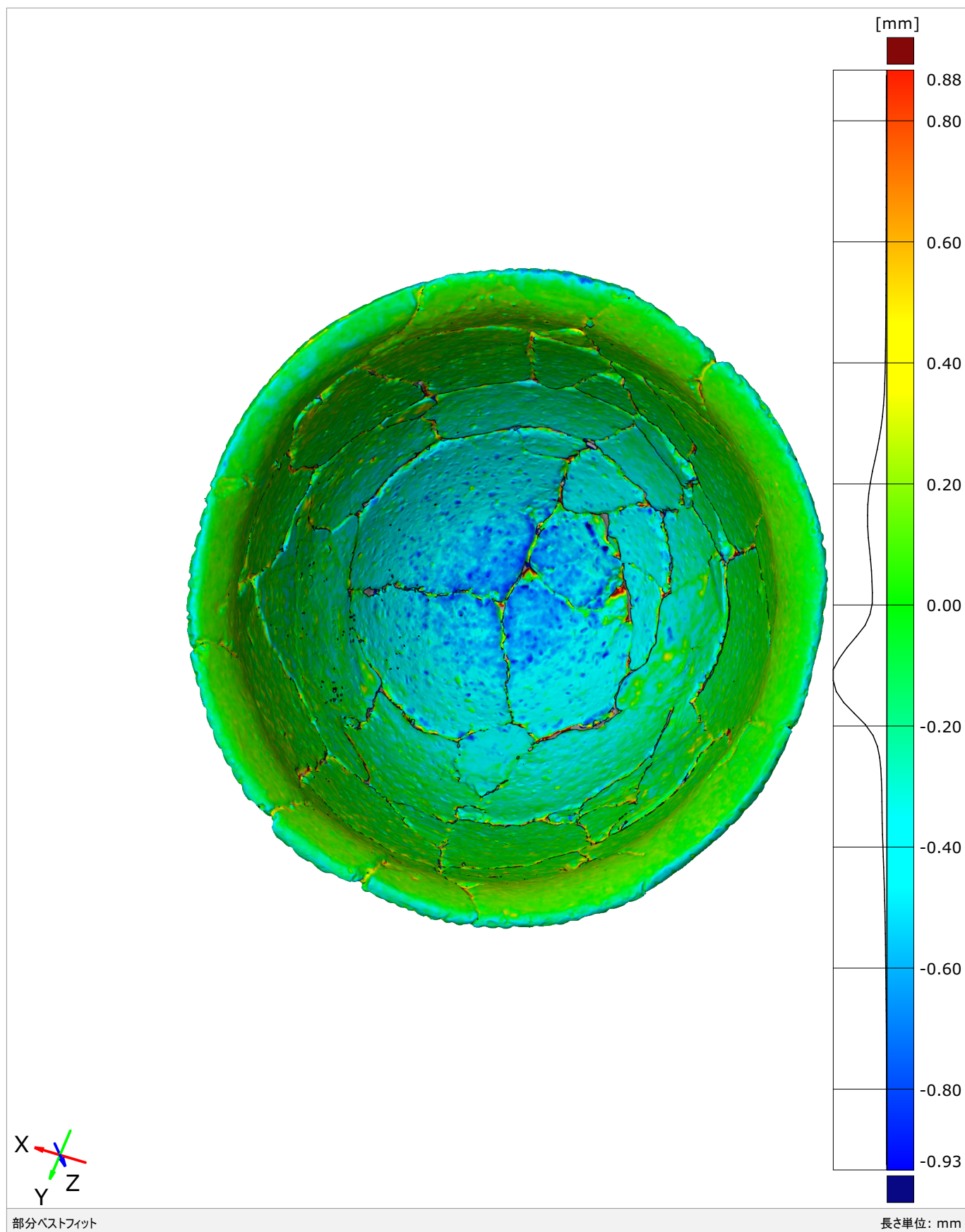

タイトルなし

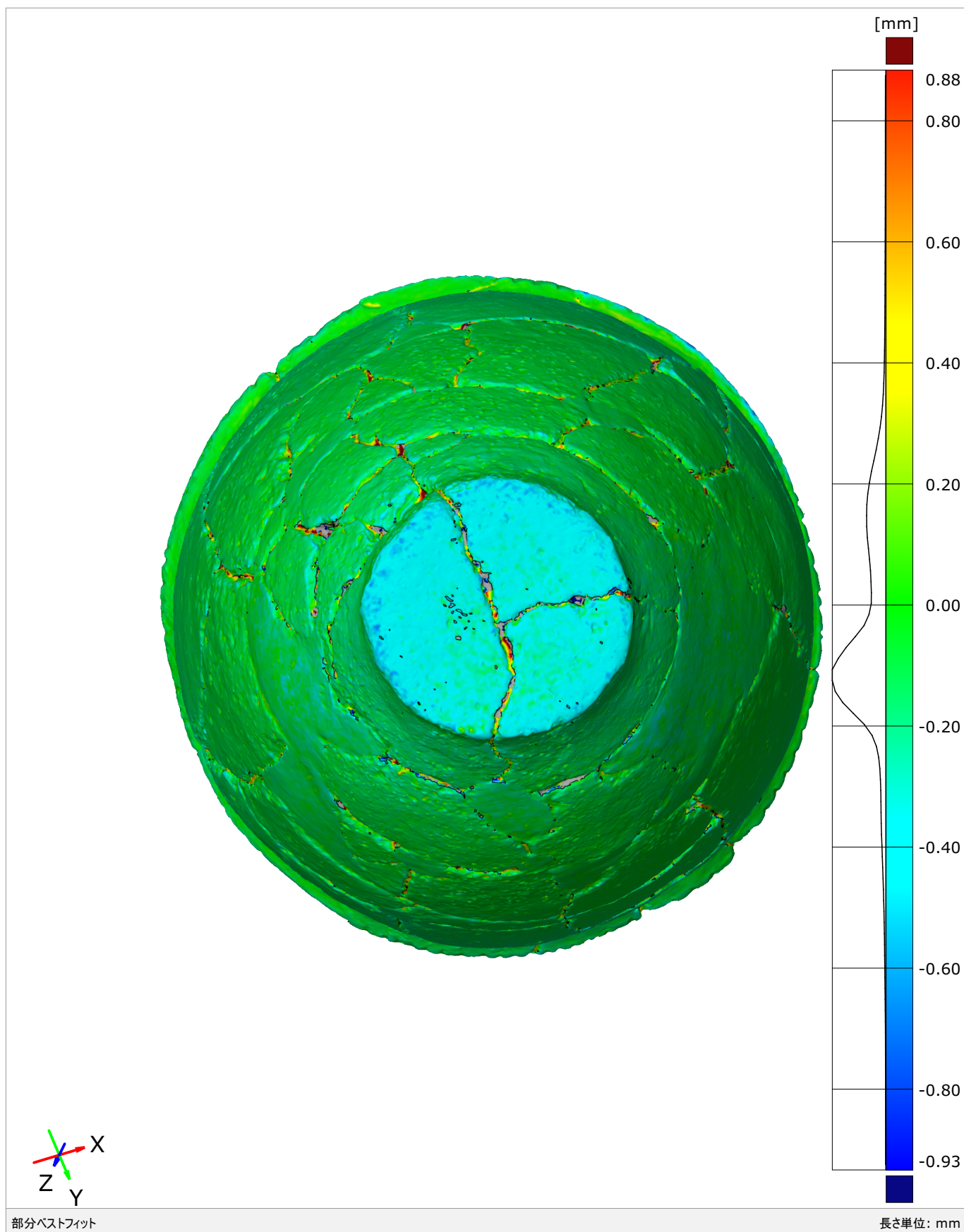

Supplement: S31 Fig — (PDF) [file pone.0270660.s031.pdf]

タイトルなし

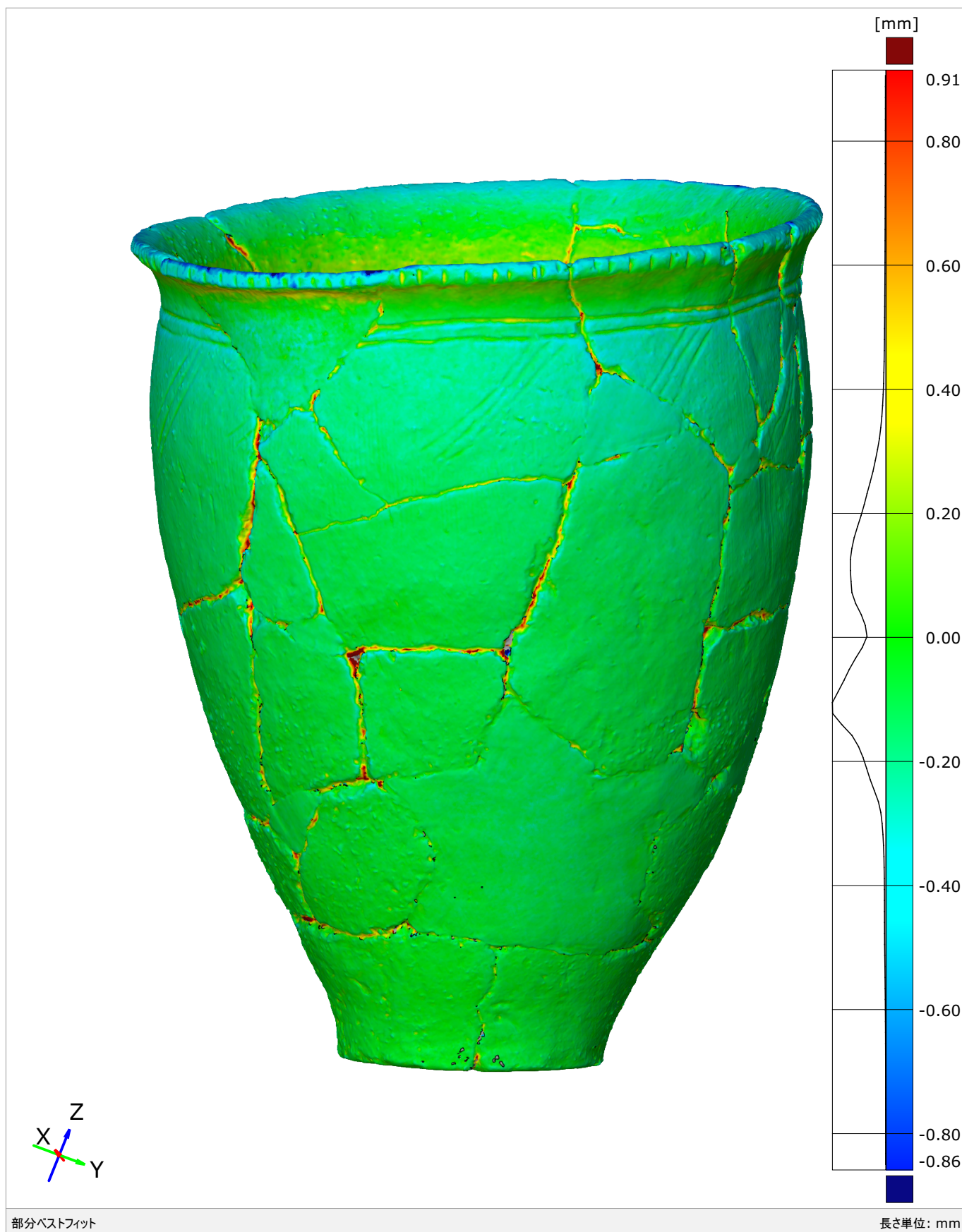

タイトルなし

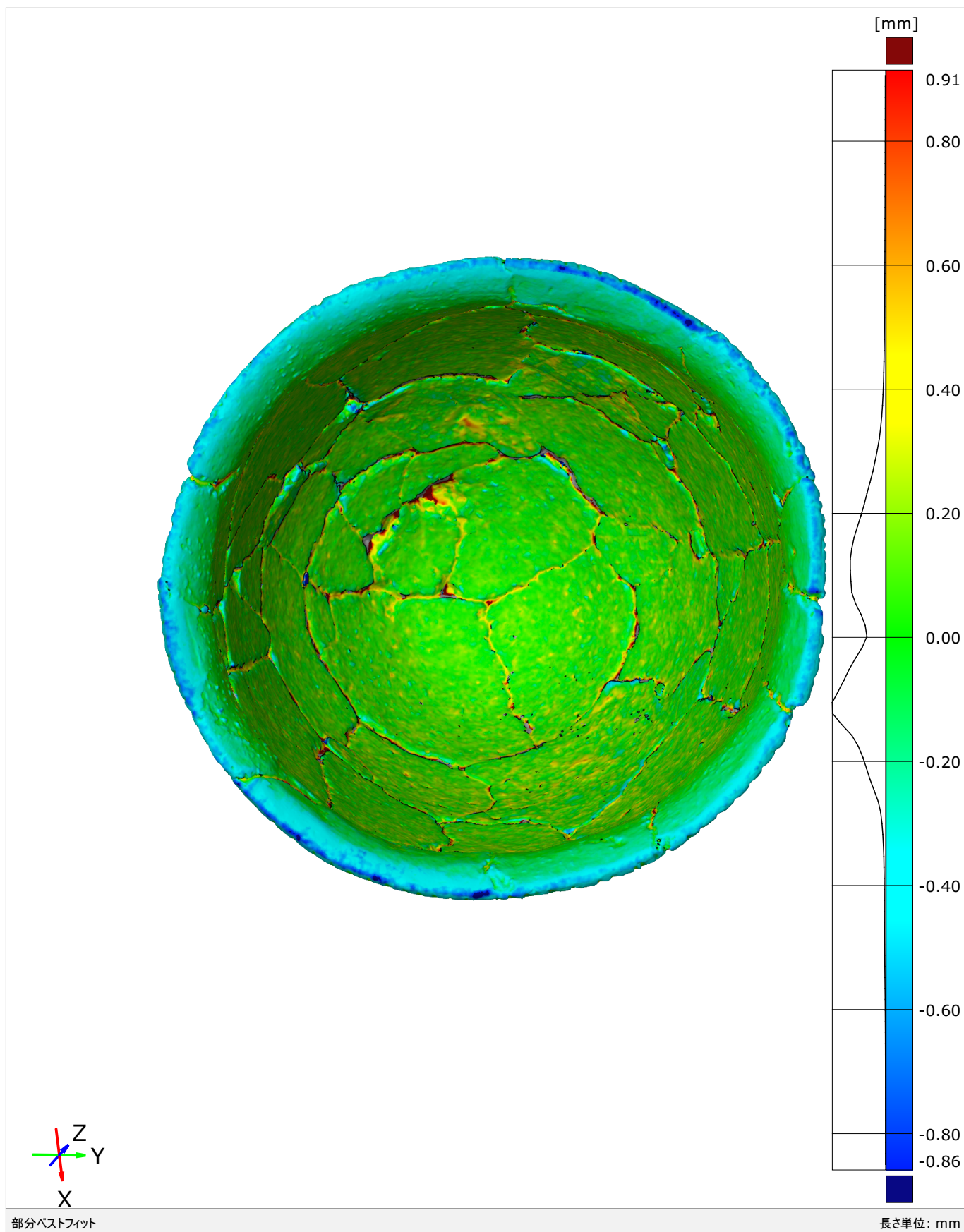

タイトルなし

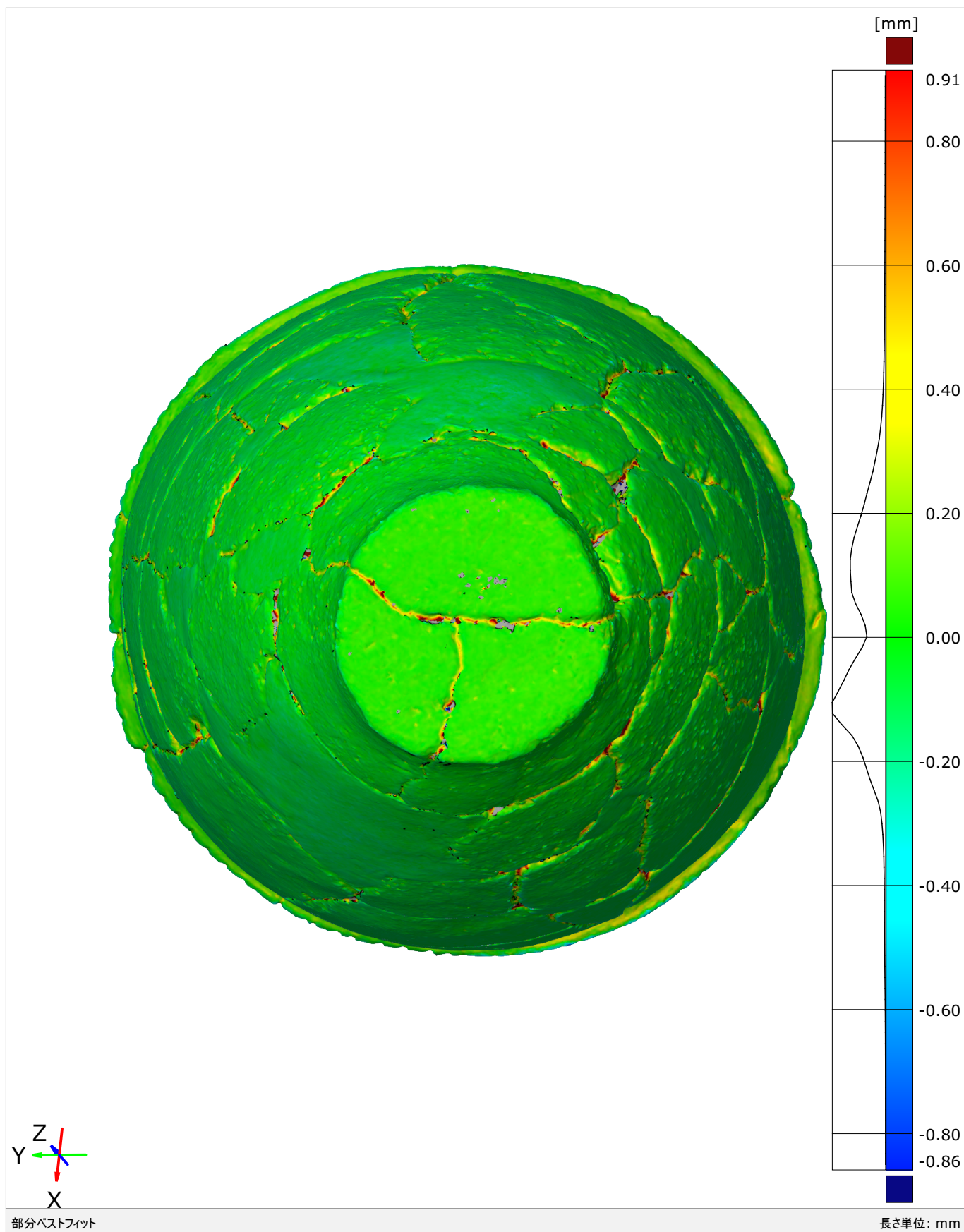

Supplement: S32 Fig — (PDF) [file pone.0270660.s032.pdf]
